# Supplementary material for: Methotrimeprazine is a neuroprotective antiviral in JEV infection via adaptive ER stress and autophagy
Source: EMBO Mol Med. 2024 Jan 2;16(1):185–217. doi: 10.1038/s44321-023-00014-w (PMC10897192; doi:10.1038/s44321-023-00014-w)
Supplement: Supplementary file 1 — Appendix [file 44321_2023_14_MOESM1_ESM.pdf]

Appendix

Contents	Page No.
Appendix Figures S1-S13	2-18
Appendix Table S1: Panel of FDA-approved drugs used in this study	19-20
Appendix Table S2: Percentage distribution of viral RNA and GAPDH mRNA in monosome vs polysome fractions	21
Appendix Table S3: Chemical reagents used in the study	22-28
Appendix Table S4: Primers used in the study	29-31

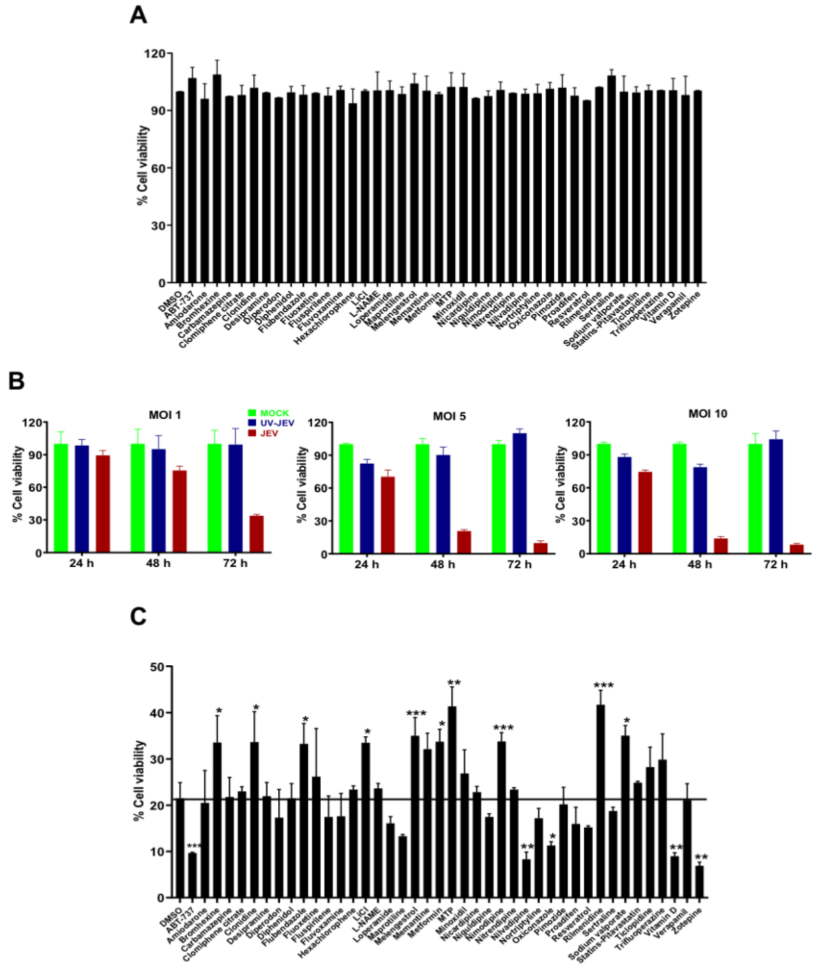

Appendix Figure S1. Effect of FDA-approved drugs on JEV-induced neuronal cell death

A. Neuro2a cells were treated with a panel of 42 FDA-approved drugs at a concentration of 10 µM for 48 h, and cell viability was measured through ATP luminescence using CellTiter-Glo® assay kit. The percentage cell viability was calculated after normalization to DMSO-treated control (n=3).

B, C. Neuro2a cells were mock/JEV/UV-inactivated JEV infected at MOI 1, 5 and 10. Cells were harvested at 24, 48, and 72 h. (C) Neuro2a cells were infected with JEV at MOI 5, and at 2 hpi treated with indicated drugs (10 µM) till 48 hpi. (B-C) The percentage cell viability was calculated by normalization to mock-infected control.

Data information: Data are expressed as means ± SD from biological triplicates. Statistical analysis was performed using one-way ANOVA followed by Dunnett test. *, $P<0.05$; **, $P<0.01$; ***, $P<0.001$; ****, $P<0.0001$.

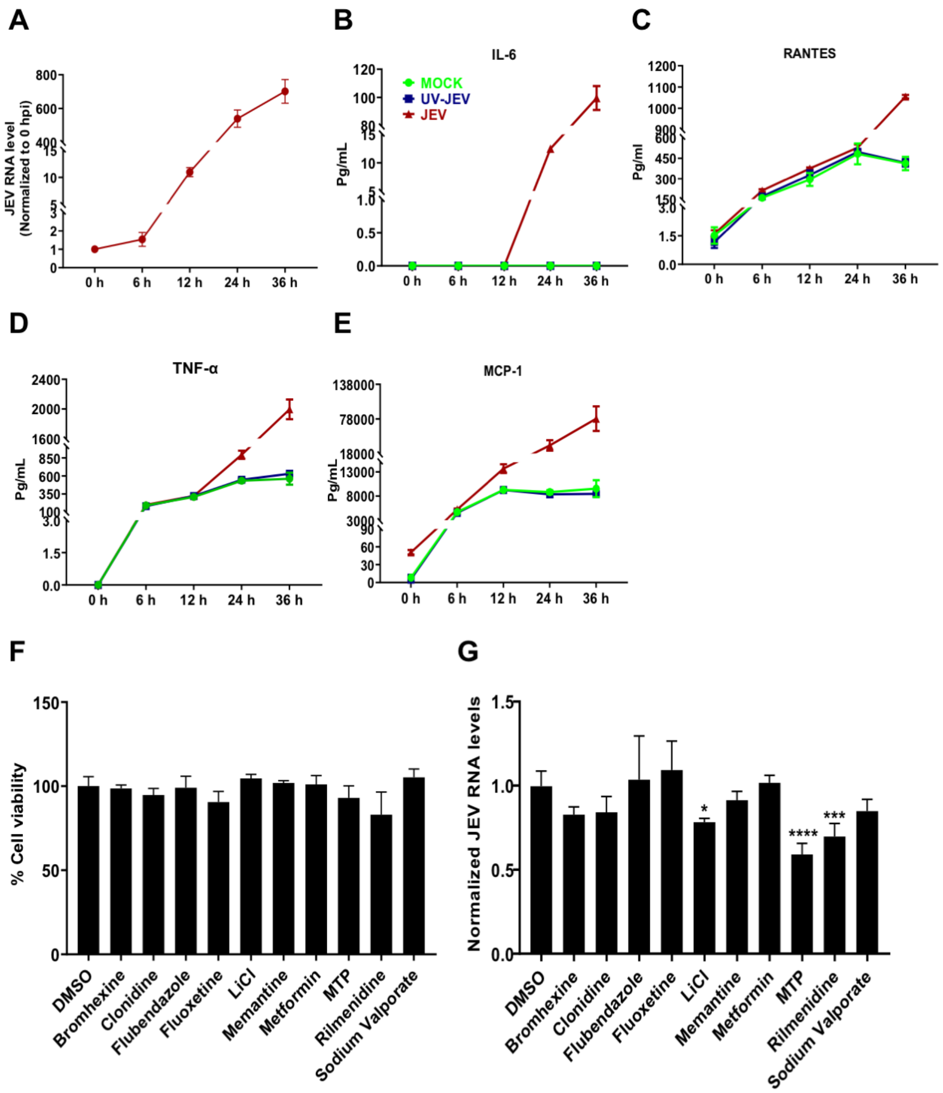

Appendix Figure S2. Effect of FDA-approved drugs on JEV-induced inflammatory response

A. N9 cells were infected with JEV (MOI 1) and harvested at indicated time-points. Relative viral RNA levels were measured by qRT-PCR (n=3).

B-E. N9 cells were mock/JEV/UV-JEV infected (MOI 1) and cytokine levels were quantitated at the indicated time points using CBA assay (n=3).

F. N9 cells were treated with indicated drugs (10 µM) for 24 h, the percentage cell viability normalized to DMSO control was calculated (n=3).

G. N9 cells were mock/JEV (MOI 1) infected, and at 1 hpi treated with DMSO/drugs (10 µM) till 24 hpi. Relative JEV RNA levels were determined using qRT-PCR. Data is represented from three independent experiments (n=9). Data are expressed as means ±

SD, one-way ANOVA followed by Dunnett test. *, P<0.05; **, P<0.01; ***, P<0.001; ****, P<0.0001

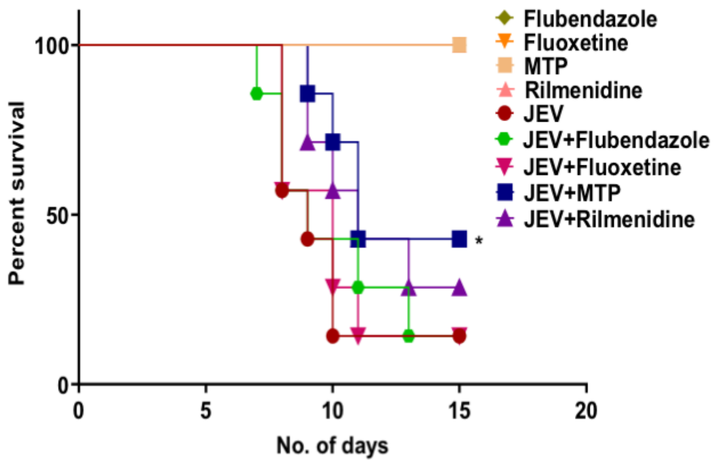

Appendix Figure S3. Antiviral effect of shortlisted drugs in JEV-mouse model

C57BL/6 (3 weeks old) mice were mock/JEV-S3 (10^7 pfu) infected through an i.p. injection, and at 4 hpi, treated with vehicle control (PEG400)/Flubendazole (5 mg/kg)/Fluoxetine (5 mg/kg)/MTP (2 mg/kg)/Rilmenidine (5 mg/kg) by oral gavage at an interval of 24 h till 15 days. Survival curve of mock (n=4)/ Flubendazole (n=4)/ Fluoxetine (n=4)/ MTP (n=4)/ Rilmenidine (n=4)/ JEV (n=7)/ JEV+flubendazole (n=7)/ JEV+fluoxetine (n=7)/ JEV+MTP (n=7)/ JEV+Rilmenidine (n=7) was plotted, a Log-rank (Mantel-Cox) test was used to determine the statistical significance comparing vehicle and drug-treated infected mice group. *, P<0.05.

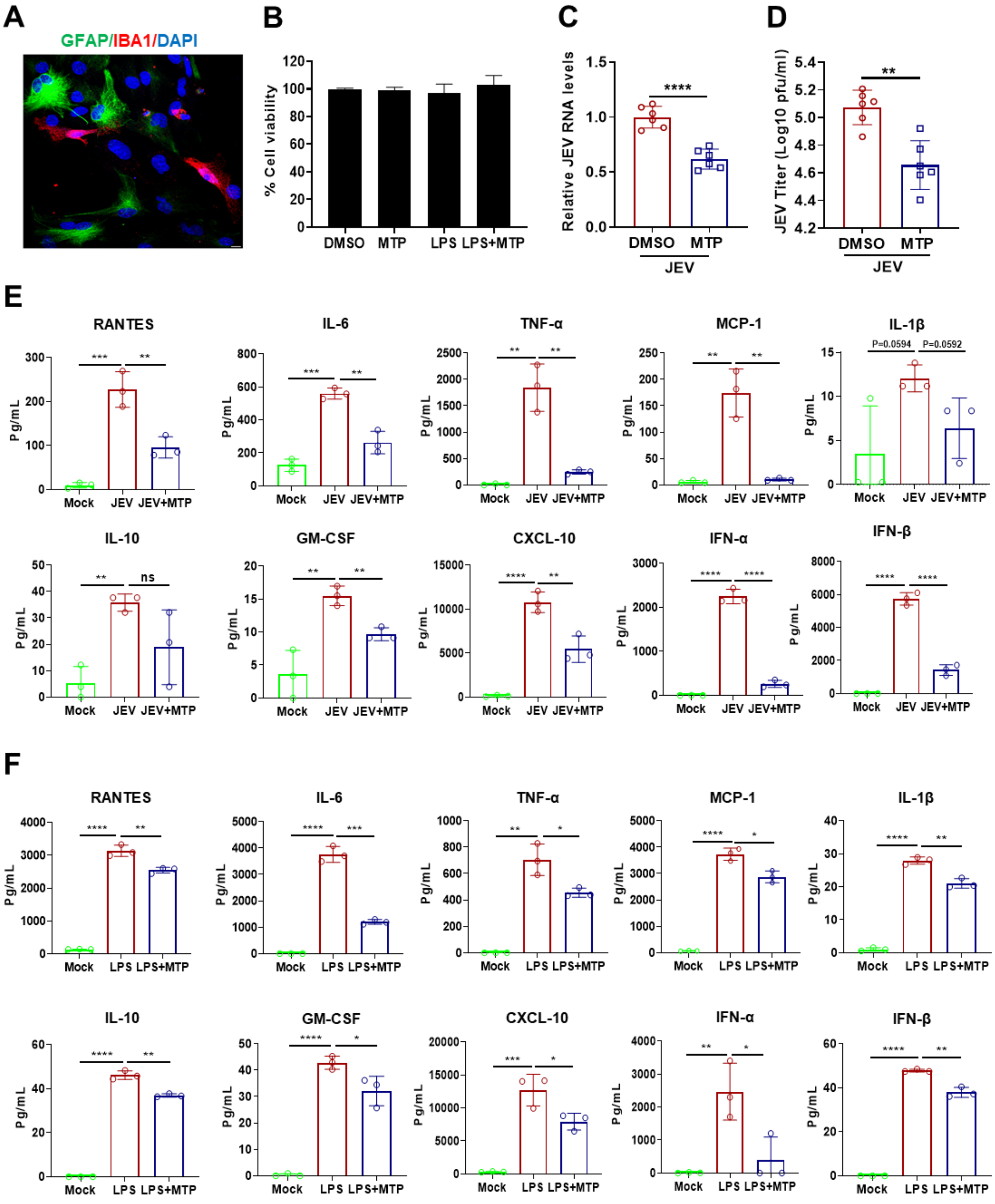
A
GFAP/IBA1/DAPI
B
% Cell viability
DMSO MTP LPS LPS+MTP
C
Relative JEV RNA levels
DMSO MTP
JEV
D
JEV Titer (Log10 pfu/ml)
DMSO MTP
JEV
E
RANTES
IL-6
TNF-α
MCP-1
IL-1β
P=0.0594 P=0.0592
IL-10
GM-CSF
CXCL-10
IFN-α
IFN-β
Pg/mL
Mock JEV JEV+MTP
F
RANTES
IL-6
TNF-α
MCP-1
IL-1β
IL-10
GM-CSF
CXCL-10
IFN-α
IFN-β
Pg/mL
Mock LPS LPS+MTP

Appendix Figure S4. MTP inhibits the secretion of proinflammatory cytokines from JEV infected/LPS-treated mixed glial cells

A. Mouse primary mixed glial cells were isolated from P2 pups and immunostained with GFAP and IBA1 antibodies. Scale bar, 10 µm.

B. Mixed glial cells were treated with DMSO/MTP (10 µM)/LPS (1 µg/ml)/LPS+MTP for 24 h. Percentage cell viability was measured by MTT assay and normalized to DMSO treated control (n=3).

C, D. Mixed glial cells were mock/JEV (MOI 1) infected, at 1 hpi, treated with either DMSO or MTP (10 µM) till 24 h. (C) Viral transcript levels were determined by qRT-PCR. Graph shows the relative expression levels of JEV RNA normalized to DMSO-treated control. Data is plotted from two independent experiments (n=6). (D) Culture supernatant was used to determine virus titre using plaque assay. Data represents values obtained from two independent experiments (n=6).

E, F. Mixed glial cells were infected with JEV (MOI 1) for 1 h then treated with DMSO/MTP (10 µM) for 24 h (E), or were treated with DMSO/ LPS (1 µg/ml)/LPS+MTP for 24 h (F). Culture supernatants were harvested and cytokine levels were quantitated by CBA using flow cytometry. Data were analysed with LEGENDplexTM Multiplex assay software. Data shows values from one representative experiment (n=3). Similar trends were seen in two independent experiments.

Data information: All data are expressed as means ± SD, unpaired Student t-test. *, $P<0.05$; **, $P<0.01$; ***, $P<0.001$; ****, $P<0.0001$, ns; non-significant.

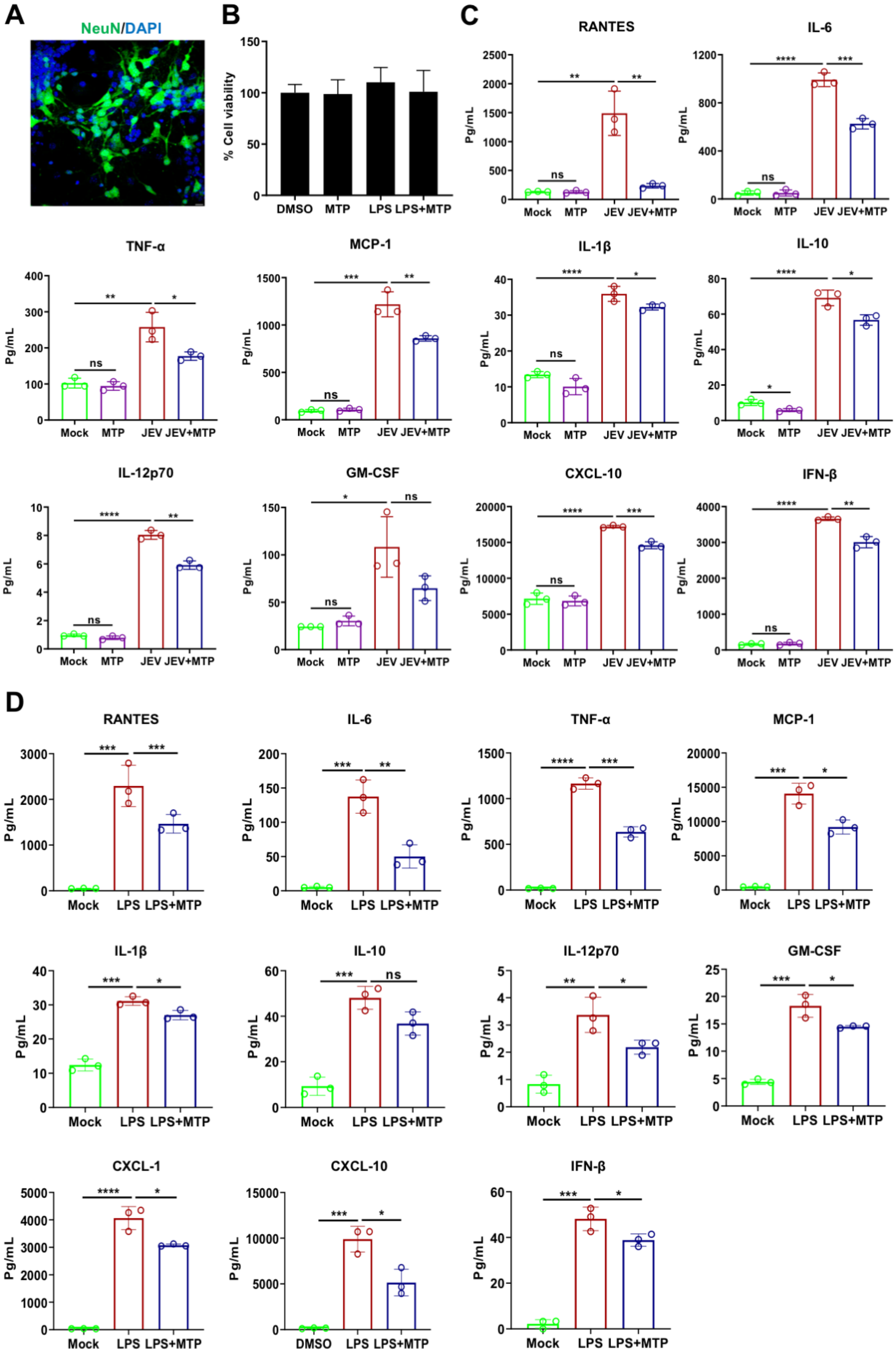
A
NeuN/DAPI
B
% Cell viability
150
100
50
0
DMSO
MTP
LPS
LPS+MTP
C
RANTES
IL-6
TNF-α
MCP-1
IL-1β
IL-10
IL-12p70
GM-CSF
CXCL-10
IFN-β
Pg/mL
Mock
MTP
JEV
JEV+MTP
ns
*
**
***
****
D
RANTES
IL-6
TNF-α
MCP-1
IL-1β
IL-10
IL-12p70
GM-CSF
CXCL-1
CXCL-10
IFN-β
Pg/mL
Mock
DMSO
LPS
LPS+MTP

Appendix Figure S5. MTP inhibits the secretion of proinflammatory cytokines from JEV infected/LPS-stimulated primary cortical neurons

A. Cortical neurons were isolated from pregnant mice (E16.5), and immunostained with NeuN antibody. Scale bar, 10 µm.

B. Cortical neurons were treated with DMSO/MTP (10 µM)/LPS (1 µg/ml)/LPS+MTP for 24 h. Percentage cell viability was measured by MTT assay (n=3).

C, D. Cortical neurons were infected with JEV (MOI 1), and at 1 hpi treated with DMSO/MTP (10 µM) till 24 h (C), or were treated with DMSO/ LPS (1 µg/ml)/LPS+MTP for 24 h (D). Culture supernatants were collected, and cytokine levels were quantitated by CBA using flow cytometry. Data were analyzed with LEGENDplexTM Multiplex assay software, and significance was compared by unpaired Student t-test. Data shows values from one representative experiment (n=3). Similar trends were seen in two independent experiments.

Data information: All data are expressed as means ± SD*, $P<0.05$; **, $P<0.01$; ***, $P<0.001$; ****, $P<0.0001$, ns; non-significant.

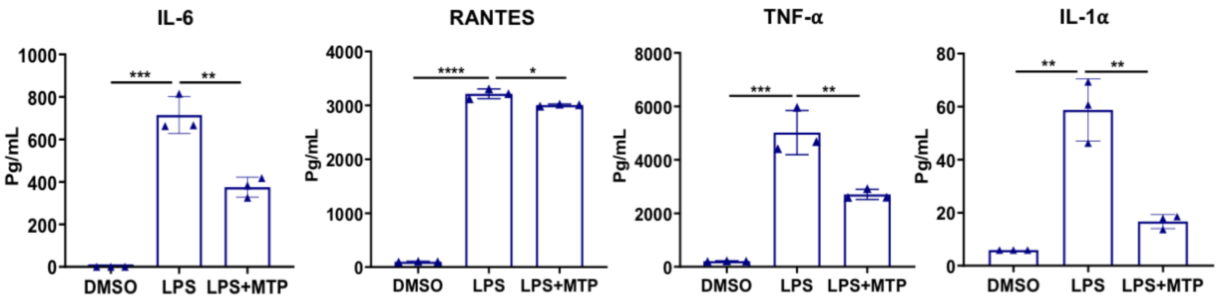

Appendix Figure S6. MTP shows anti-inflammatory responses in LPS treated microglial cells

N9 cells were treated with DMSO/LPS (1 µg/ml)/LPS+MTP (10 µM) for 24 h, and cytokine levels were quantified from the supernatant (n=3). Statistical significance was determined by unpaired Student t-test. *P<0.05; **, P<0.01; ***, P<0.001; ****, P<0.0001.

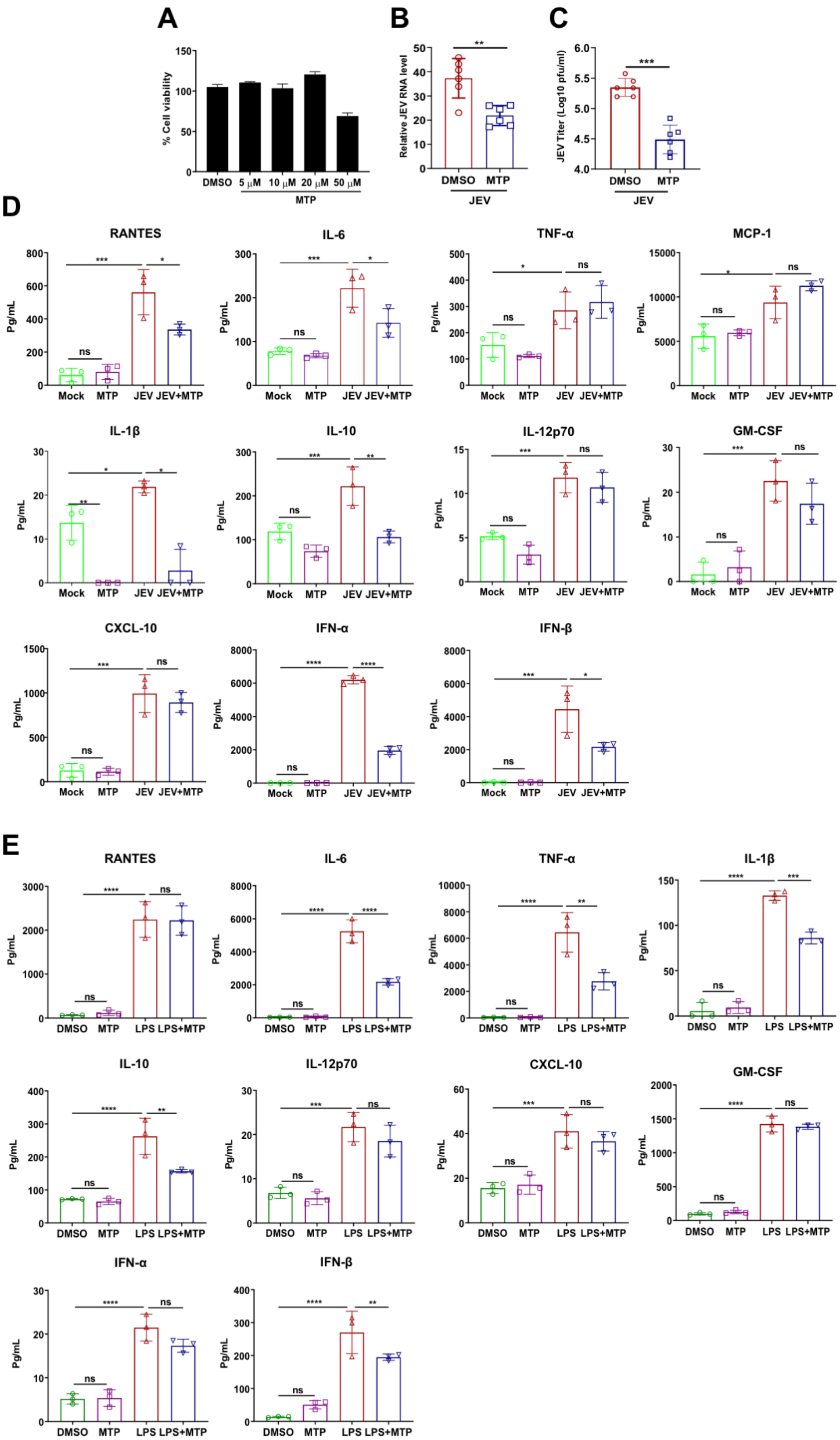
A
B
C
D
E
% Cell viability
DMSO 5 μM 10 μM 20 μM 50 μM
MTP
Relative JEV RNA level
JEV Titer (Log10 pfu/ml)
DMSO MTP
JEV
RANTES
IL-6
TNF-α
MCP-1
IL-1β
IL-10
IL-12p70
GM-CSF
CXCL-10
IFN-α
IFN-β
Pg/mL
Mock MTP JEV JEV+MTP
DMSO MTP LPS LPS+MTP
ns

Appendix Figure S7. MTP inhibits the secretion of proinflammatory cytokines from JEV infected BMDMs

A. BMDMs were treated with DMSO/MTP at indicated concentration for 24 h, MTT assay was used to calculate % cell viability.

B-D. BMDMs were mock/JEV (MOI 2) infected for 1 h, followed by DMSO/MTP (10 µM) treatment till 24 hpi. (B) Viral transcript levels were measured using qRT-PCR and normalized to DMSO-treated infected control (n=6). (C) Virus titer in the culture supernatant was determined by plaque assays (n=6), unpaired Student t-test. (D) Culture supernatant was used for the quantitation of cytokine levels by flow cytometry-based CBA assay (n=3). Similar trends were seen in two independent experiments.

E. BMDMs were treated with DMSO/MTP (10 µM)/LPS (1 µg/ml)/LPS+MTP for 24 h, supernatant was harvested and used for the quantitation of cytokine levels (n=3).

Data information: All data were expressed as means ± SD, unpaired Student t-test, *, P<0.05; **, P<0.01; ***, P<0.001; **** P < 0.0001, ns; non-significant.

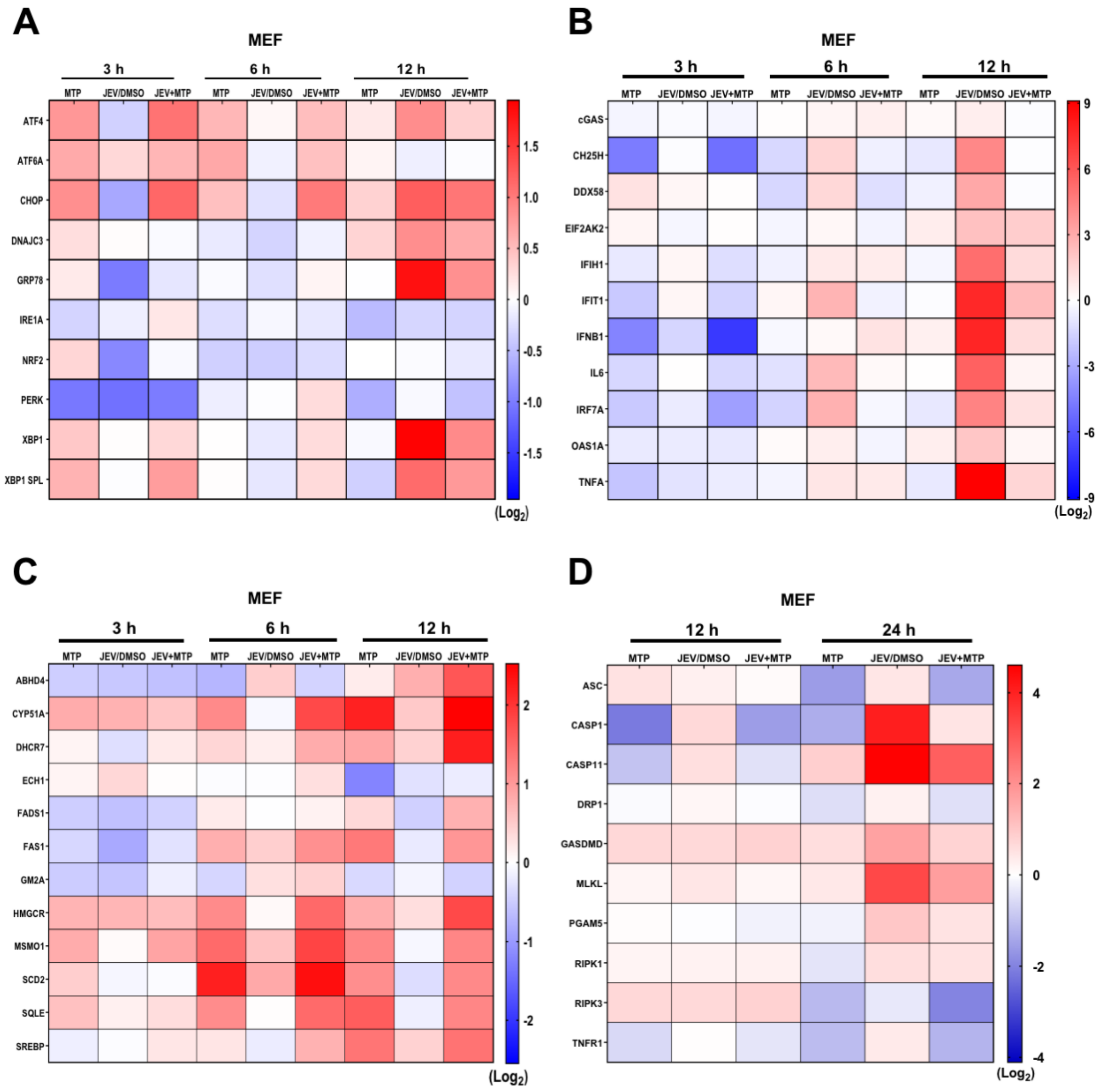

Appendix Figure S8. MTP induces adaptive ER stress

MEFs were infected with JEV at MOI 1, and at 1 hpi, cells were treated with DMSO/MTP (10 μM) for the indicated time points. RNA was used for the quantitation of ER-stress (A), innate immune/inflammatory pathway (B), cholesterol metabolic pathway genes (C), and cell death genes (D). Heatmap showing relative gene expression levels normalized to DMSO control, represented as mean from two independent experiments (n=6).

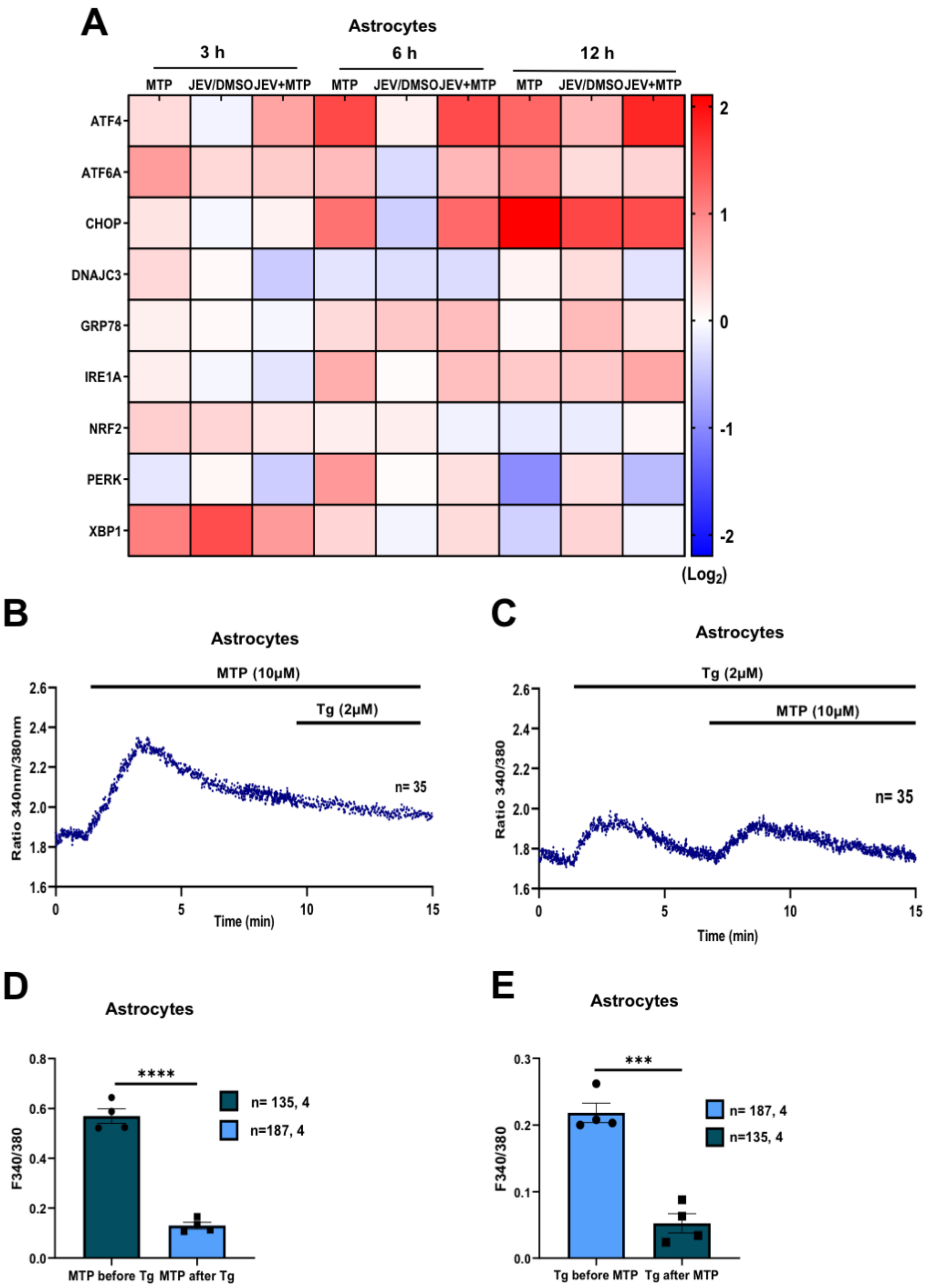

Appendix Figure S9. MTP activates ER stress and dysregulates ER calcium homeostasis in astrocytes

A. Astrocytes were infected with JEV (MOI 1), and at 1 hpi treated with DMSO/MTP (10 μM) for the indicated time points. RNA levels of ER-stress genes were quantitated by qRT-PCR. Heatmap showing relative gene expression levels normalized to DMSO control, represented as mean (n=3).

B. Representative Ca^{2+} imaging trace of experiments where Astrocytes were stimulated with 10 μM MTP in absence of extracellular Ca^{2+} followed by addition of 2 μM thapsigargin (Tg). Here, "n=35" denotes the number of cells in that particular trace.
C. Representative Ca^{2+} imaging trace of experiments where cells were stimulated first with 2 μM Tg to deplete ER Ca^{2+} stores, followed by addition of 10 μM MTP in absence of extracellular Ca^{2+}. Here, "n=35" denotes the number of cells in that particular trace.
D. Quantitation of MTP (10 μM) induced ER Ca^{2+} stores depletion before and after the addition of 2 μM Tg. 135 and 187 cells from 4 independent imaging dishes were analysed for the two conditions, respectively.
E. Quantitation of Tg, (2 μM) induced ER Ca^{2+} stores depletion before and after the addition of 10 μM MTP. 187 and 135 cells from 4 independent imaging dishes were analysed for the two conditions, respectively. ("n = x, y" where "x" denotes total number of cells imaged and "y" denotes number of traces recorded).

Data information: Data presented are mean ± S.E.M, and statistical significance was calculated by unpaired Student t-test. ***, $P<0.001$; **** $P < 0.0001$

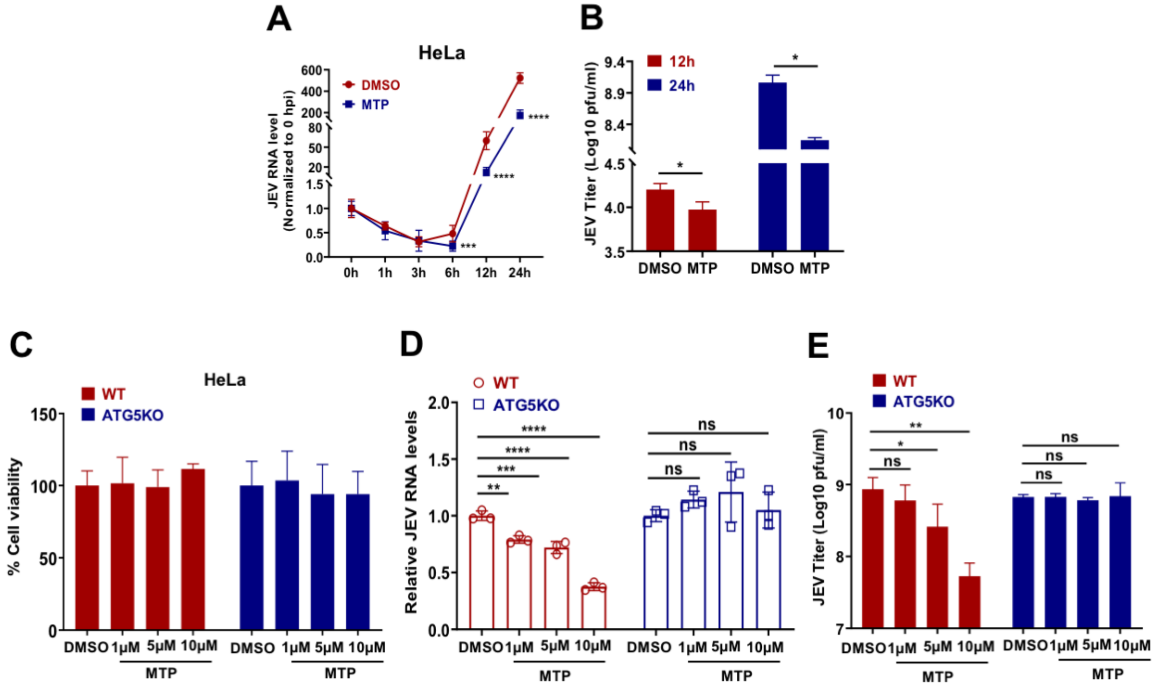

Appendix Figure S10. Antiviral effect of MTP is autophagy dependent in HeLa cells

A, B. HeLa cells were infected with JEV (MOI 1), and at 1 hpi treated with DMSO/MTP (10 µM). (A) Cells were harvested at the indicated hpi and viral RNA levels were quantified using qRT-PCR. Data represents values obtained from two independent experiments (n=6). (B) Culture supernatant was used for the determination of virus titer using plaque assay. Data is plotted from two independent experiments (n=6). Statistical significance was calculated by unpaired Student t-test.

C. WT and ATG5 KO HeLa cells were treated with indicated concentrations of MTP for 24 h, and the percentage cell viability was measured and normalized to respective DMSO treated controls (n=3).

D, E. WT and ATG5 KO HeLa cells were infected with JEV at MOI 1, and at 1 hpi treated with MTP at indicated concentrations. (D) Cells were harvested at 24 hpi and the relative viral RNA levels were quantitated using qRT-PCR, and plotted after normalization to respective DMSO-treated control. (E) Culture supernatant was collected and virus titer was determined by plaque assay. Data represents values from three independent experiments (n=9). One-way ANOVA test followed by Dunnett test was used for the determination of statistical significance.

Data information: All data are expressed as means ± SD **, P<0.01; ***, P<0.001; **** P < 0.0001, ns; non-significant.

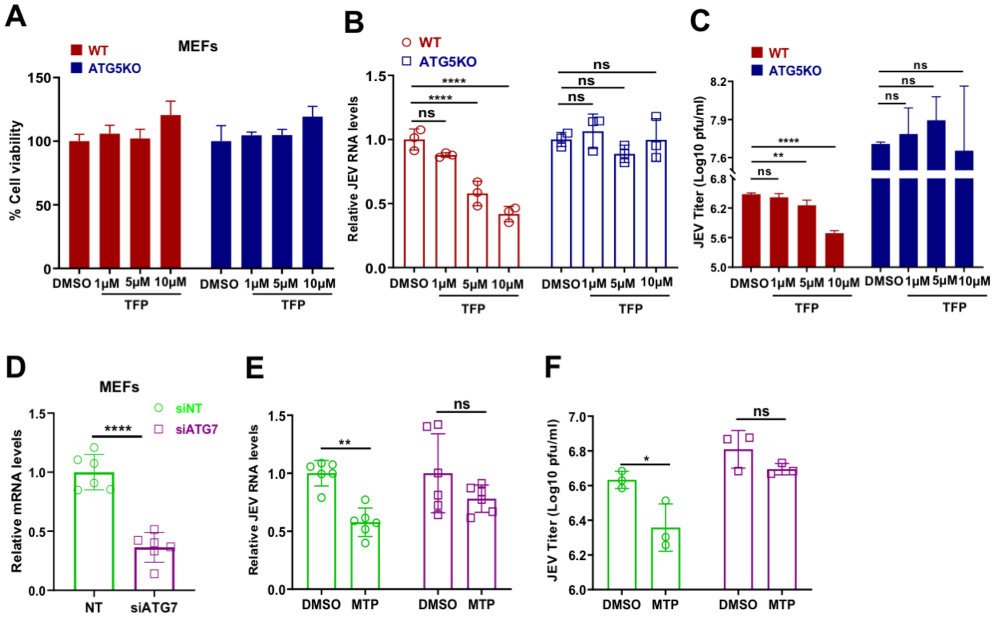

Appendix Figure S11: Antiviral effect of phenothiazines is autophagy dependent in MEFs

A. WT and ATG5 KO MEFs were treated with indicated concentrations of TFP for 24 h, MTT assay was used to calculate percentage cell viability, and normalized to respective DMSO treated controls (n=3).

B, C. WT and ATG5 KO MEFs were infected with JEV (MOI 1) for 1 h. Post-infection, cells were treated with TFP at indicated concentrations till 24 h. (B) Viral RNA levels were quantitated using qRT-PCR, and plotted after normalization to respective DMSO-treated control. Data is plotted from three independent experiments (n=9). (C) Virus titer was measured by plaque assay. Data represents values from three independent experiments (n=9), One-way ANOVA test followed by Dunnett test.

D. MEFs were transfected with siNT/siATG7 (50 nM) for 48 h, bar-graph shows relative Atg7 RNA levels determined by qRT-PCR.

E, F. MEFs were transfected with siNT/ATG7 for 48 h, infected with JEV (MOI 1), and treated with 10 µM MTP at 1 hpi. JEV RNA levels were estimated at 24 hpi through qRT-PCR. Bar-graph shows relative viral RNA levels after normalization to siNT/DMSO control, data is plotted from two independent experiments (n=6). (F) Virus titer was determined by plaque assay (n=6). Statistical significance was calculated by unpaired Student t-test.

Data information: All data are expressed as means ± SD, **, P<0.01; ***, P<0.001; **** P < 0.0001, ns; non-significant.

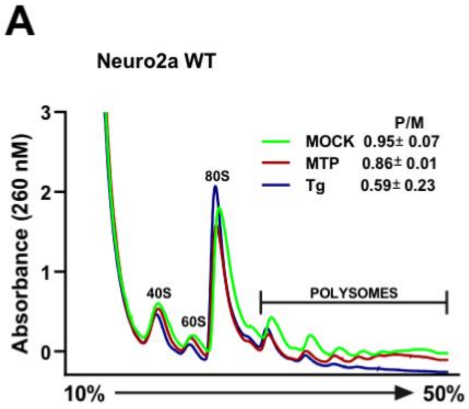

Appendix Figure S12: Effect of MTP treatment on global translation. Neuro2a WT (A) and ATG7 KO (B) cells were DMSO/MTP (10 µM)/Tg (1 µM) treated for 6 h. Global polysome profile analysis of cell lysates were performed by the density gradient fractionation system. Polysome-to-monosome (P/M) ratios from two independent experiments, means ± SD.

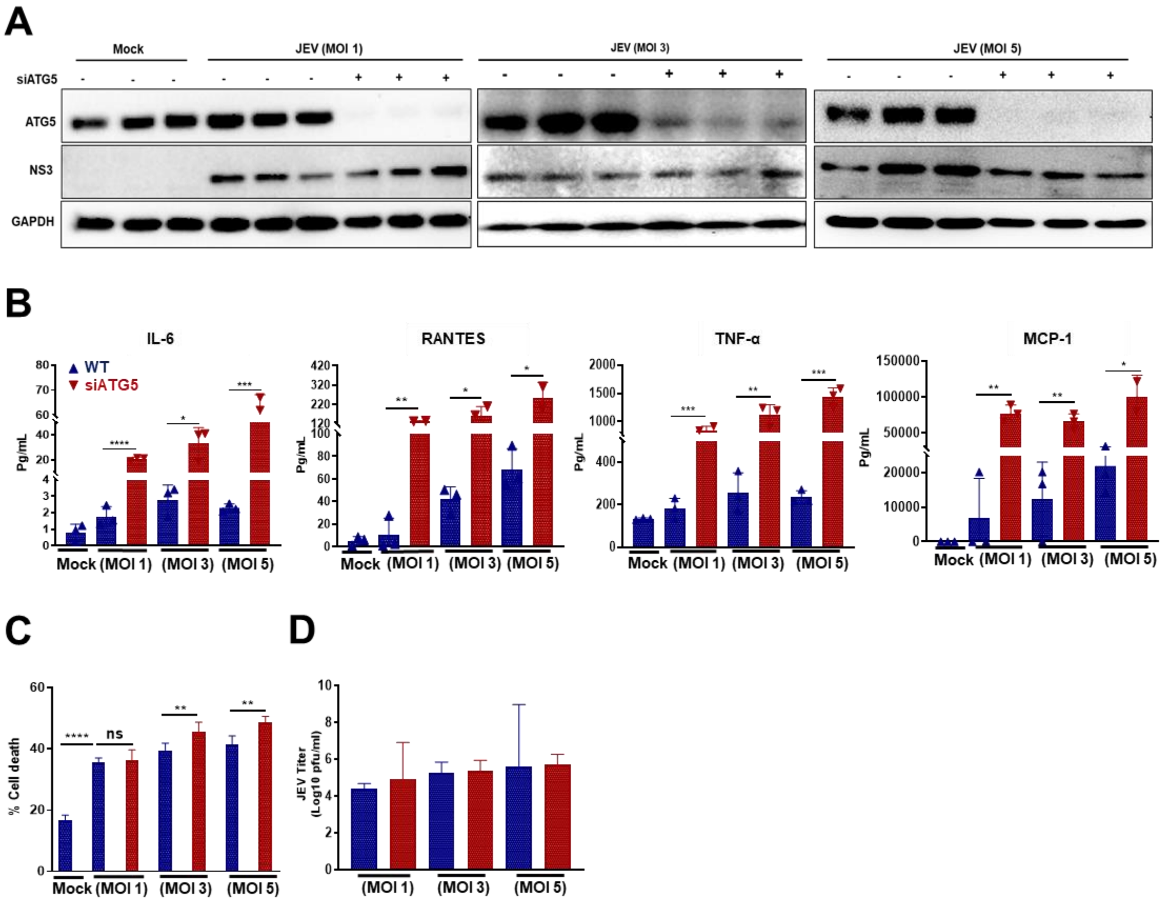

Appendix Figure S13. Autophagy restricts JEV-induced inflammatory response in microglial cells

N9 cells were transfected with siNT/ATG5 for 48 h, followed by mock/JEV infection (1/ 3/ 5 MOI) for 24 h.

A. Western blotting was performed using ATG5, NS3 (infection control), and GAPDH (internal control) antibodies (n=3).

B. Quantification of cytokine levels from the culture supernatant (n=3).

C. JEV-induced cell death is measured through LDH release assay (n=3).

D. JEV titers were quantified by plaque assay (n=3).

Data information: Statistical significance was determined by unpaired Student t-test, *, P<0.05; **, P<0.01; ***, P<0.001; **** P < 0.0001, ns; non-significant.

Appendix Table S1: Panel of FDA-approved drugs used in this study

S.No.	FDA-approved Drug	References (PMID)
1	ABT-737 (BH3 mimetics)	21460857
2	Amiodarone hydrochloride	18391949; 18024584
3	Bromhexine	26503418
4	Carbamazepine	18391949; 25535254
5	Clomiphene citrate	26503418
6	Clonidine hydrochloride	18391949
7	Desipramine	26503418; 24991837
8	Diperodon	26503418
9	Diphenidol	26503418
10	Flubendazole	26503418
11	Fluoxetine	26503418; 23364696
12	Fluspirilene	18024584
13	Fluvoxamine	26503418
14	Hexachlorophene	26503418
15	Lithium chloride	16186256
16	L-NAME	21726807
17	Loperamide hydrochloride	18391949
18	Maprotiline	26503418
19	Melengestrol	26503418
20	Metformin	21258367
21	Memantine	26503418
22	Methotrimeprazine	26503418
23	Minoxidil	18391949
24	Nicardipine	18024584
25	Niguldipine	18024584

26	Nimodipine	18391949
27	Nitrendipine	18391949
28	Nilvadipine	26503418
29	Nortriptyline	26503418
30	Oxiconazole	26503418
31	Pimozide	18024584
32	Proadifen	26503418
33	Resveratrol	22465415
34	Rilmenidine	20190273; 18391949
35	Sertraline	26503418
36	Sodium Valproate	18391949; 25535254
37	Statins-Pitavastatin	24433351
38	Ticlopidine	26503418
39	Trifluoperazine	18024584
40	Vitamin D	21454634; 22589721
41	Verapamil hydrochloride	18391949
42	Zotepine	26503418

Appendix Table S2: Percentage distribution of viral RNA and GAPDH mRNA in monosome vs polysome fractions at 6 hpi, mean ± S.D. n=2 biological replicates

**Neuro2a**					
		**WT**		**ATG7 KO**	
		**80S**	**POLYSOMES**	**80S**	**POLYSOMES**
**JEV**	**JEV**	21.12 ± 2.77	78.87 ± 2.77	18.13 ± 3.03	81.86 ± 3.03
	**JEV+MTP**	28.20 ± 1.95	71.79 ± 1.95	17.49 ± 10.40	82.50 ± 10.40
**GAPDH**	**MOCK**	3.22 ± 1.84	96.77 ± 1.84	4.09 ± 0.008	95.90 ± 0.008
	**JEV**	4.79 ± 1.89	95.20 ± 1.89	2.99 ± 0.76	97.00 ± 0.76
	**JEV+MTP**	10.32 ± 3.31	89.67 ± 3.31	4.24 ± 1.32	95.75 ± 1.32
**MEFs**					
		**WT**		**ATG5 KO**	
		**80S**	**POLYSOMES**	**80S**	**POLYSOMES**
**JEV**	**JEV**	3.53 ± 2.55	96.46 ± 2.55	7.52 ± 4.50	92.47 ± 4.50
	**JEV+MTP**	6.14 ± 6.37	93.85 ± 6.37	5.04 ± 0.93	94.95 ± 0.93
**GAPDH**	**MOCK**	1.48 ± 0.23	98.51 ± 0.23	1.94 ± 0.82	98.05 ± 0.82
	**JEV**	2.30 ± 0.78	97.69 ± 0.78	3.20 ± 2.02	96.79 ± 2.02
	**JEV+MTP**	2.79 ± 2.28	97.20 ± 2.28	2.65 ± 2.55	97.34 ± 2.55

Appendix Table S3: Chemical reagents used in this study

S. No.	Name of Reagent	Catalogue No.
1.	2-Mercaptoethanol	Sigma (M6250-250ML)
2.	ABT-737 (BH3 mimetic)	Sigma (197333-10MG)
3.	Agarose Type VII	Sigma (A4018)
4.	Amiodarone hydrochloride	Sigma (PHR1164-1G)
5.	Bafilomycin A1	Sigma (B1793)
6.	BCA assay kit	G-Biosciences (786-570)
7.	Bromhexine	Sigma (PHR1831-200MG)
8.	Carbamazepine	Sigma (PHR1067-1G)
9.	CellTiter-Glo® CellTiter-Glo® Luminescent Cell Viability Assay	Promega (G7572)
10.	Clomiphene citrate	Abcam (ab141183)
11.	Clonidine hydrochloride	Sigma (C7897-100MG)
12.	CM-H2DCFDA (General Oxidative Stress Indicator)	Invitrogen (C6827)
13.	Cycloheximide	Sugma (C7698-1G)
14.	CyQUANT™ LDH Cytotoxicity Assay	Invitrogen (C20301)
15.	Deoxyribonuclease I (DNase I)	SRL (61824)
16.	Desipramine	Sigma (D3900-1G)
17.	Dimethyl fumarate (DMF)	Sigma (242926-25G)
18.	Dimethyl sulfoxide (DMSO)	Sigma (276855-250ML)
19.	Diperodon hydrochloride	Sigma (D8536-5G)
20.	Diphenidol hydrochloride	Sigma (SML2169-100MG)
21.	DL-Dithiothreitol (DTT)	SRL (3483-12-3)
22.	EDTA	Sigma (E9884-500G)
23.	Evans blue	Sigma (E2129-10G)

24.	Flubendazole	Abcam (ab143260)
25.	Fluoxetine hydrochloride	Sigma (F132-10MG)
26.	Fluspirilene	Sigma (F100-10MG)
27.	Fluvoxamine maleate	Abcam (ab141082)
28.	Fura-2, AM	Invitrogen (F1225)
29.	Glucose	Sigma (G8270-100G)
30.	GUIDE-IT IVT RNA clean up kit	Takara (632638)
31.	Haloperidol	Sigma (H1512-5G)
32.	HBSS	Gibco (14175095)
33.	Heparin	G-Biosciences (RC1102)
34.	HEPES sodium salt	Sigma (H3784-500G)
35.	Hexachlorophene pestanal	Sigma (45526-250MG)
36.	ImProm-II™ Reverse Transcription System	Promega (A3800)
37.	Lithium chloride	Abcam (ab120853)
38.	L-NAME	Abcam (ab120136)
39.	Loperamide hydrochloride	Cayman Chemical (14875)
40.	LPS	Sigma (L2630)
41.	LysoSensor™ Yellow/Blue DND-160	Invitrogen (L7545)
42.	LysoTracker™ Red DND-99	Invitrogen (L7528)
43.	Magnesium acetate tetrahydrate	Sigma (M2545-250G)
44.	Maprotiline (hydrochloride)	Cayman Chemical (15892)
45.	Melengestrol acetate	Sigma (33998-100MG-R)
46.	Memantine hydrochloride	Sigma (M9292-25MG)
47.	Metformin	Abcam (ab120847)
48.	Methotrimeprazine	MedChem Express (HY-B1693)
49.	Minoxidil	Sigma (M4145-25MG)

50.	MTT 3-(4,5-Dimethylthiazol-2-yl)-2,5-Diphenyltetrazolium Bromide	VWR life science, (0793-1G)
51.	N-Acetyl-L-cysteine (NAC)	Sigma (A7250-10G)
52.	Nicardipine hydrochloride	Sigma (N7510-1G)
53.	Niguldipine (hydrochloride)	Cayman Chemical (19534)
54.	Nilvadipine	Sigma (SML0945-10MG)
55.	Nimodipine	Sigma (N149-100MG)
56.	Nitrendipine	Sigma (N144-25MG)
57.	Nortriptyline (hydrochloride)	Cayman Chemical (15904)
58.	Oxiconazole	Sigma (SML1474-10MG)
59.	Phenylmethylsulfonyl fluoride (PMSF)	Sigma (329-98-6)
60.	Pimozide	Abcam (ab142135)
61.	Poly (ethylene glycol) (PEG 400)	Sigma (202398-500G)
62.	Potassium acetate	Himedia (MB042-500G)
63.	Premix Ex Taq™ (Probe qPCR)	Takara (RR390A)
64.	Proadifen	Sigma (P1061-100MG)
65.	ProLong™ Gold Antifade Mountant with DAPI	Invitrogen (P36935)
66.	Protease inhibitor cocktail (PI)	Sigma (P8340)
67.	Puromycin	InvivoGen (ant-pr-1)
68.	PVDF membrane	Merck Millipore (IPVH00010)
69.	Random hexamer	Sigma (H0268)
70.	RBC lysis buffer	GCC Biotech (19114B1076)
71.	Resveratrol	Abcam (ab120726)
72.	RiboprobeR combination systems kit	Promega (P1460)
73.	Rilmenidine hemifumarate salt	Sigma (R134-5MG)
74.	SDS	Sigma (L3771-500G)

75.	Sodium pyruvate	Himedia (TCL015)
76.	Sodium Valproate	Abcam (ab120745)
77.	Statins-Pitavastatin	Cayman Chemical (15414)
78.	Sucrose	Sigma (S1888-1KG)
79.	SYBR® Premix Ex Taq™	Takara (RR420A)
80.	Thapsigargin	Sigma (T9033)
81.	Ticlopidine	Sigma (T6654-1G)
82.	Torin1	Tocris Bioscience (4247)
83.	Trifluoperazine Dihydrochloride	Cayman Chemical (15068)
84.	Triton™ X-100	Sigma (T9284-500ML)
85.	Trizol reagent (RNAiso Plus)	Takara (9109)
86.	Tween 20	G-Biosciences (RC1227)
87.	Verapamil hydrochloride	Abcam (ab120140)
88.	Vitamin D	Cayman Chemical (11791)
89.	Zotepine	Sigma (Z0877-10MG)
	**Media & other additives**	**Catalogue No.**
90.	2XMEM	Himedia (AL178A-500ML)
91.	B-27	Gibco (17504044)
92.	DMEM	Himedia (AL007A-500ML)
93.	FBS	Himedia, (RM10432-500ML)
94.	Geneticin™ Selective Antibiotic (G418 Sulfate)	Gibico (10131035)
95.	L-15	Himedia (AL011S-500ML)
96.	L929 conditioned media	Culture supernatant of L929 fibroblast cells (After 6-8 days starvation of L929 cells)
97.	L-Glutamine	Himedia (TCL012)
98.	MEM	Hyclone (SH3024401-500ML)

99.	Neurobasal	Gibco (21103049)
100.	Penicillin-Streptomycin	Himedia (A007-100ML)
101.	RPMI-1640	Himedia (AL028-500ML)
102.	Trypsin - EDTA Solution	Himedia (TCL007)
	**Antibodies**	**Catalogue No.**
103.	Alexa fluor™ 488 chicken anti-mouse IgG (H+L)	Invitrogen (A-21200, dilution 1:500)
104.	Alexa fluor™ 568 donkey anti-mouse IgG (H+L)	Invitrogen (A-10037, dilution 1:500)
105.	ATG5	CST (12994S, dilution 1:1000)
106.	ATG7	CST (2631S, dilution 1:1000)
107.	GAPDH	GeneTex (GTX100118, dilution 1:10000)
108.	GFAP	Millipore (MAB3402, dilution 1:500)
109.	IBA1	Millipore (MABN92, dilution 1:500)
110.	JEV-Envelope	Abcam (ab41671, dilution 1:50)
111.	JEV-NS1	Abcam (ab41651, dilution 1:50)
112.	JEV-NS5	GeneTex (GTX131359, dilution 1:2000)
113.	LC3B	Abcam (ab51520, dilution 1:2000)
114.	mTOR	CST (2983S, dilution 1:1000)
115.	NeuN	Millipore (ABN78, dilution 1:500)
116.	NLRP3	CST (15101S, dilution 1:1000)
117.	p-4E-BP1 (Thr37/46)	CST (2855S, dilution 1:1000)
118.	p70S6Kinase	CST (9202S, dilution 1:1000)
119.	Peroxidase AffiniPure Donkey Anti-Mouse IgG (H+L)	Jackson ImmunoResearch (715-035-150, dilution 1:5000)
120.	Peroxidase AffiniPure Donkey Anti-Rabbit IgG (H+L)	Jackson ImmunoResearch (711-035-152, dilution 1:10000)
121.	p-mTOR (Ser2448)	CST (5536S, dilution 1:1000)

122.	p-p70S6Kinase (Thr389)	CST (97596S, dilution 1:1000)
123.	SQSTM1/ p62	Abcam (ab56416, dilution 1:1000)
	**Plasmids**	**Catalogue No.**
124.	gag/pol	Addgene (14887)
125.	pCI-VSVG	Addgene (1733)
126.	pEGFP-LC3	Addgene (21073)
127.	pEGFP-N1-TFEB	Addgene (38119)
128.	pMRX-IP-GFP-LC3-RFP-LC3ΔG	Addgene (84572)
129.	pSpCas9(BB)-2A-GFP (PX458)	Addgene (48138)
130.	pSpCas9(BB)-2A-Puro(PX459) V2.0	Addgene (62988)
	**siRNA/Transfection reagents**	**Catalogue No.**
131.	DharmaFECT 2	Dharmacon (T-2002-02)
132.	Lipofectamine™ RNAimax	Invitrogen (13778030)
133.	Lipofectamine™ 2000	Invitrogen (11668019)
134.	Lipofectamine™ 3000	Invitrogen (L3000015)
135.	ON-TARGETplus Non-targeting (NT)	Dharmacon (D-001810-10-20)
136.	ON-TARGETplus Mouse siAtg5	Dharmacon (L-064838-00-0005)
137.	ON-TARGETplus Mouse siAtg7	Dharmacon (L-049953-00-0005)
	**Cytokines**	**Catalogue No.**
138.	Mouse IL-1α Flex Set	BD Bioscience (560157)
139.	Mouse IL-1β Flex Set	BD Bioscience (560232)
140.	Mouse IL-6 Flex Set	BD Bioscience (558301)
141.	LEGENDPLEX MU Anti-Virus Response Panel (13-plex)	Biolegend (740622)
142.	Mouse MCP-1 Flex Set	BD Bioscience (558342)
143.	Mouse/Rat Soluble Protein Master Buffer Kit	BD Bioscience (558266)

144.	Mouse RANTES Flex Set	BD Bioscience (558345)
145.	Mouse TNF Flex Set	BD Bioscience (558299)

Appendix Table S4: Primers used in this study

S.No.	Gene name	Forward (5'-3') sequence	Reverse (5'-3') sequence
1.	ABHD4	GGCACAGTTTGGGAGGATTCC	ACTAGGGTCAGTTGGTCGTAG
2.	AKT2	ATGAACGACGTAGCCATTGTG	TTGTAGCCAATAAAGGTGCCAT
3.	AMPK/PRKAA1	GTCAAAGCCGACCCAATGATA	CGTACACGCAAATAATAGGGGTT
4.	ASC	CTTGTCAGGGGATGAACTCAAAA	GCCATACGACTCCAGATAGTAGC
5.	ATF4	CTCTTGACCACGTTGGATGAC	CAACTTCACTGCCTAGCTCTAAA
6.	ATF6 A	CGGTCCACAGACTCGTGTTC	GCTGTCGCCATATAAGGAAAGG
7.	ATG12	TCCCCGGAACGAGGAACTC	TTCGCTCCACAGCCCATTTC
8.	ATG13	CCAGGCTCGACTTGGAGAAAA	AGATTTCCACACACATAGATCGC
9.	ATG14	GAGGGCCTTTACGTGGCTG	AATAGACGAAATCACCGCTCTG
10.	ATG16L1	CAGAGCAGCTACTAAGCGACT	AAAAGGGGAGATTCGGACAGA
11.	ATG3	ACACGGTGAAGGGAAAGGC	TGGTGGACTAAGTGATCTCCAG
12.	ATG4A	GCTGGTATGGATTCTGGGGAA	TGGGTTGTTCTTTTTGTCTCTCC
13.	ATG4B	TATGATACTCTCCGGTTTGCTGA	GTTCCCCCAATAGCTGGAAAG
14.	ATG5	TGTGCTTCGAGATGTGTGGTT	GTCAAATAGCTGACTCTTGGCAA
15.	ATG7	GTTCGCCCCCTTTAATAGTGC	TGAACTCCAACGTCAAGCGG
16.	ATG9A	CAGTTTGACACTGAATACCAGCG	AATGTGGTGCCAAGGTGATTT
17.	BCL2	ATGCCTTTGTGGAACTATATGGC	GGTATGCACCCAGAGTGATGC
18.	BECN1	ATGGAGGGGTCTAAGGCGTC	TCCTCTCCTGAGTTAGCCTCT
19.	CASP1	ACAAGGCACGGGACCTATG	TCCCAGTCAGTCCTGGAAATG
20.	CASP11	ACAAACACCCTGACAAACCAC	CACTGCGTTCAGCATTGTTAAA
21.	CGAS	GAGGCGCGGAAAGTCGTAA	TTGTCCGGTTCCTTCCTGGA
22.	CH25H	TGCTACAACGGTTCGGAGC	AGAAGCCCACGTAAGTGATGAT
23.	CHOP/DDIT3	ACCTTCACTACTCTTGACCCTG	GATGTGCGTGTGACCTCTGT
24.	CYP51A1	GACAGGAGGCAACTTGCTTTC	GTGGACTTTTCGCTCCAGC
25.	DDX58	AAGAGCCAGAGTGTCAGAATCT	AGCTCCAGTTGGTAATTTCTTGG
26.	DHCR7	AGGCTGGATCTCAAGGACAAT	GCCAGACTAGCATGGCCTG
27.	DNAJC3	GGCGCTGAGTGTGGAGTAAAT	GCGTGAAACTGTGATAAGGCG
28.	DRP1	CAGGAATTGTTACGGTTCCCTAA	CCTGAATTAACTTGTCCCGTGA
29.	ECH1	GCTACCGCGATGACAGTTTC	TCAGAGATCGAAGGCTGATGTT
30.	EIF2AK2/PKR	ATGCACGGAGTAGCCATTACG	TGACAATCCACCTTGTTTTCGT
31.	FADS1	AGCACATGCCATACAACCATC	TTTCCGCTGAACCACAAAATAGA
32.	FASN F	GGAGGTGGTGATAGCCGGTAT	TGGGTAATCCATAGAGCCCAG
33.	GADD34/PPP1R15A	GCCTGCAAGGGGCTGATAAG	TTTGTATCCCGGAGCTATGGA
34.	GAPDH	CGTCCCGTAGACAAAATGGT	TTGATGGCAACAATCTCCAC
35.	GM2A	CGCCTTTCCCAACTTGGTG	TGACGACTACATCTCCAGGAAC
36.	GRP78/HSPA5	GCATCACGCCGTCGTATGT	ATTCCAAGTGCGTCCGATGAG
37.	GSDMD	CCATCGGCCTTTGAGAAAGTG	ACACATGAATAACGGGGTTTCC
38.	HGAPDH	TGCACCACCAACTGCTTACG	GGCATGGACTGTGGTCATGAG
39.	HMGCR	AGCTTGCCCGAATTGTATGTG	TCTGTTGTGAACCATGTGACTTC
40.	IFIT1	CTGAGATGTCACTTCACATGGAA	GTGCATCCCCAATGGGTTCT

41.	IFNB1	CAGGTAGTAGGCGACACTGT	TCAATTGCCACAGGAGCTTC
42.	IFN-α	ATGGCTAGRCTC TGTGCTTTCCT	AGGGCTCTCCAGAYTTCTGCTCTG
43.	IFN-β	AAGAGTTACACTGCCTTTGCCATC	CACTGTCTGCTGGTGGAGTTCATC
44.	IFN-γ	GGCCATCAGCAACATAAGCGT	TGGGTTGTTGACCTCAAACTTGGC
45.	IL-6	CTGCAAGAGACTTCCATCCAG	AGTGGTATAGACAGGTCTGTTGG
46.	IRE1 ALPHA/*ERN1*	ACACCGACCACCGTATCTCA	CTCAGGATAATGGTAGCCATGTC
47.	IRF7	GAGACTGGCTATTGGGGGAG	GACCGAAATGCTTCCAGGG
48.	JEV	AGAGCACCAAGGGAATGAAATAGT<br>Taqman probe: CCACGCCACTCGACCCATAGACTG (5′ end FAM, 3′end TAMRA).	AATAAGTTGTAGTTGGGCACTCTG
49.	LAMP1	CAGCACTCTTTGAGGTGAAAAAC	ACGATCTGAGAACCATTCGCA
50.	LAMP2	TGTATTTGGCTAATGGCTCAGC	TATGGGCACAAGGAAGTTGTC
51.	LC3A	GACCGCTGTAAGGAGGTGC	CTTGACCAACTCGCTCATGTTA
52.	LC3B	TTATAGAGCGATACAAGGGGGAG	CGCCGTCTGATTATCTTGATGAG
53.	MCP-1/CCL2	CAAGAAGGAATGGGTCCAGA	GCTGAAGACCTTAGGGCAGA
54.	MDA5	AGATCAACACCTGTGGTAACACC	CTCTAGGGCCTCCACGAACA
55.	MLKL	AATTGTACTCTGGGAAATTGCCA	TCTCCAAGATTCCGTCCACAG
56.	MSMO1	AAACAAAAGTGTTGGCGTGTTC	AAGCATTCTTAAAGGGCTCCTG
57.	mTOR	ACCGGCACACATTTGAAGAAG	CTCGTTGAGGATCAGCAAGG
58.	Negative sense_probe	FAM-GCATTAGCCCCGACCAAGGCG-TAMRA	
59.	NRF2/NFE212	TAGATGACCATGAGTCGCTTGC	GCCAAACTTGCTCCATGTCC
60.	OAS1	GCCTGATCCCAGAATCTATGC	GAGCAACTCTAGGGCGTACTG
61.	PERK/E*IF2AK3*	GCACTTTAGATGGACGAATCGC	TGCTGAGGCTAGATGAAACCA
62.	PGAM5	ATCTGGAGAAGACGAGTTGACA	CCTGTTCCCGACCTAATGGT
63.	PI3KCA	CCACGACCATCTTCGGGTG	ACGGAGGCATTCTAAAGTCACTA
64.	PRKAA2	CAGGCCATAAAGTGGCAGTTA	AAAAGTCTGTCGGAGTGCTGA
65.	RANTES/CCL5	GCTGCTTTGCCTACCTCTCC	TCGAGTGACAAACACGACTGC
66.	RIG-I	ACAGATCCGAGACACTAAAGGG	AACAGCGCCTCTGATGGAAAG
67.	RIPK1	GAAGACAGACCTAGACAGCGG	CCAGTAGCTTCACCACTCGAC
68.	RIPK3	TCTGTCAAGTTATGGCCTACTGG	GGAACACGACTCCGAACCC
69.	SCD2	GCATTTGGGAGCCTTGTACG	AGCCGTGCCTTGTATGTTCTG
70.	SQLE	ATAAGAAATGCGGGGATGTCAC	ATATCCGAGAAGGCAGCGAAC
71.	SREBP2	GCAGCAACGGGACCATTCT	CCCCATGACTAAGTCCTTCAACT
72.	TLR-3	GTGAGATACAACGTAGCTGACTG	TCCTGCATCCAAGATAGCAAGT
73.	Neg qPCR	GGCCGTCATGGTGGCGAATAA	GTCAATGAGTGTTCCAAGTTCTCG
74.	NVnegVneg	<u>GGCCGTCATGGTGGCGAATAA</u>GAGC<br>TTGTTGGACGGTAGAG	Underlined sequence represents non-viral tag sequence.
75.	TNFR1sf1a	CCGGGAGAAGAGGGATAGCTT	TCGGACAGTCACTCACCAAGT
76.	TNF-α	CCCTCACACTCAGATCATCTTCT	GCTACGACGTGGGCTACAG
77.	ULK1	AAGTTCGAGTTCTCTCGCAAG	CGATGTTTTCGTGCTTTAGTTCC
78.	VPS34/PIK3C3	CCTGGACATCAACGTGCAG	TGTCTCTTGGTATAGCCCAGAAA

79.	XBP-1	AGCAGCAAGTGGTGGATTTG	GAGTTTTCTCCCGTAAAAGCTGA
80.	XBP-1 SPLICED	GACAGAGAGTCAAACTAACGTGG	GTCCAGCAGGCAAGAAGGT
