# Supplementary figures and images for: Methotrimeprazine is a neuroprotective antiviral in JEV infection via adaptive ER stress and autophagy
Source: EMBO Mol Med. 2024 Jan 2;16(1):185–217. doi: 10.1038/s44321-023-00014-w (PMC10897192; doi:10.1038/s44321-023-00014-w)

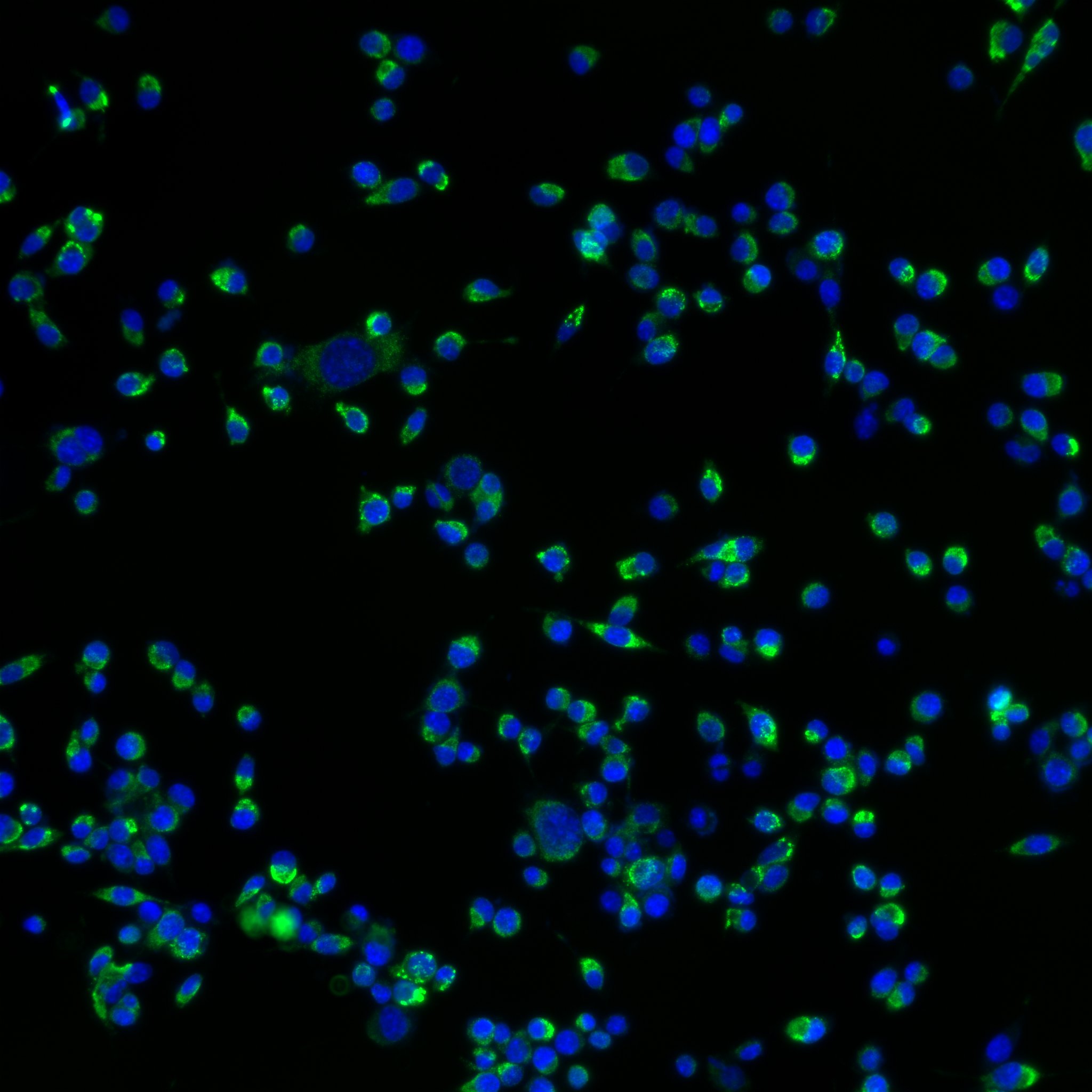

Supplement: Supplementary file 3 — Source Data Fig. 1 [file 44321_2023_14_MOESM3_ESM.zip › Figure 1/Fig 1G/DMSO.tif]

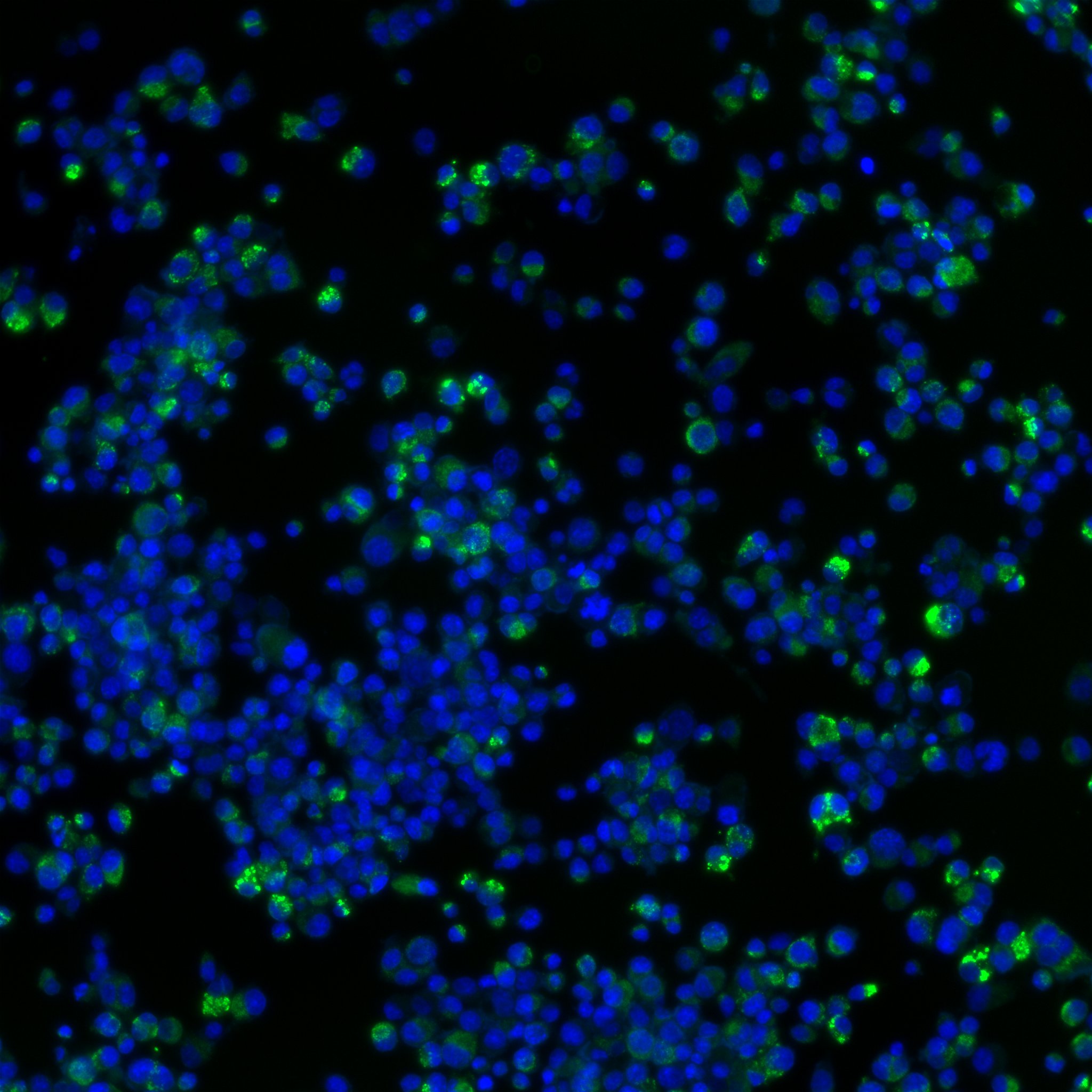

Supplement: Supplementary file 3 — Source Data Fig. 1 [file 44321_2023_14_MOESM3_ESM.zip › Figure 1/Fig 1G/Flubendazole.tif]

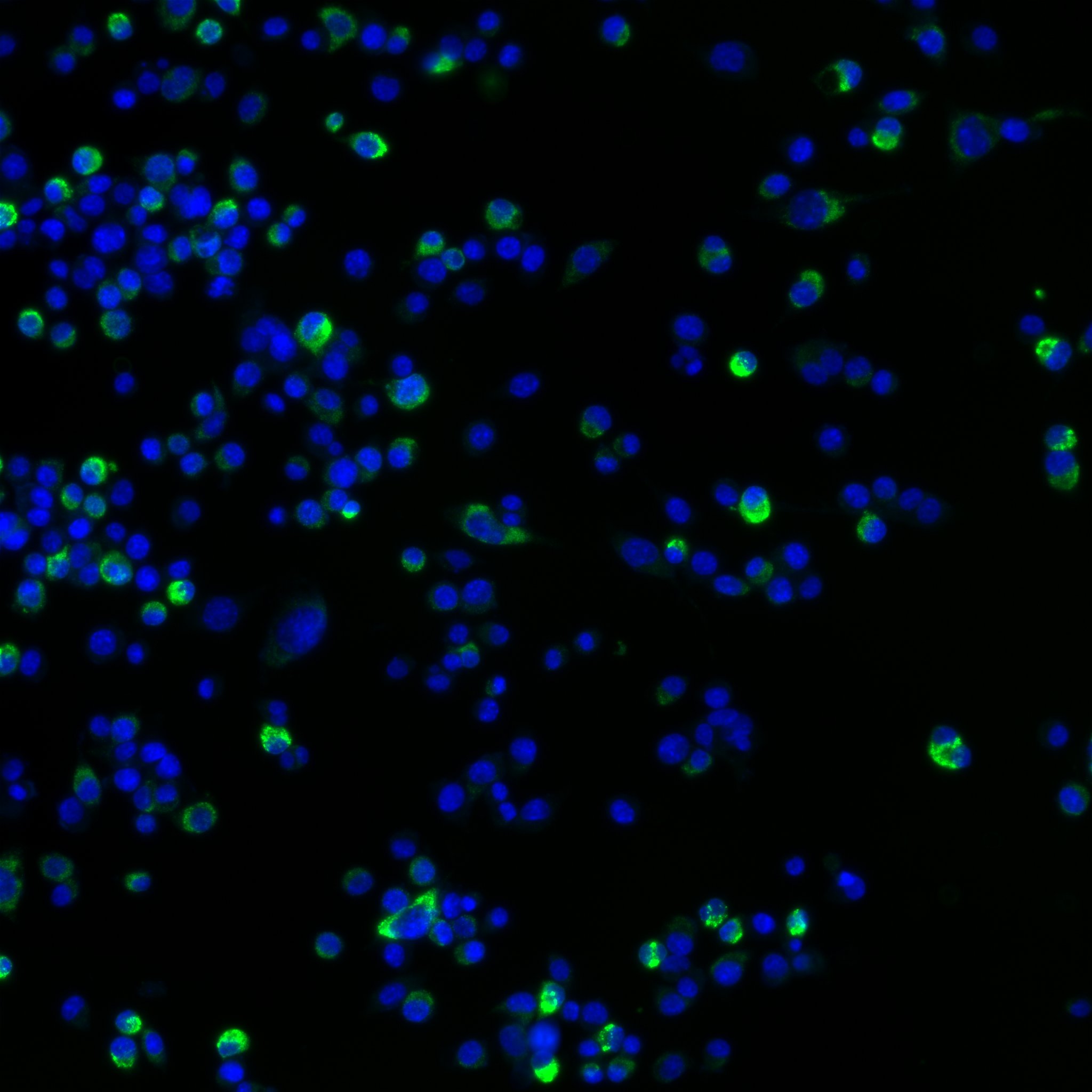

Supplement: Supplementary file 3 — Source Data Fig. 1 [file 44321_2023_14_MOESM3_ESM.zip › Figure 1/Fig 1G/Fluoxetine.tif]

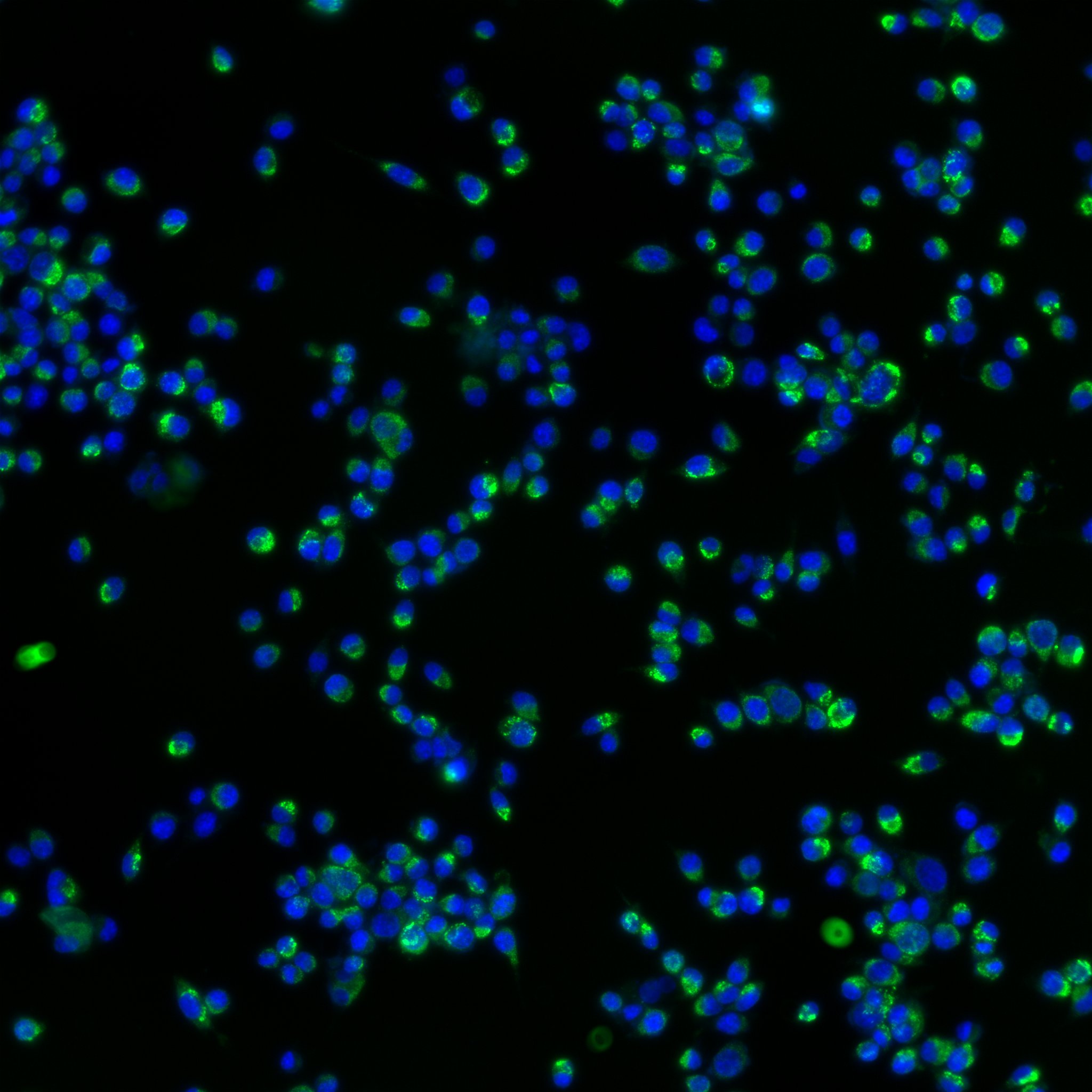

Supplement: Supplementary file 3 — Source Data Fig. 1 [file 44321_2023_14_MOESM3_ESM.zip › Figure 1/Fig 1G/Memantine.tif]

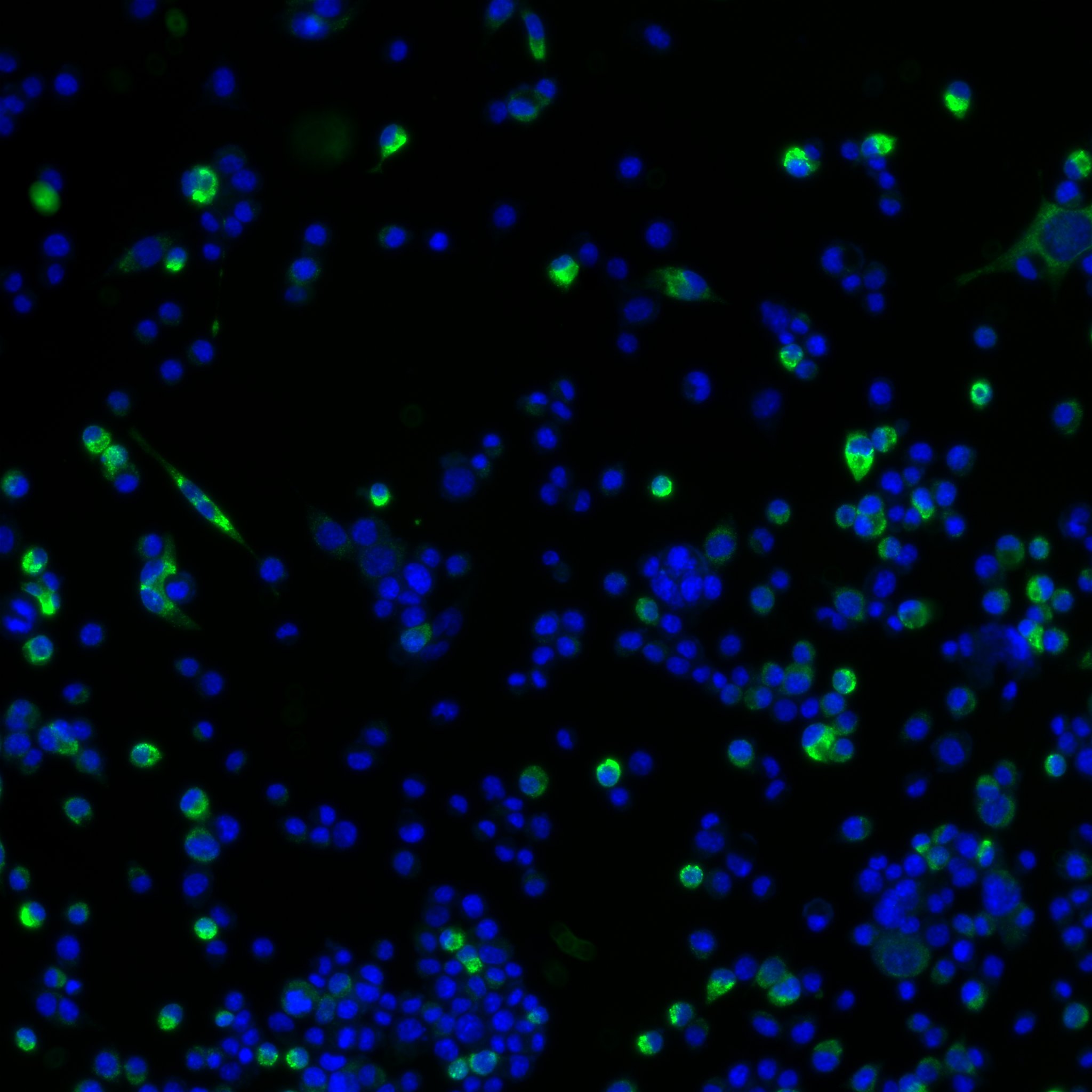

Supplement: Supplementary file 3 — Source Data Fig. 1 [file 44321_2023_14_MOESM3_ESM.zip › Figure 1/Fig 1G/MTP.tif]

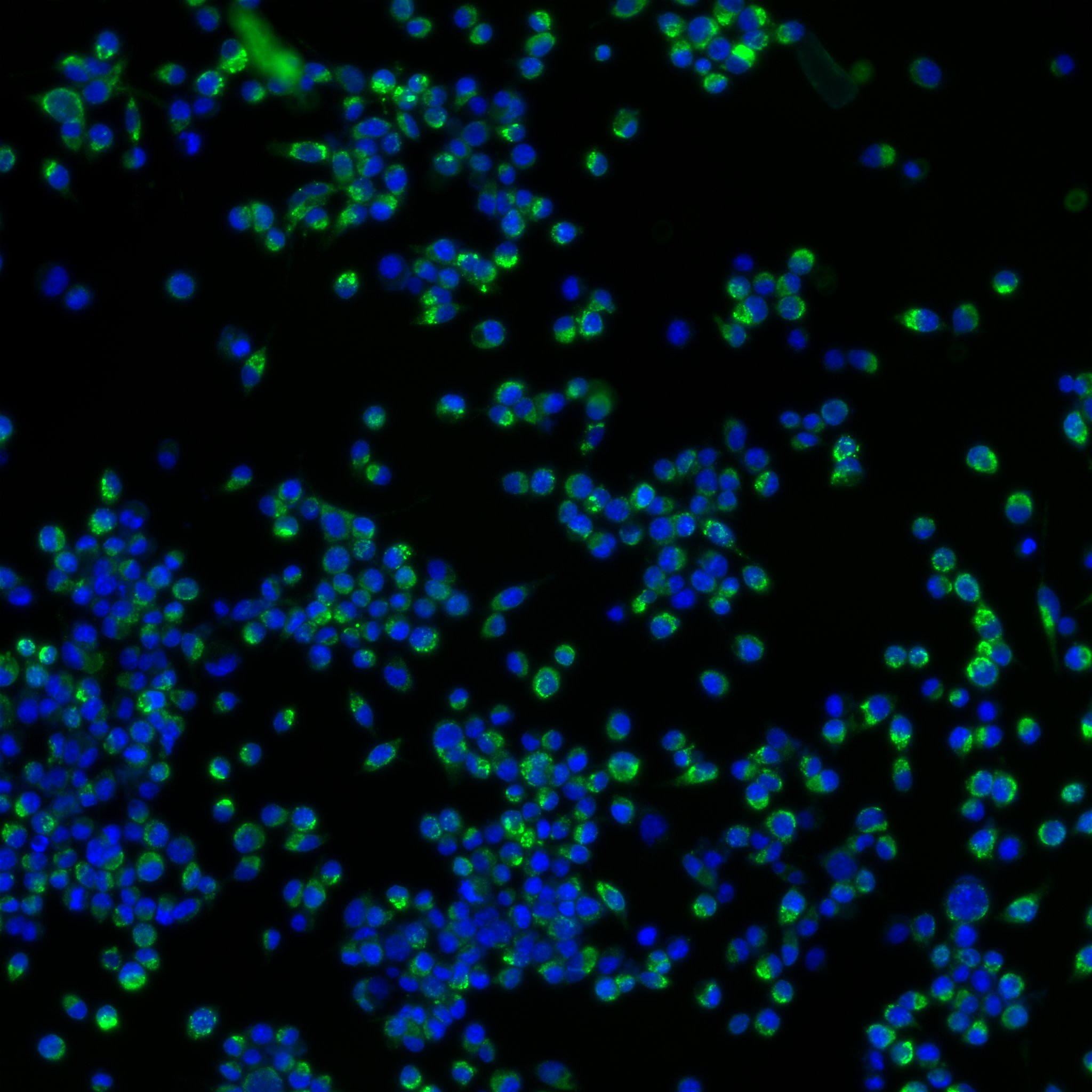

Supplement: Supplementary file 3 — Source Data Fig. 1 [file 44321_2023_14_MOESM3_ESM.zip › Figure 1/Fig 1G/Rilmenidine.tif]

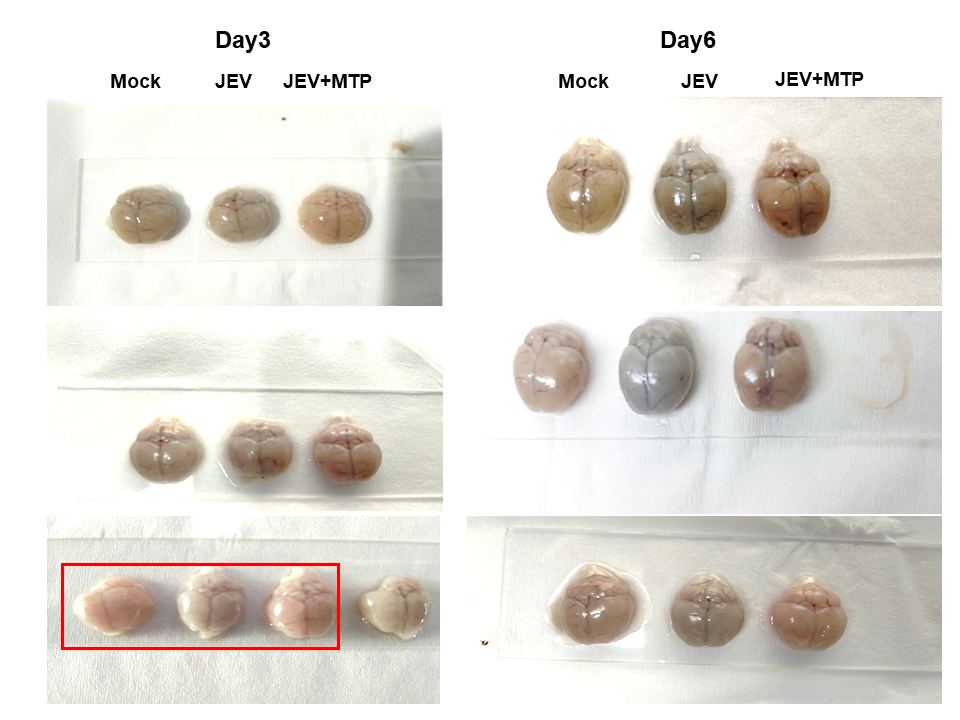

Supplement: Supplementary file 4 — Source Data Fig. 2 [file 44321_2023_14_MOESM4_ESM.zip › Figure 2/Fig 2D/Evans blue.tif]

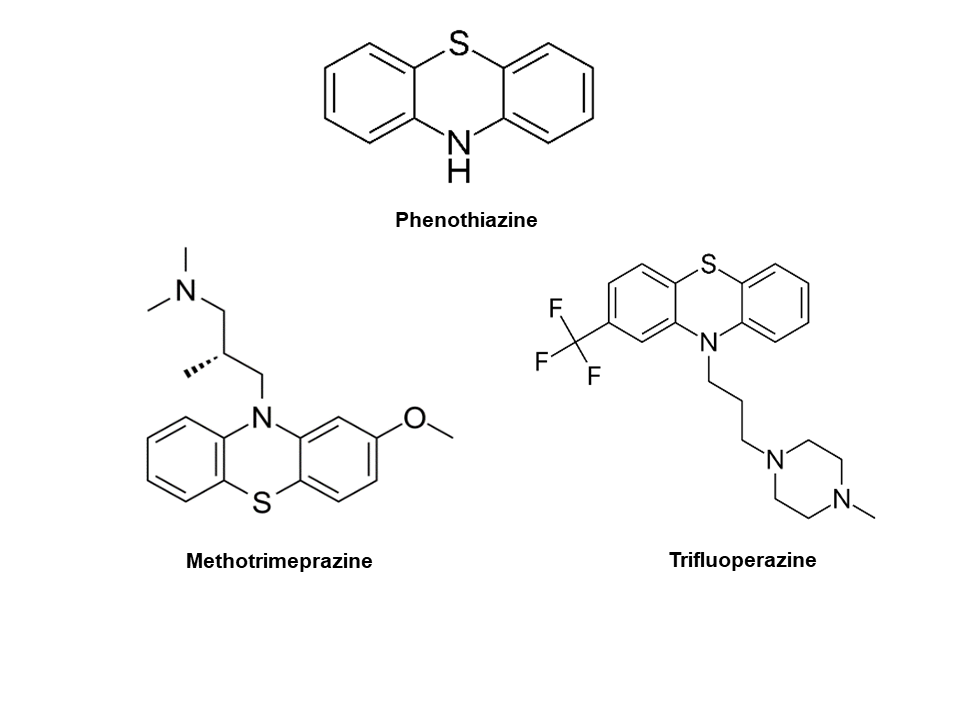

Supplement: Supplementary file 5 — Source Data Fig. 3 [file 44321_2023_14_MOESM5_ESM.zip › Figure 3/Fig 3A/Chemical structures.tif]

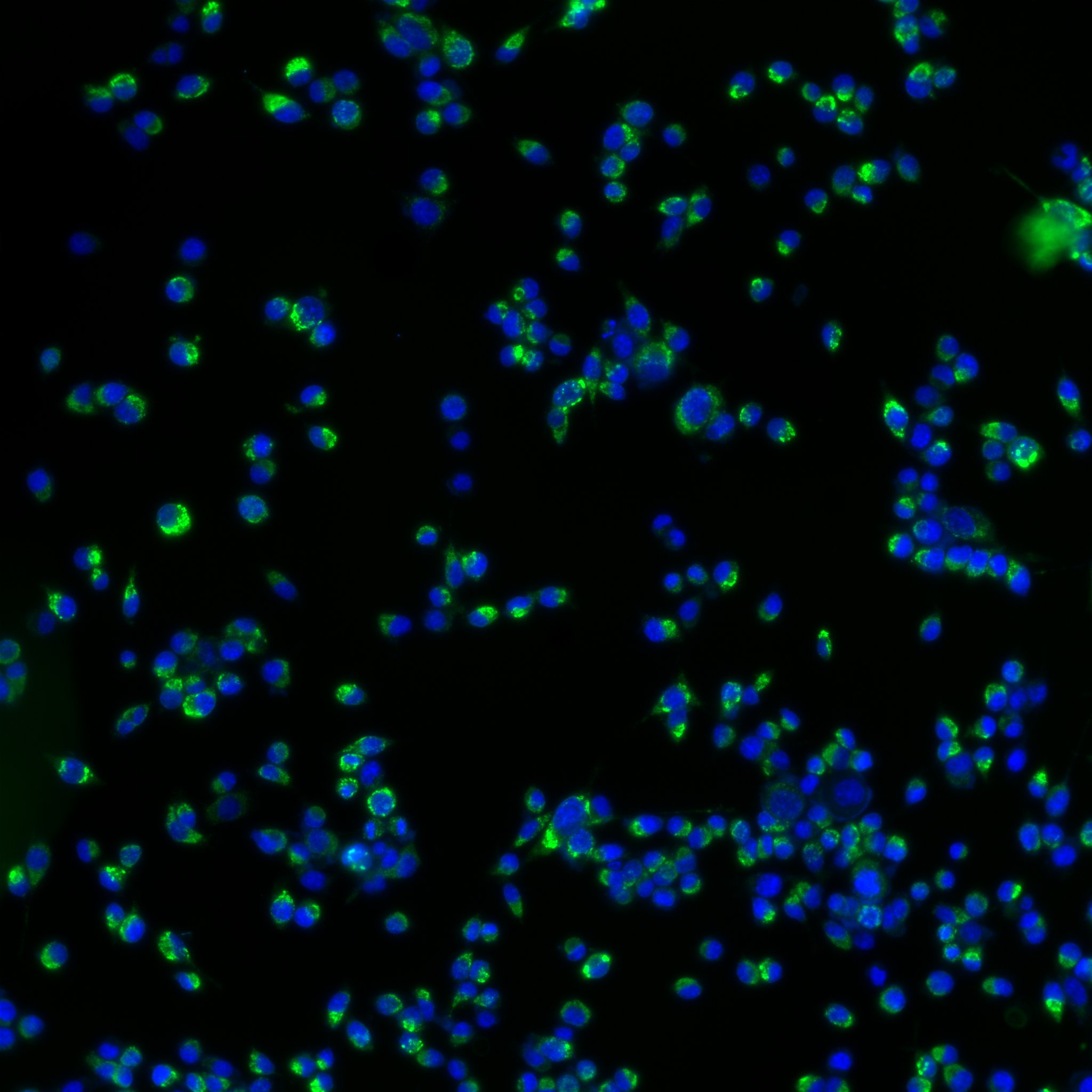

Supplement: Supplementary file 5 — Source Data Fig. 3 [file 44321_2023_14_MOESM5_ESM.zip › Figure 3/Fig 3H/DMSO.tif]

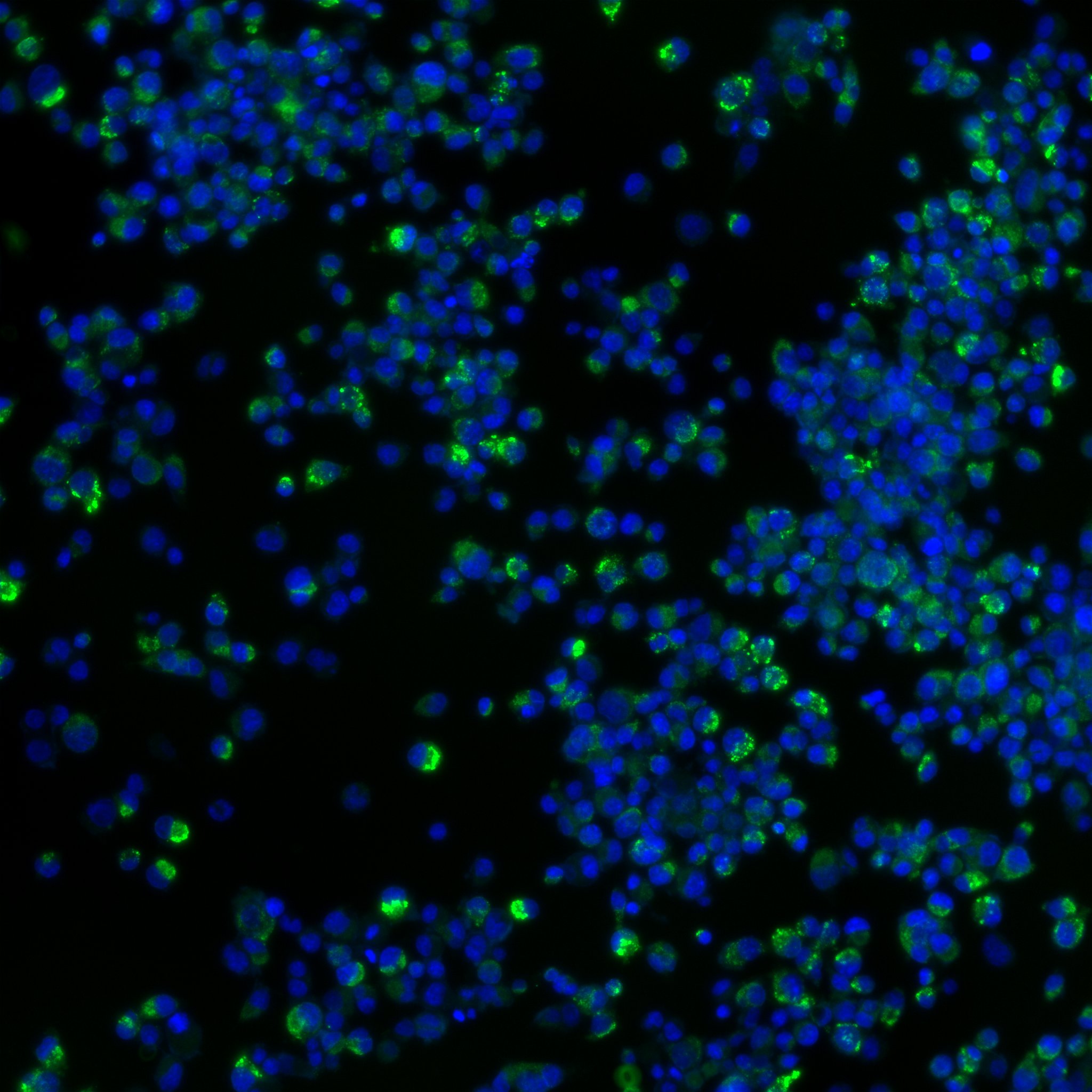

Supplement: Supplementary file 5 — Source Data Fig. 3 [file 44321_2023_14_MOESM5_ESM.zip › Figure 3/Fig 3H/TFP.tif]

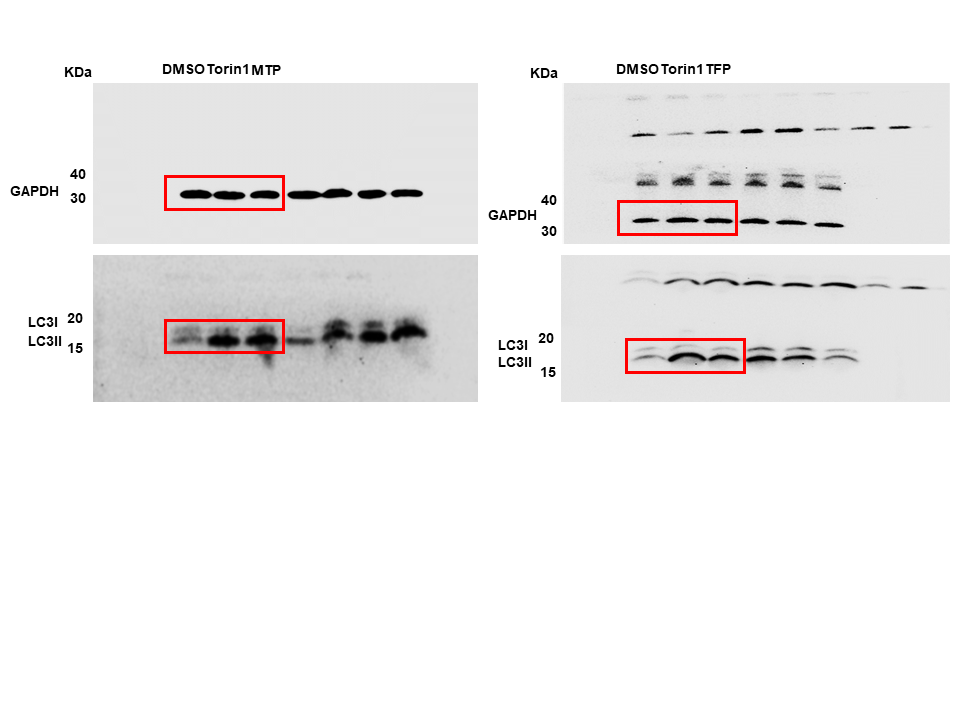

Supplement: Supplementary file 6 — Source Data Fig. 4 [file 44321_2023_14_MOESM6_ESM.zip › Figure 4/Fig 4A/Exp 1.tif]

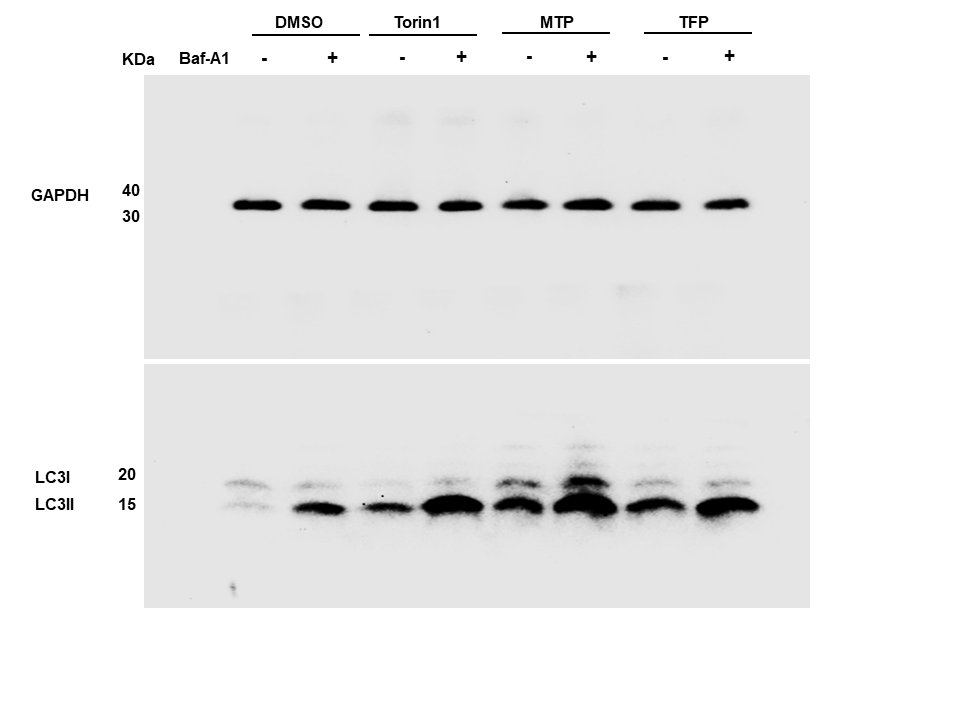

Supplement: Supplementary file 6 — Source Data Fig. 4 [file 44321_2023_14_MOESM6_ESM.zip › Figure 4/Fig 4A/Exp 2.tif]

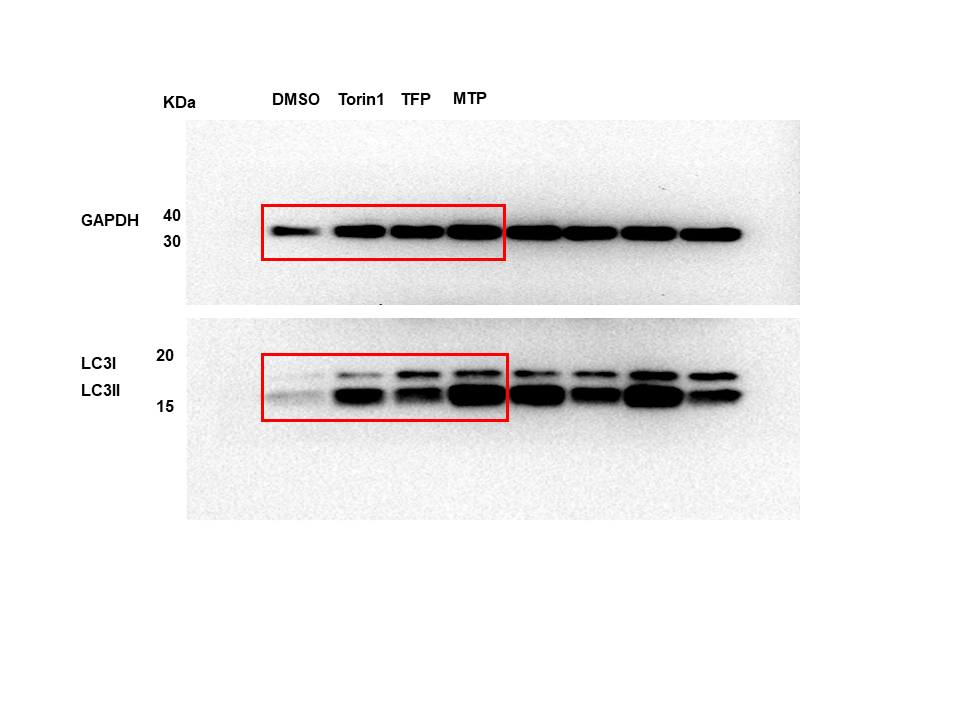

Supplement: Supplementary file 6 — Source Data Fig. 4 [file 44321_2023_14_MOESM6_ESM.zip › Figure 4/Fig 4A/Exp 3.tif]

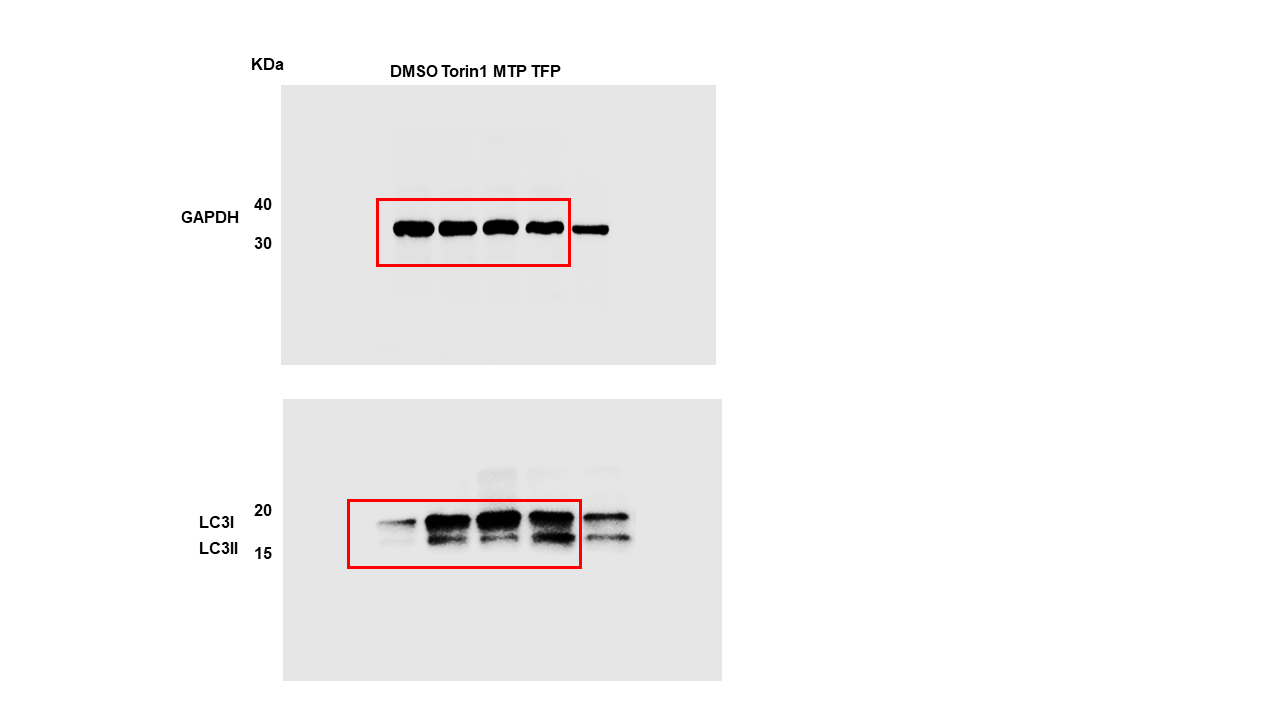

Supplement: Supplementary file 6 — Source Data Fig. 4 [file 44321_2023_14_MOESM6_ESM.zip › Figure 4/Fig 4C/Exp 1.tif]

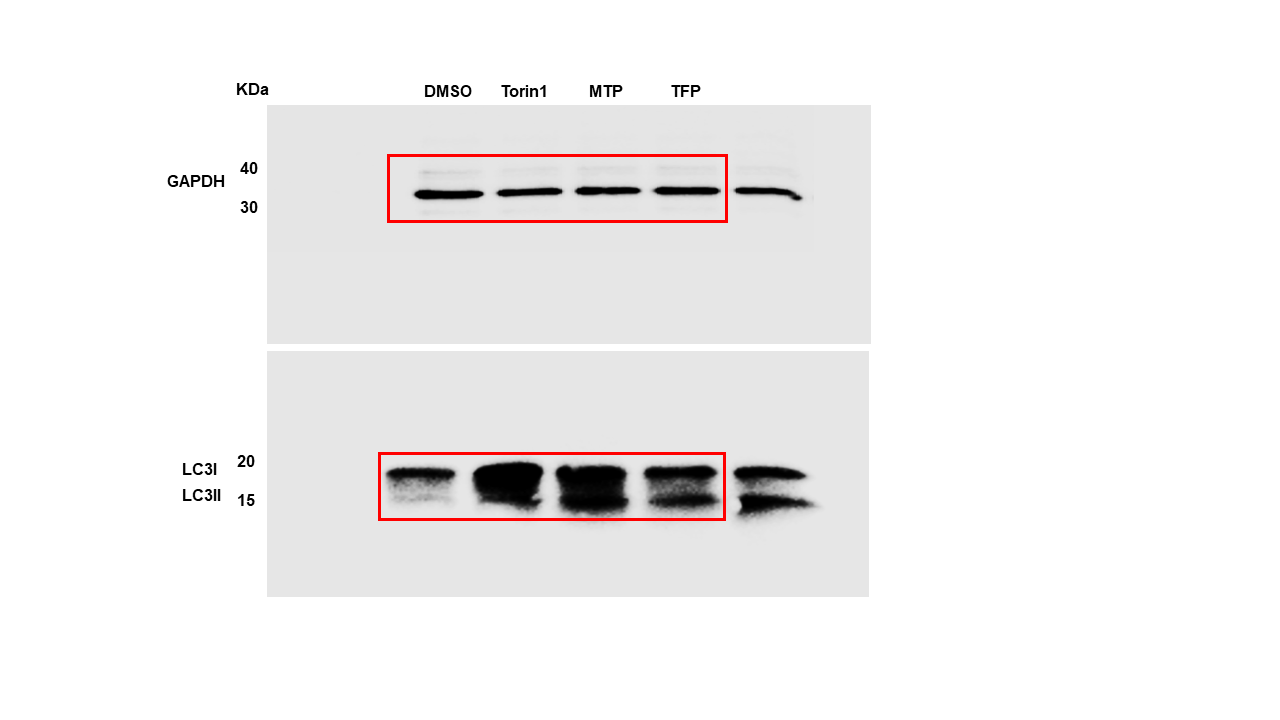

Supplement: Supplementary file 6 — Source Data Fig. 4 [file 44321_2023_14_MOESM6_ESM.zip › Figure 4/Fig 4C/Exp 2.tif]

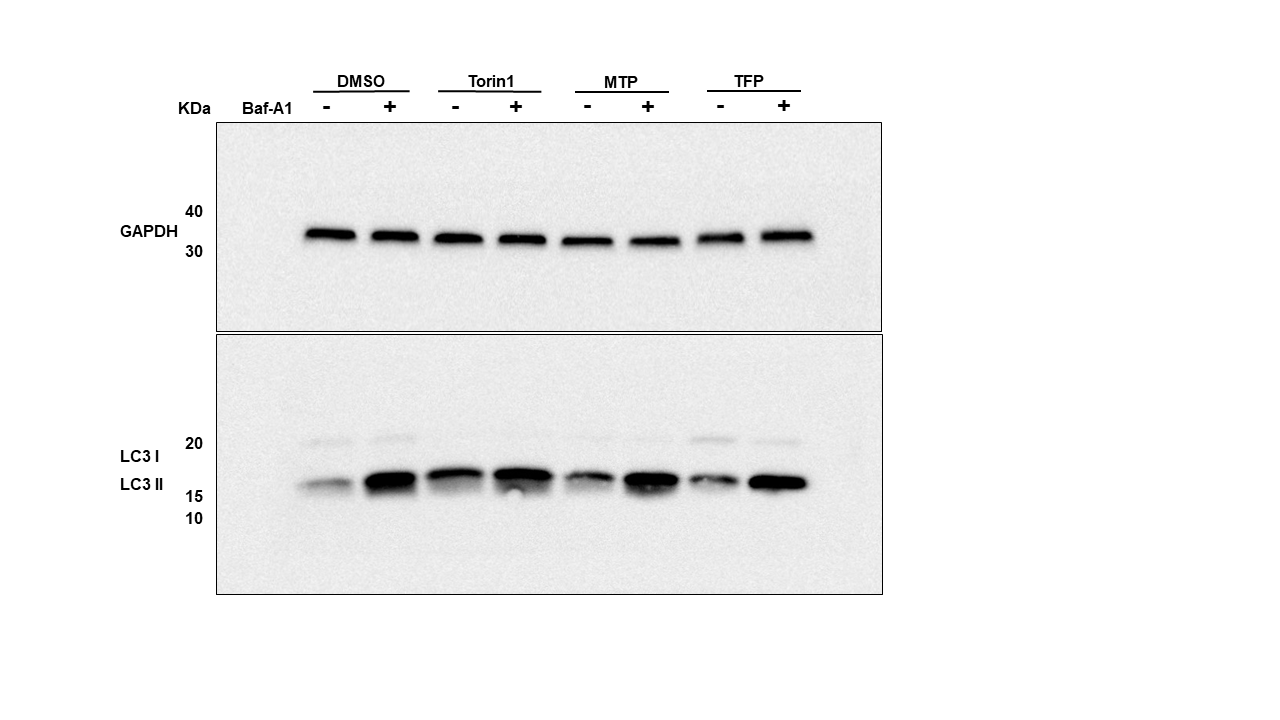

Supplement: Supplementary file 6 — Source Data Fig. 4 [file 44321_2023_14_MOESM6_ESM.zip › Figure 4/Fig 4C/Exp 3.tif]

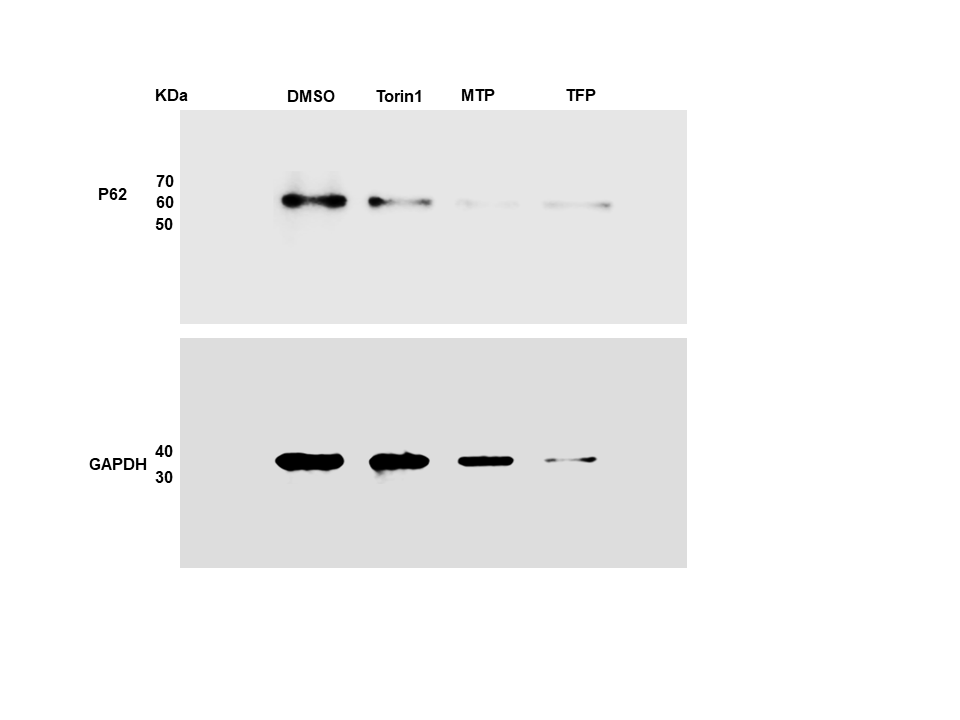

Supplement: Supplementary file 6 — Source Data Fig. 4 [file 44321_2023_14_MOESM6_ESM.zip › Figure 4/Fig 4G/Exp 1.tif]

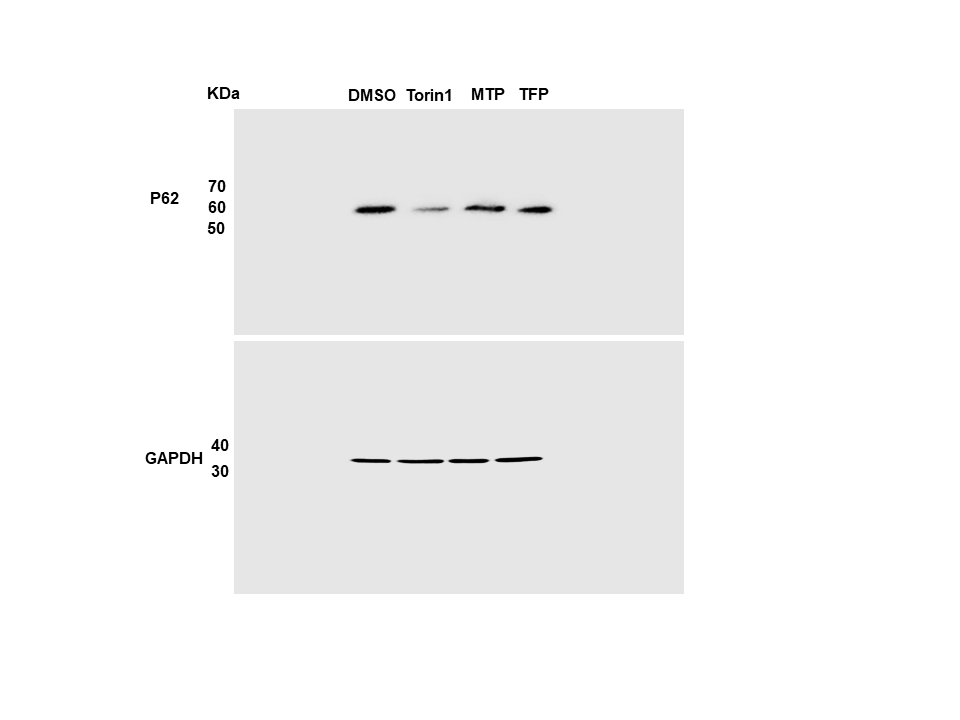

Supplement: Supplementary file 6 — Source Data Fig. 4 [file 44321_2023_14_MOESM6_ESM.zip › Figure 4/Fig 4G/Exp 2.tif]

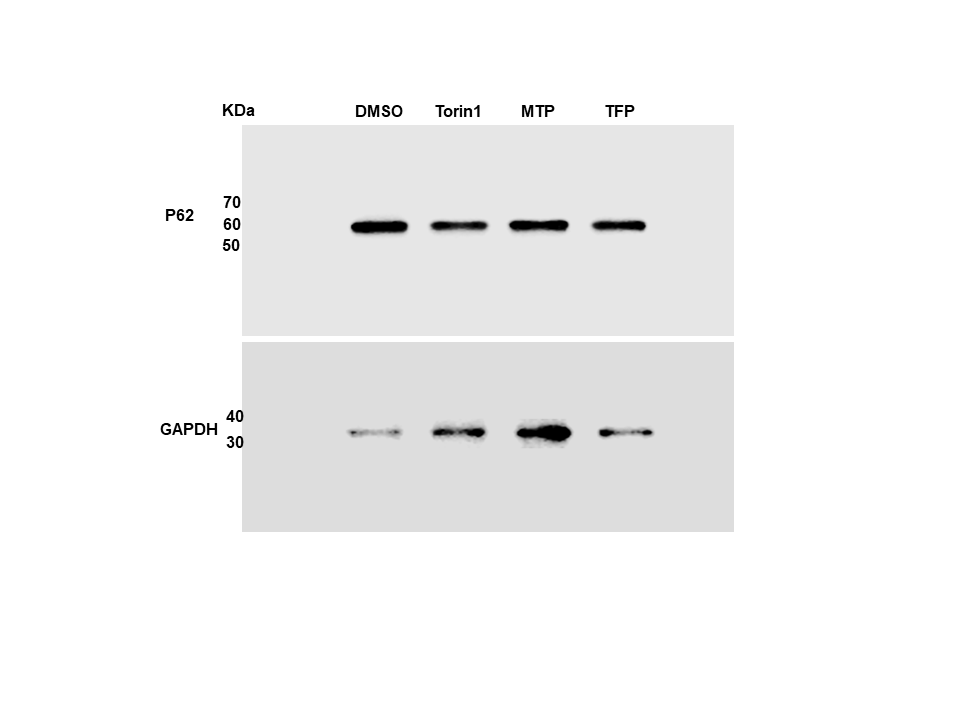

Supplement: Supplementary file 6 — Source Data Fig. 4 [file 44321_2023_14_MOESM6_ESM.zip › Figure 4/Fig 4G/Exp 3.tif]

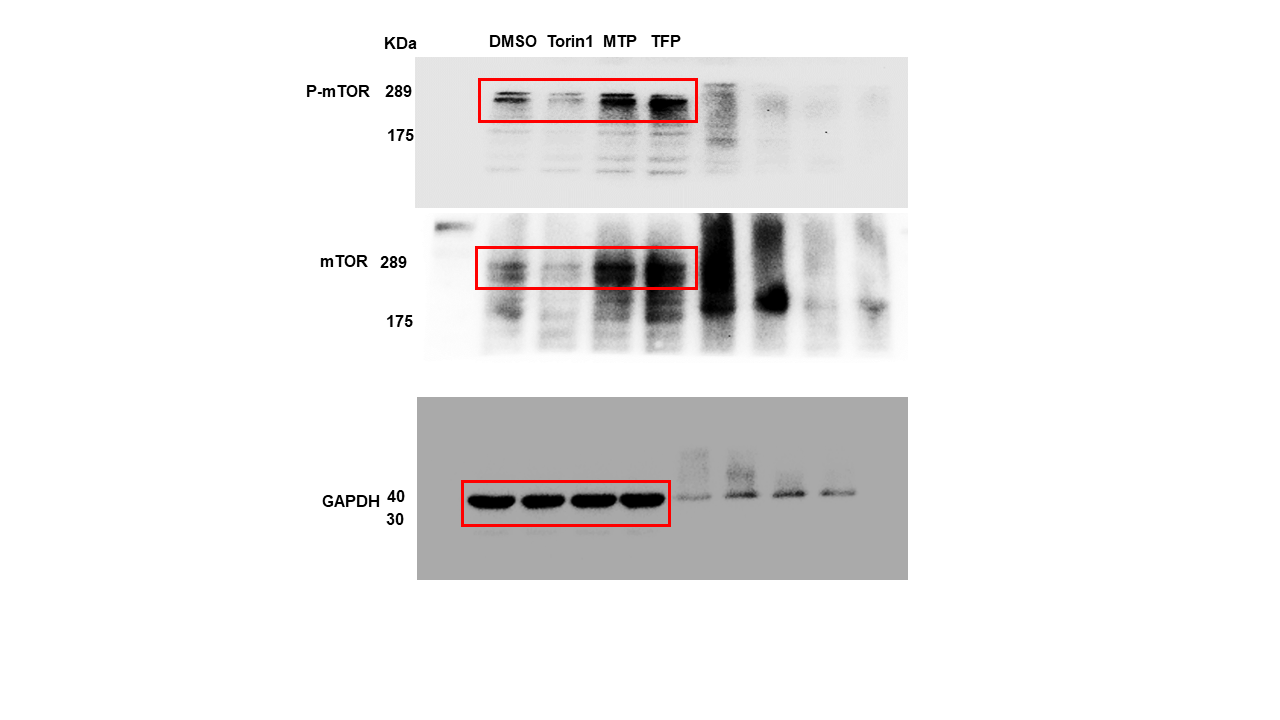

Supplement: Supplementary file 6 — Source Data Fig. 4 [file 44321_2023_14_MOESM6_ESM.zip › Figure 4/Fig 4I/mTOR/Exp 1.tif]

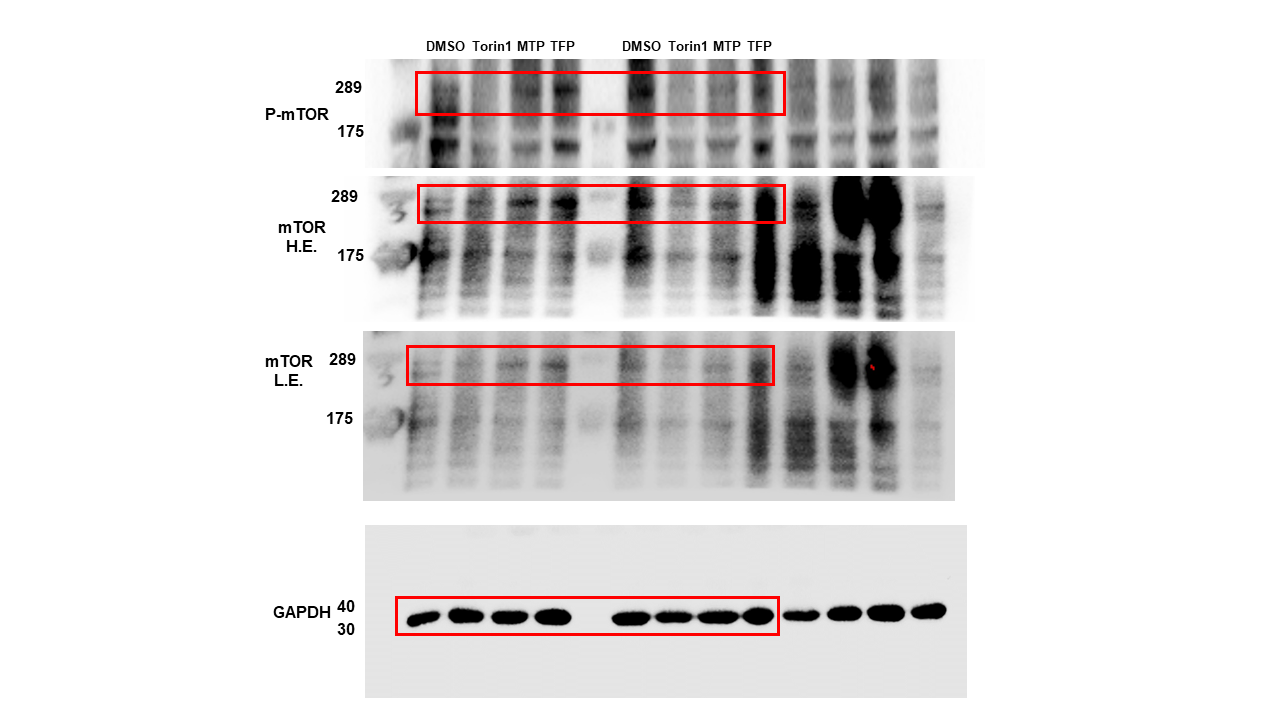

Supplement: Supplementary file 6 — Source Data Fig. 4 [file 44321_2023_14_MOESM6_ESM.zip › Figure 4/Fig 4I/mTOR/Exp 2&3.tif]

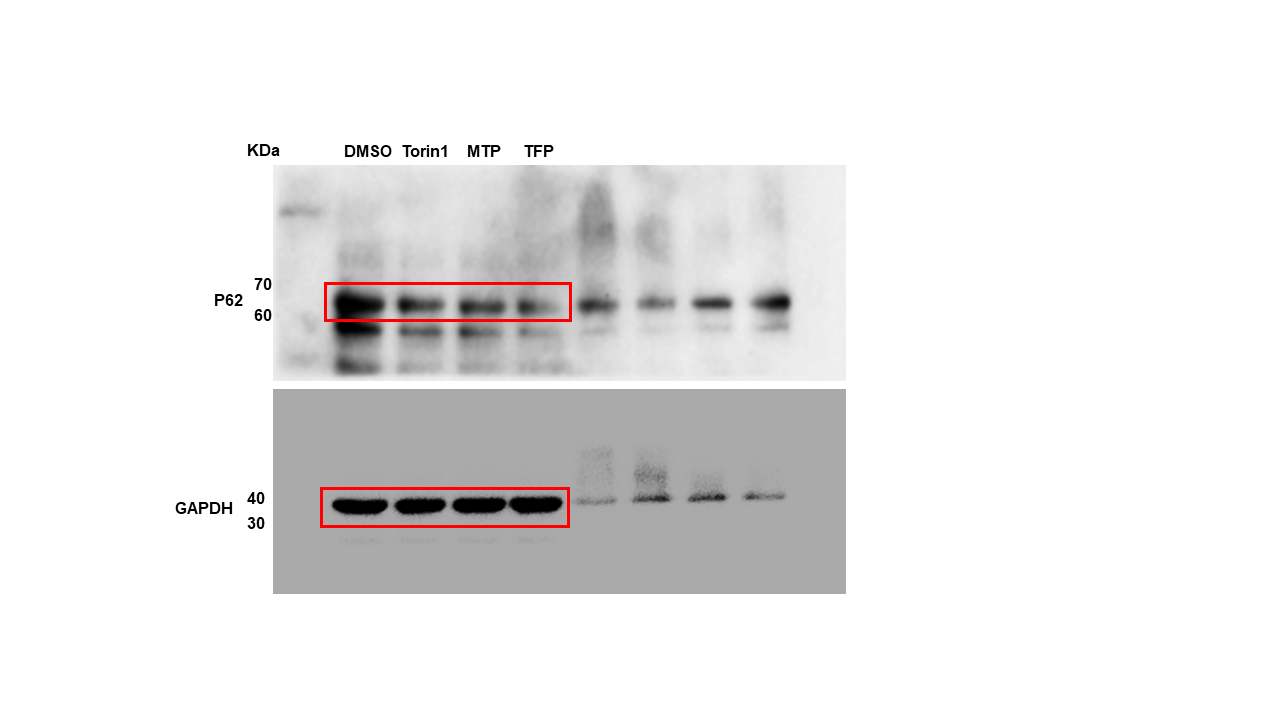

Supplement: Supplementary file 6 — Source Data Fig. 4 [file 44321_2023_14_MOESM6_ESM.zip › Figure 4/Fig 4I/P62/Exp 1.tif]

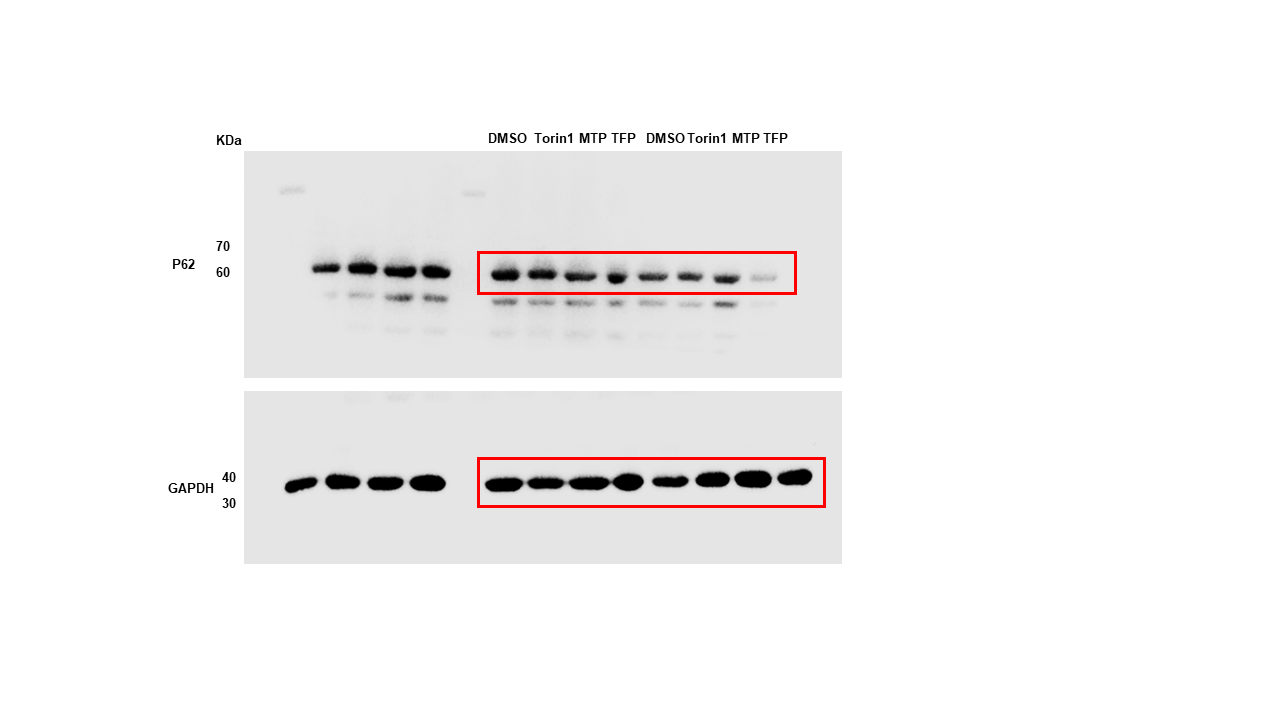

Supplement: Supplementary file 6 — Source Data Fig. 4 [file 44321_2023_14_MOESM6_ESM.zip › Figure 4/Fig 4I/P62/Exp 2&3.tif]

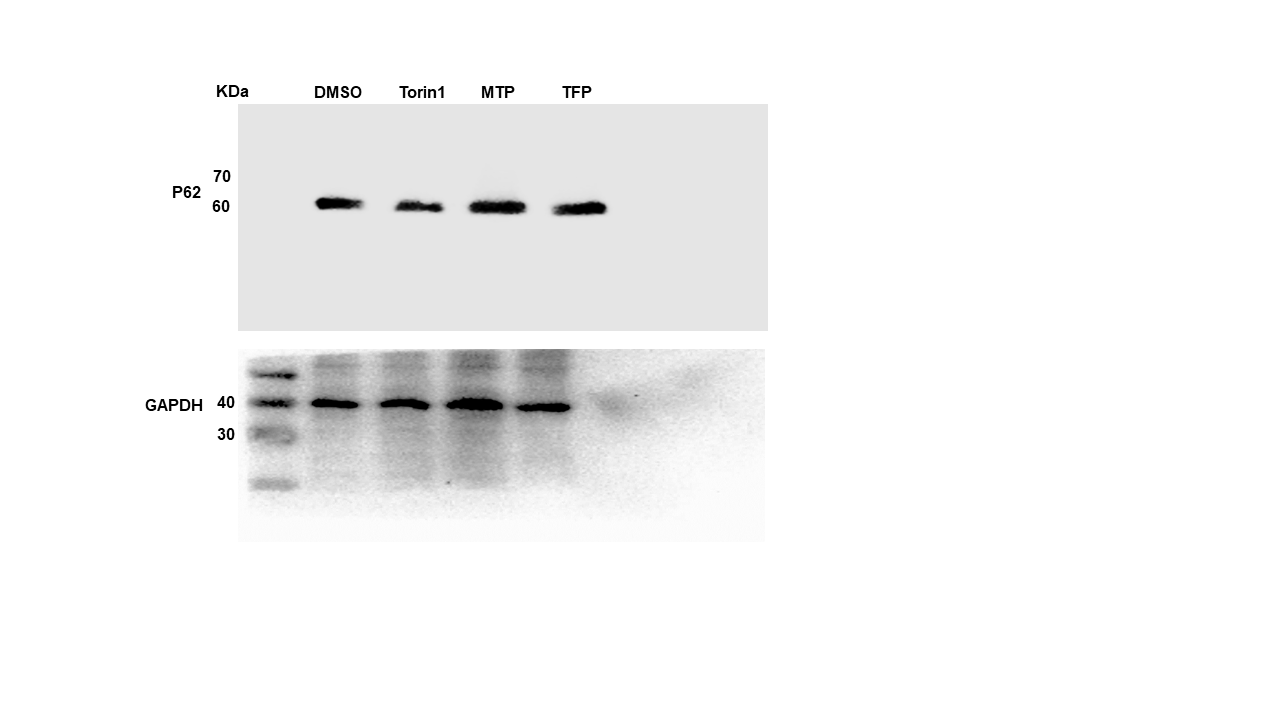

Supplement: Supplementary file 6 — Source Data Fig. 4 [file 44321_2023_14_MOESM6_ESM.zip › Figure 4/Fig 4I/P62/Exp 4.tif]

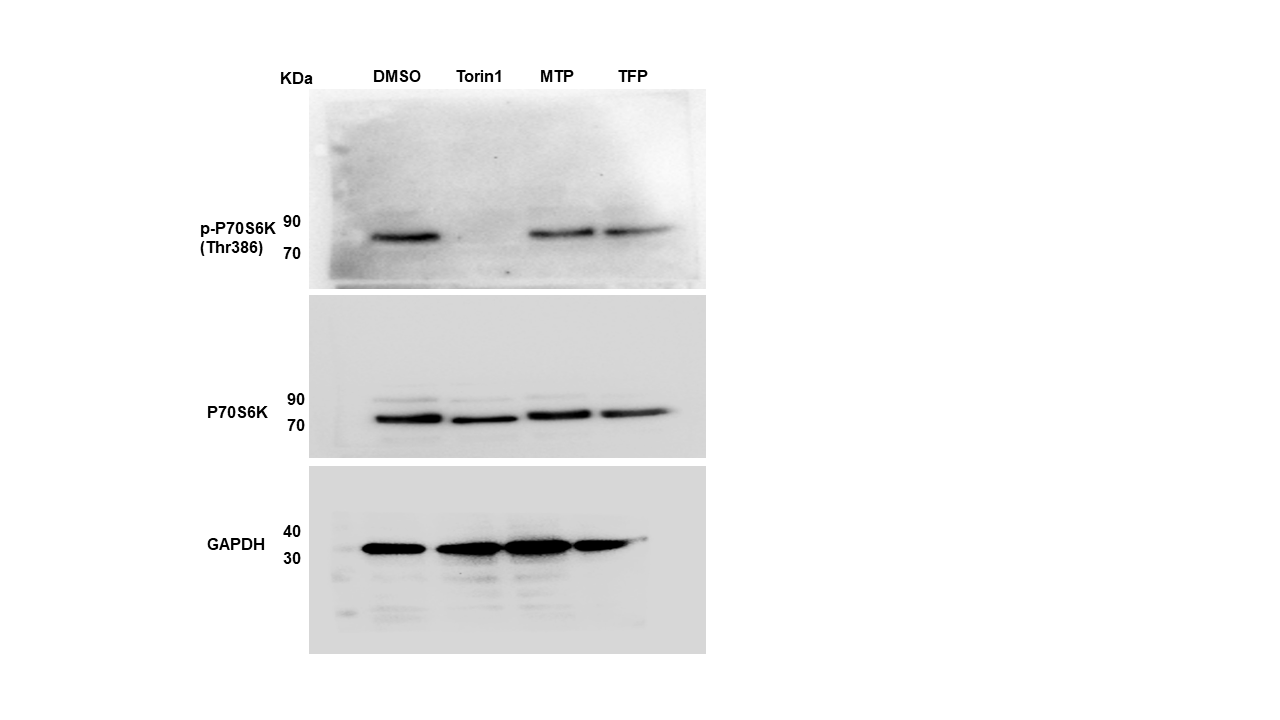

Supplement: Supplementary file 6 — Source Data Fig. 4 [file 44321_2023_14_MOESM6_ESM.zip › Figure 4/Fig 4L/Exp 1.tif]

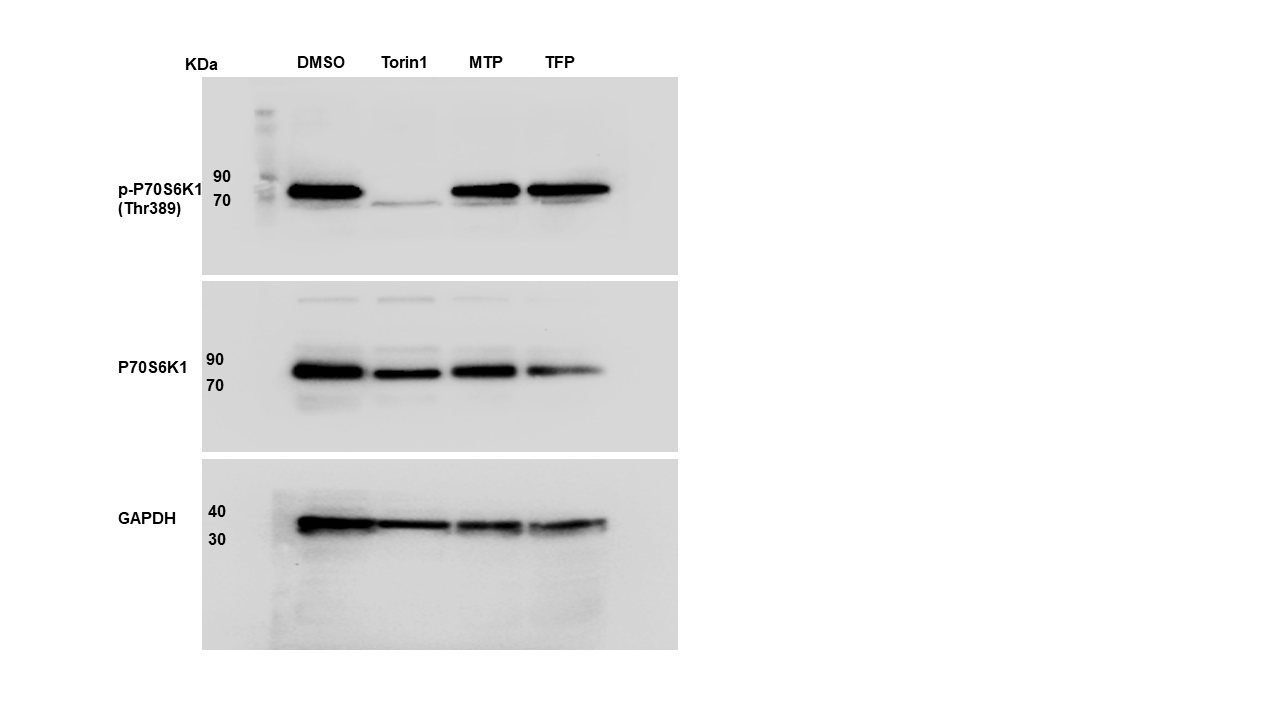

Supplement: Supplementary file 6 — Source Data Fig. 4 [file 44321_2023_14_MOESM6_ESM.zip › Figure 4/Fig 4L/Exp 2.tif]

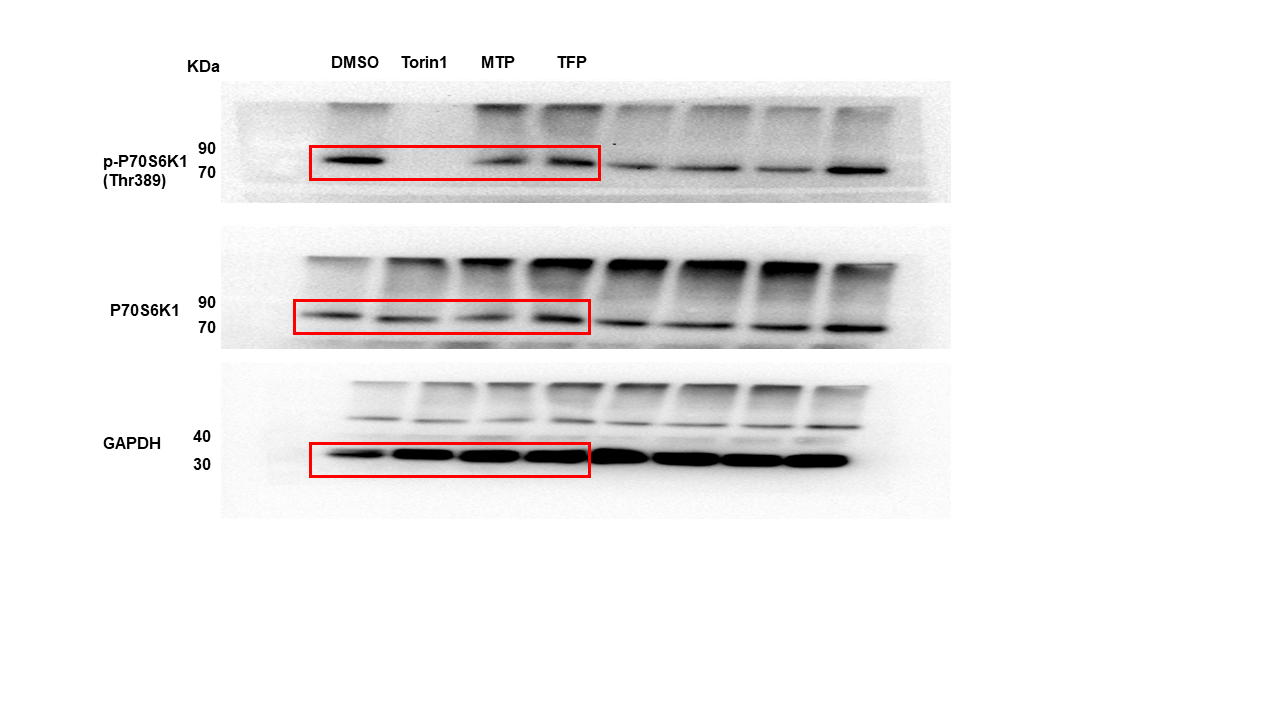

Supplement: Supplementary file 6 — Source Data Fig. 4 [file 44321_2023_14_MOESM6_ESM.zip › Figure 4/Fig 4L/Exp 3.tif]

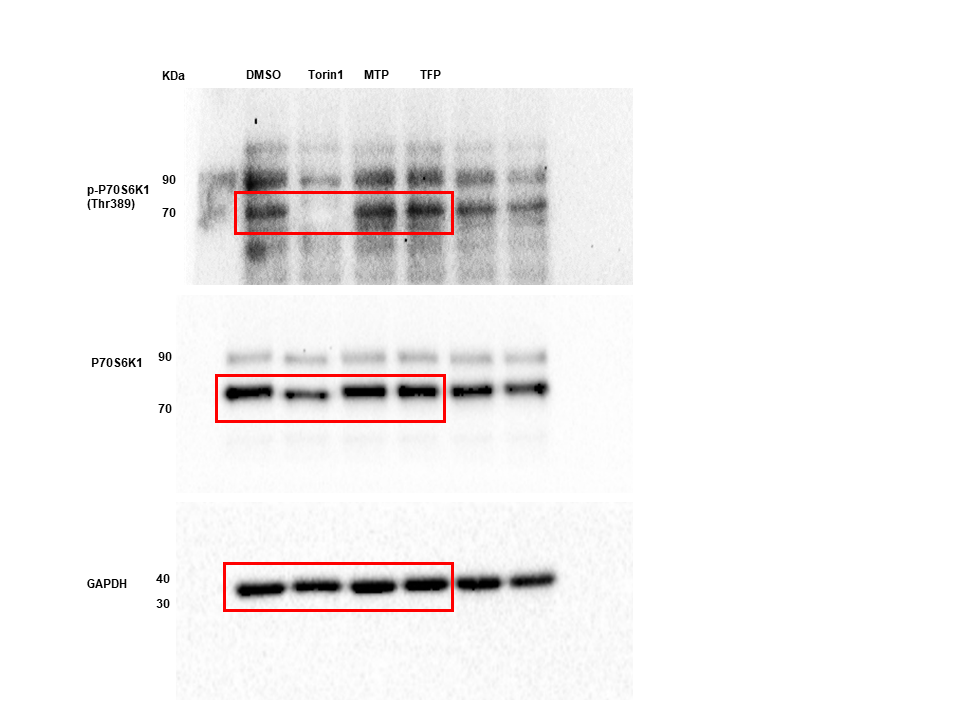

Supplement: Supplementary file 6 — Source Data Fig. 4 [file 44321_2023_14_MOESM6_ESM.zip › Figure 4/Fig 4L/Exp 4.tif]

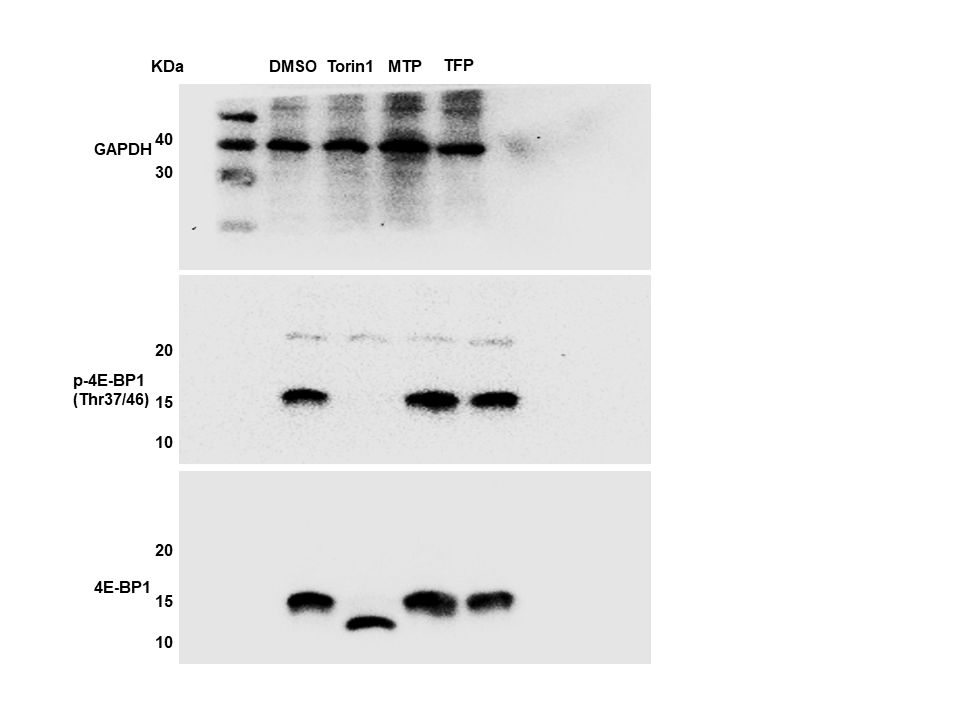

Supplement: Supplementary file 6 — Source Data Fig. 4 [file 44321_2023_14_MOESM6_ESM.zip › Figure 4/Fig 4N/Exp 1.tif]

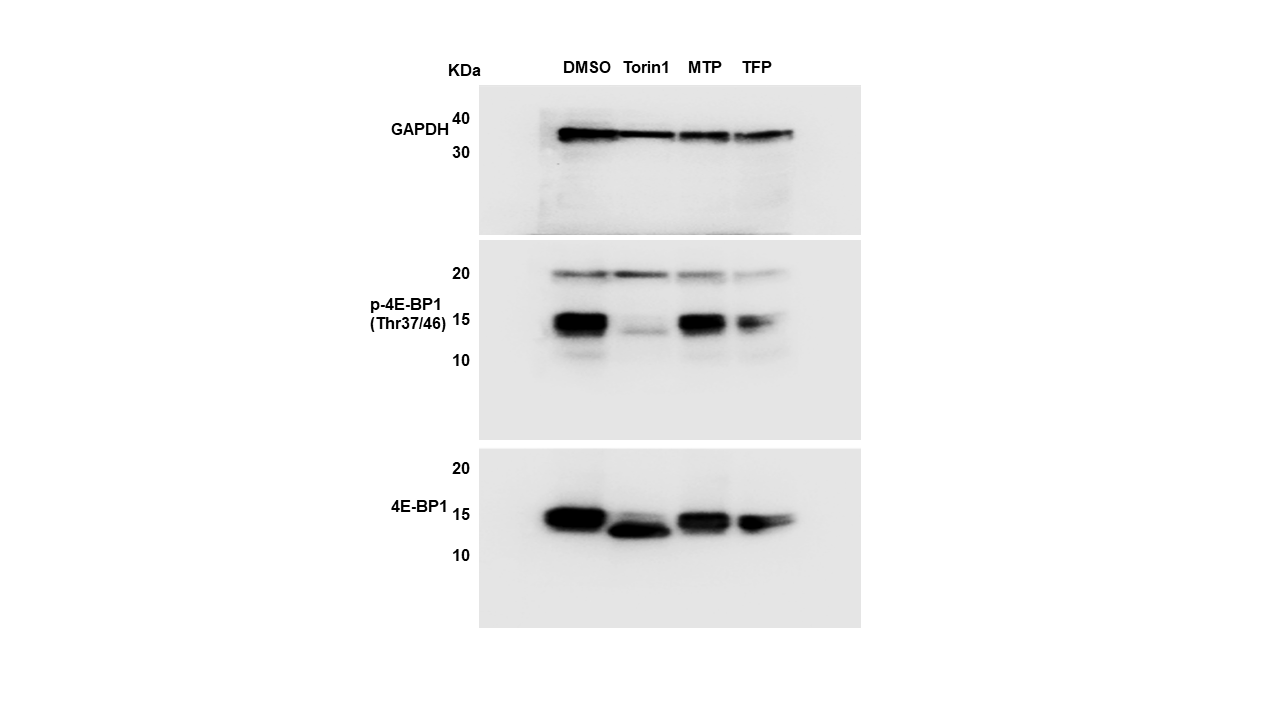

Supplement: Supplementary file 6 — Source Data Fig. 4 [file 44321_2023_14_MOESM6_ESM.zip › Figure 4/Fig 4N/Exp 2.tif]

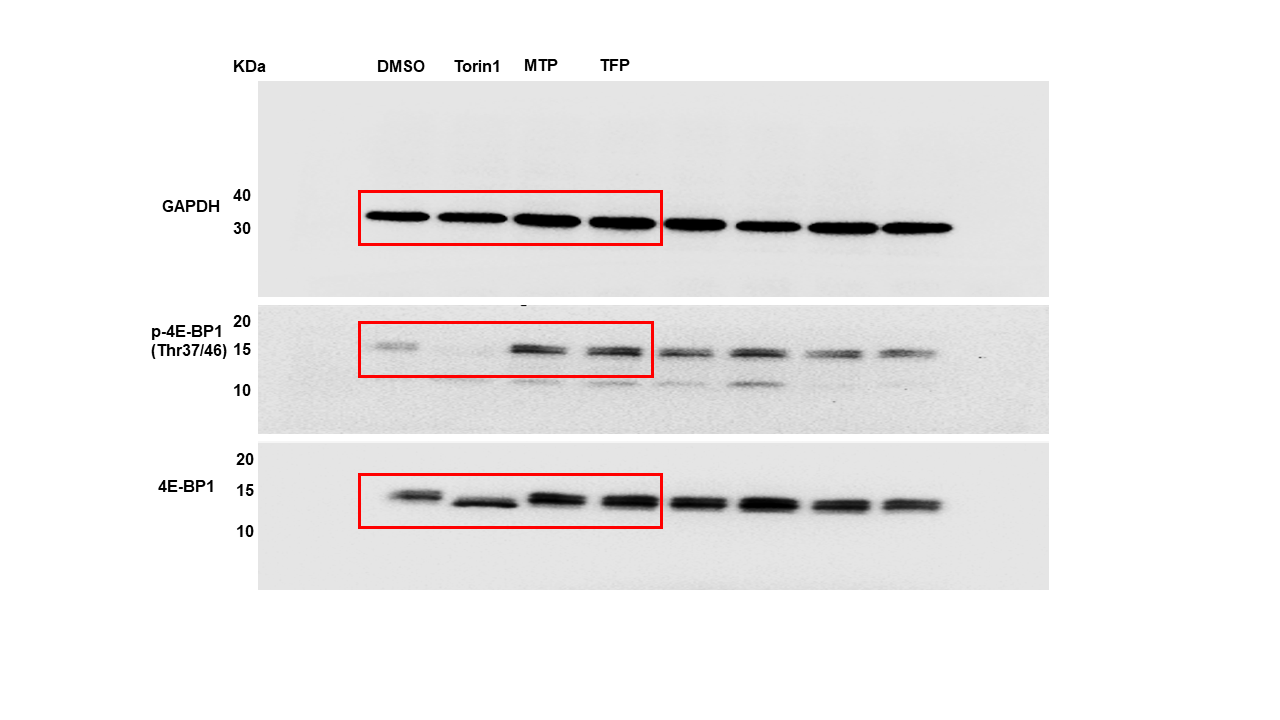

Supplement: Supplementary file 6 — Source Data Fig. 4 [file 44321_2023_14_MOESM6_ESM.zip › Figure 4/Fig 4N/Exp 3.tif]

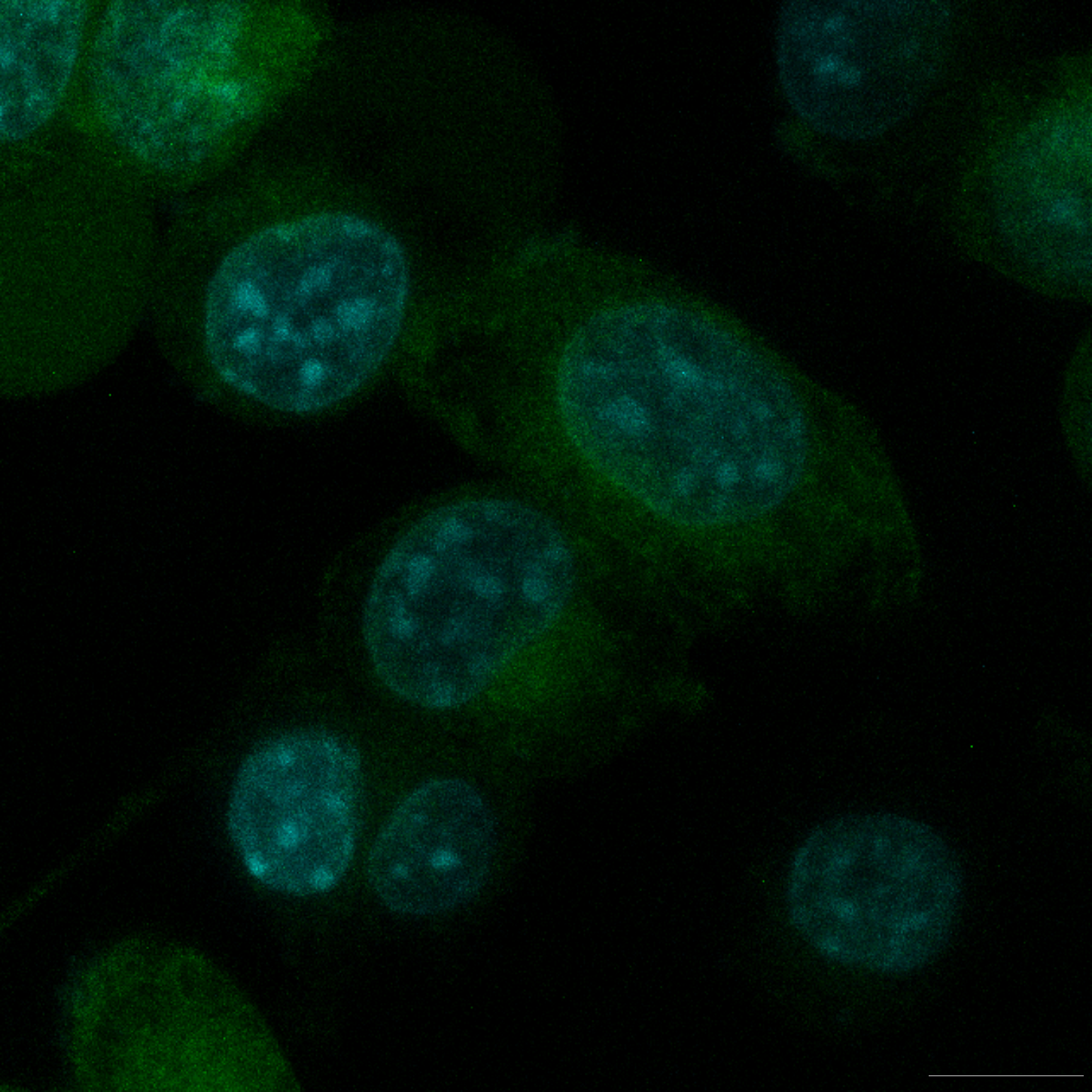

Supplement: Supplementary file 6 — Source Data Fig. 4 [file 44321_2023_14_MOESM6_ESM.zip › Figure 4/Fig 4P/DMSO.tif]

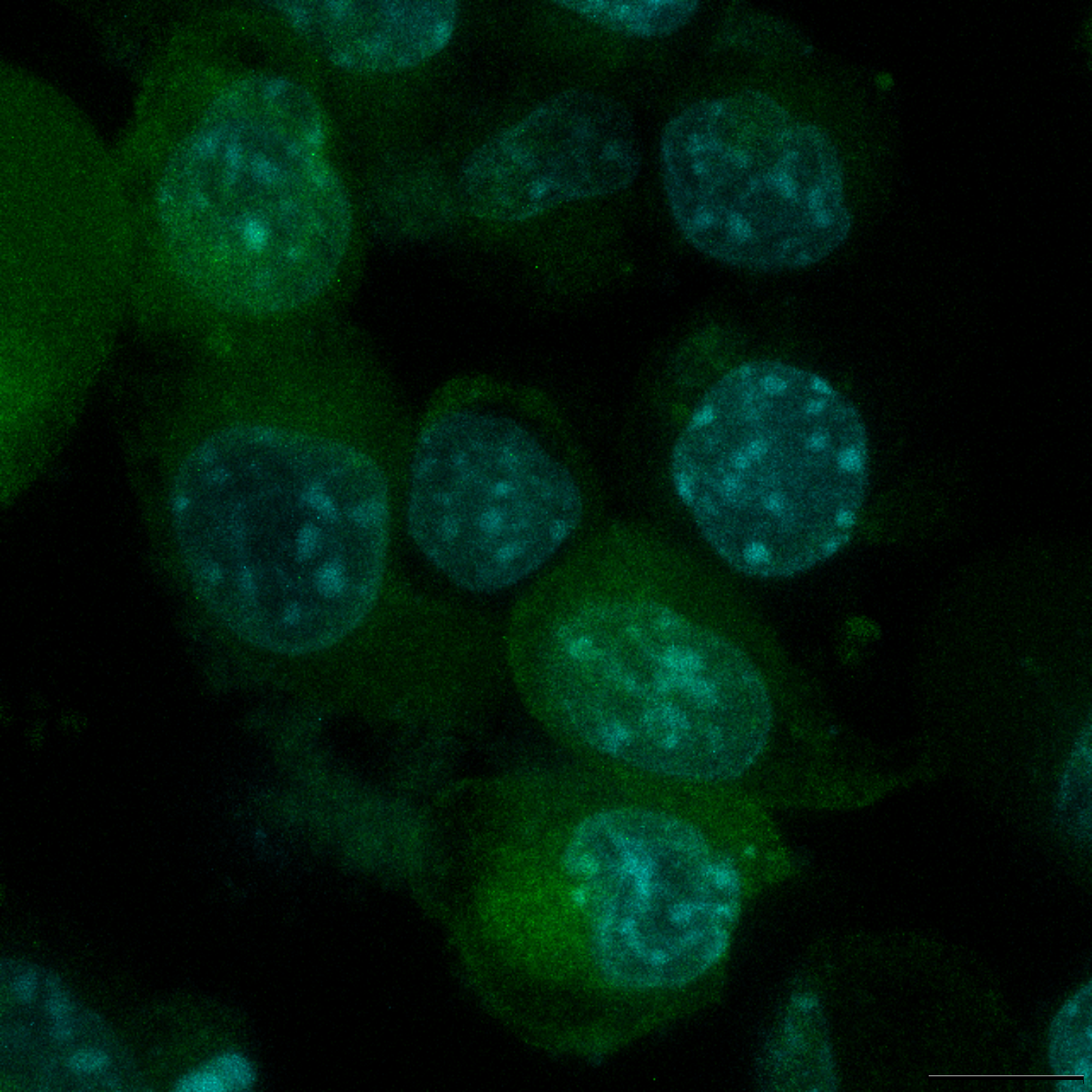

Supplement: Supplementary file 6 — Source Data Fig. 4 [file 44321_2023_14_MOESM6_ESM.zip › Figure 4/Fig 4P/MTP.tif]

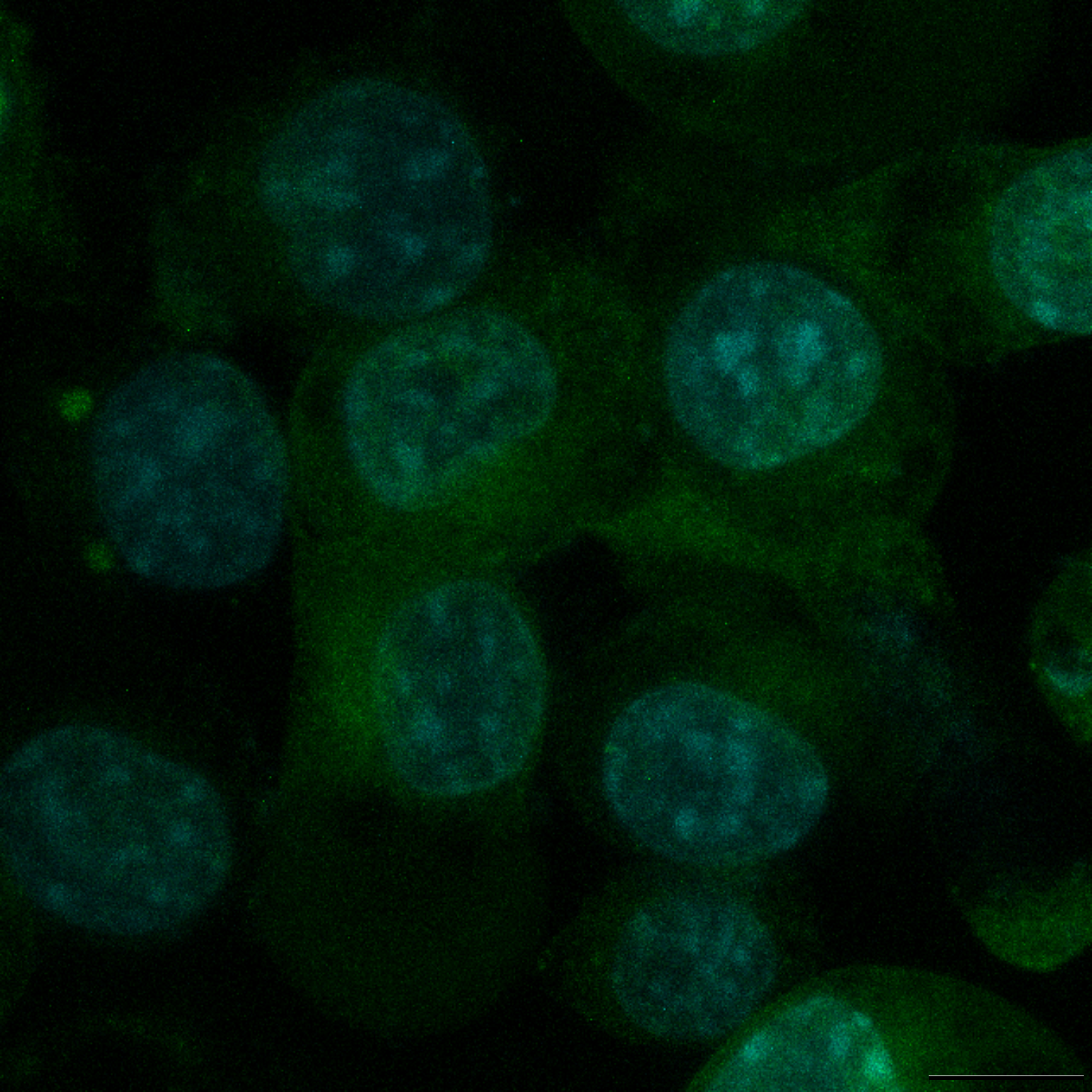

Supplement: Supplementary file 6 — Source Data Fig. 4 [file 44321_2023_14_MOESM6_ESM.zip › Figure 4/Fig 4P/TFP.tif]

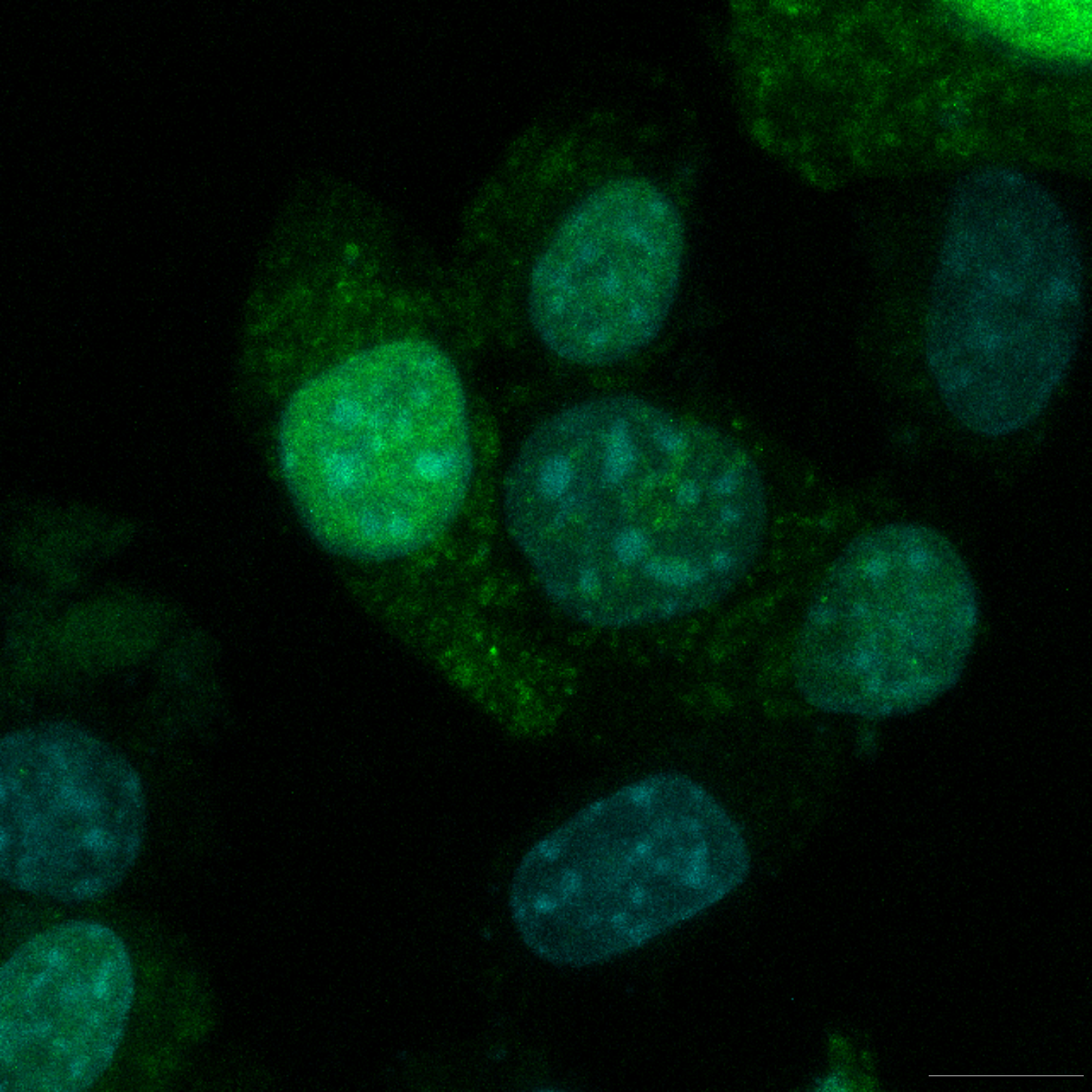

Supplement: Supplementary file 6 — Source Data Fig. 4 [file 44321_2023_14_MOESM6_ESM.zip › Figure 4/Fig 4P/Torin1.tif]

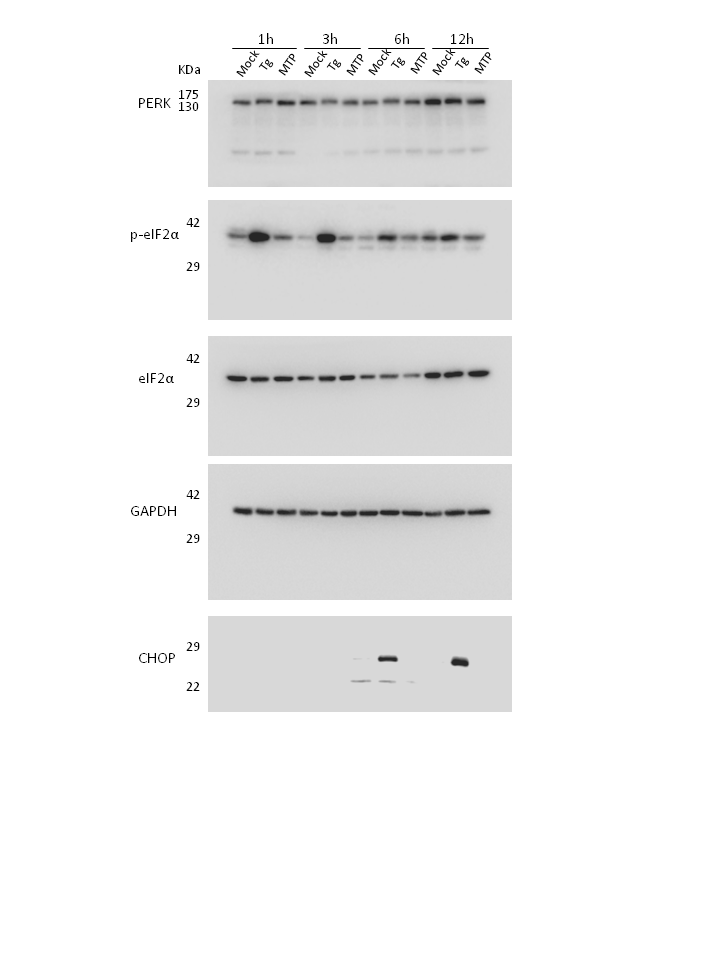

Supplement: Supplementary file 7 — Source Data Fig. 5 [file 44321_2023_14_MOESM7_ESM.zip › Figure 5/Fig 5B/Exp 1.tif]

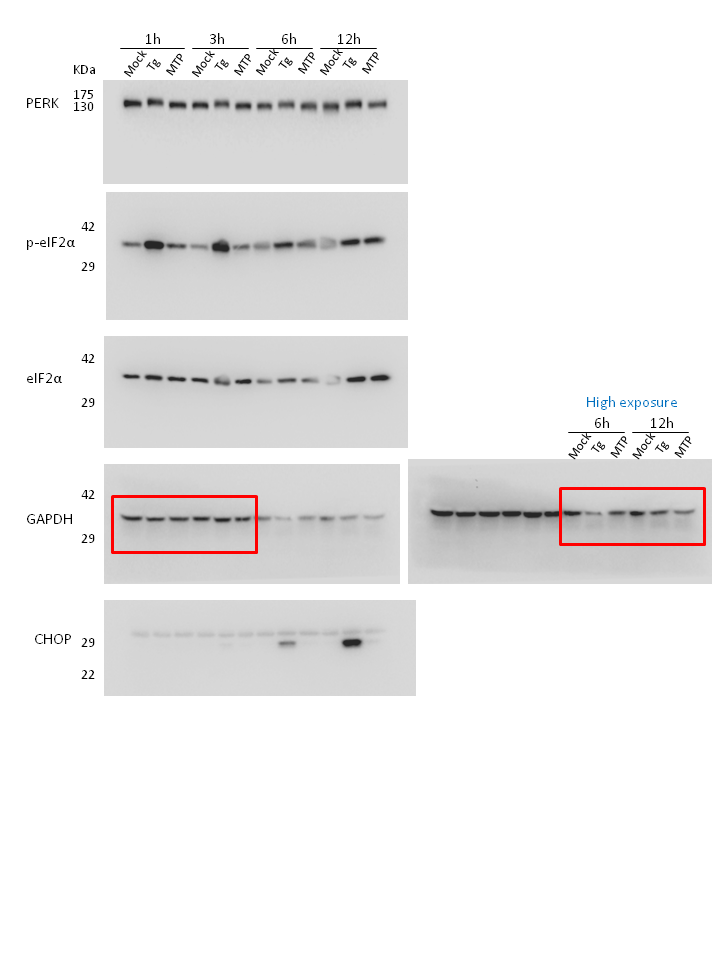

Supplement: Supplementary file 7 — Source Data Fig. 5 [file 44321_2023_14_MOESM7_ESM.zip › Figure 5/Fig 5B/Exp 2.tif]

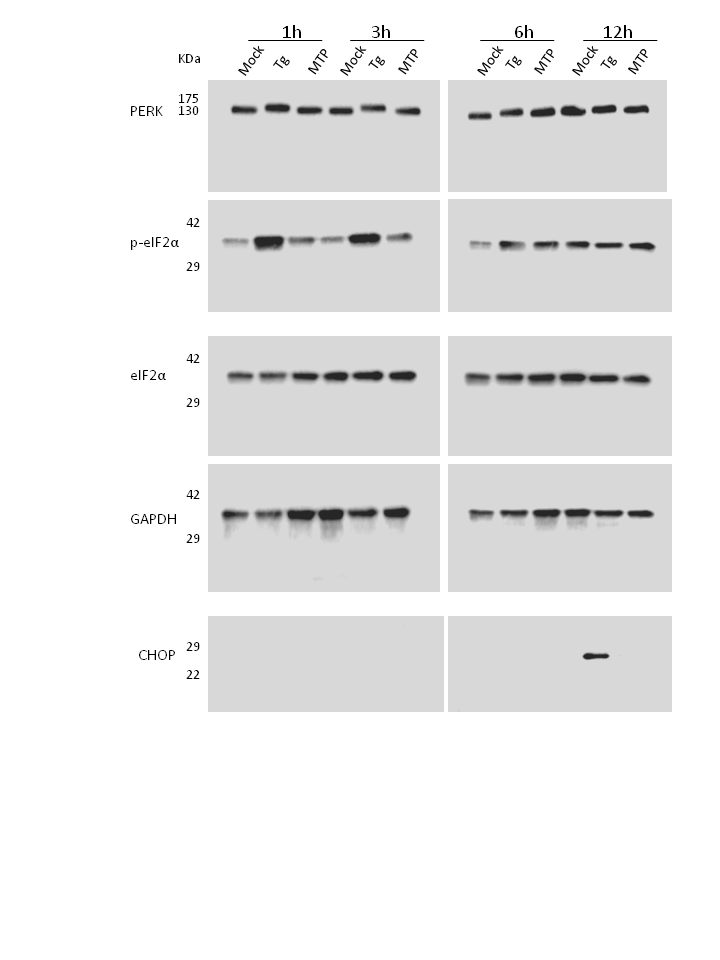

Supplement: Supplementary file 7 — Source Data Fig. 5 [file 44321_2023_14_MOESM7_ESM.zip › Figure 5/Fig 5B/Exp 3.tif]

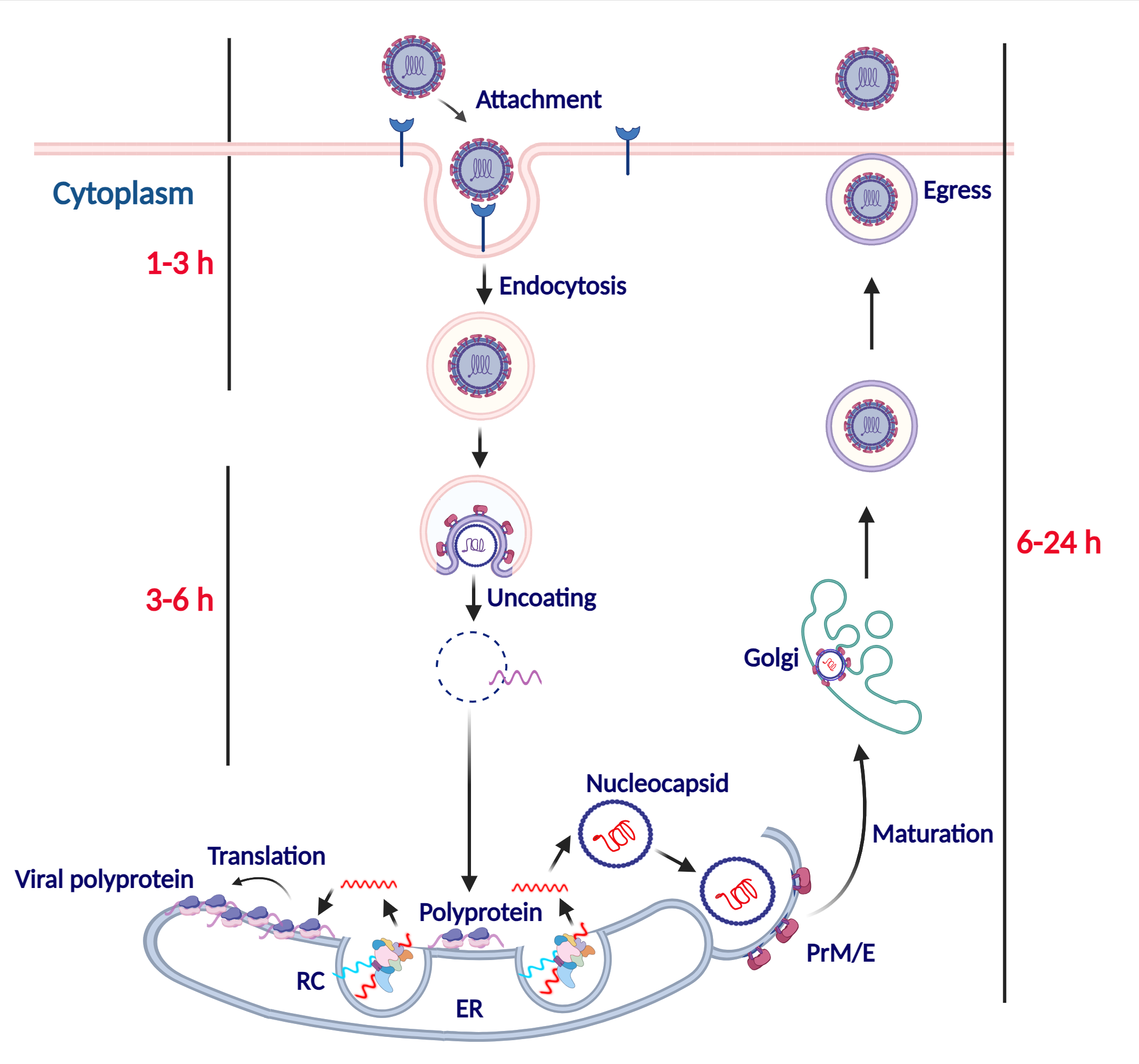

Supplement: Supplementary file 8 — Source Data Fig. 6 [file 44321_2023_14_MOESM8_ESM.zip › Figure 6/Fig 6A/Life Cycle of JEV.png]

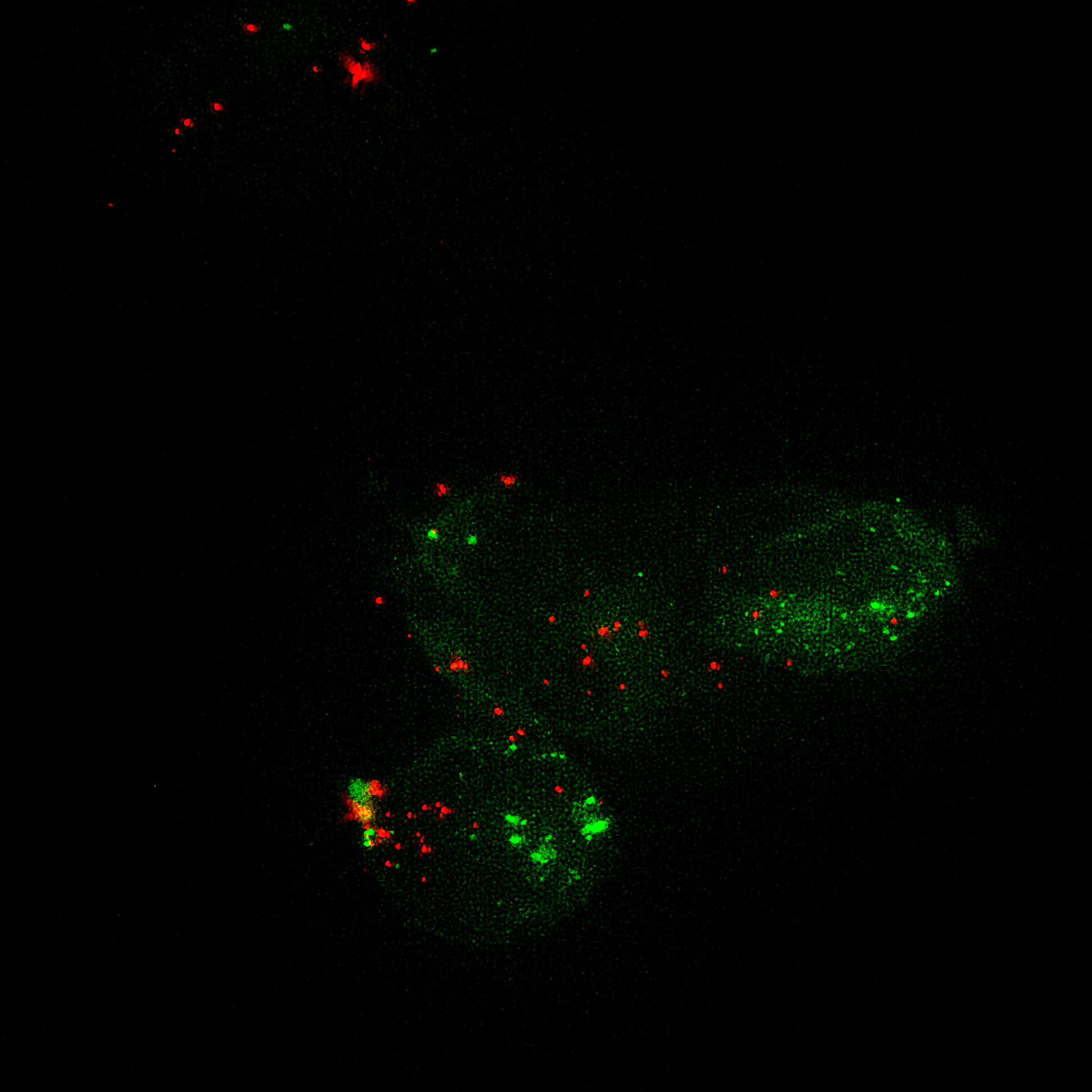

Supplement: Supplementary file 8 — Source Data Fig. 6 [file 44321_2023_14_MOESM8_ESM.zip › Figure 6/Fig 6D/JEV/Image 1_Out_Maximum intensity projection.tif]

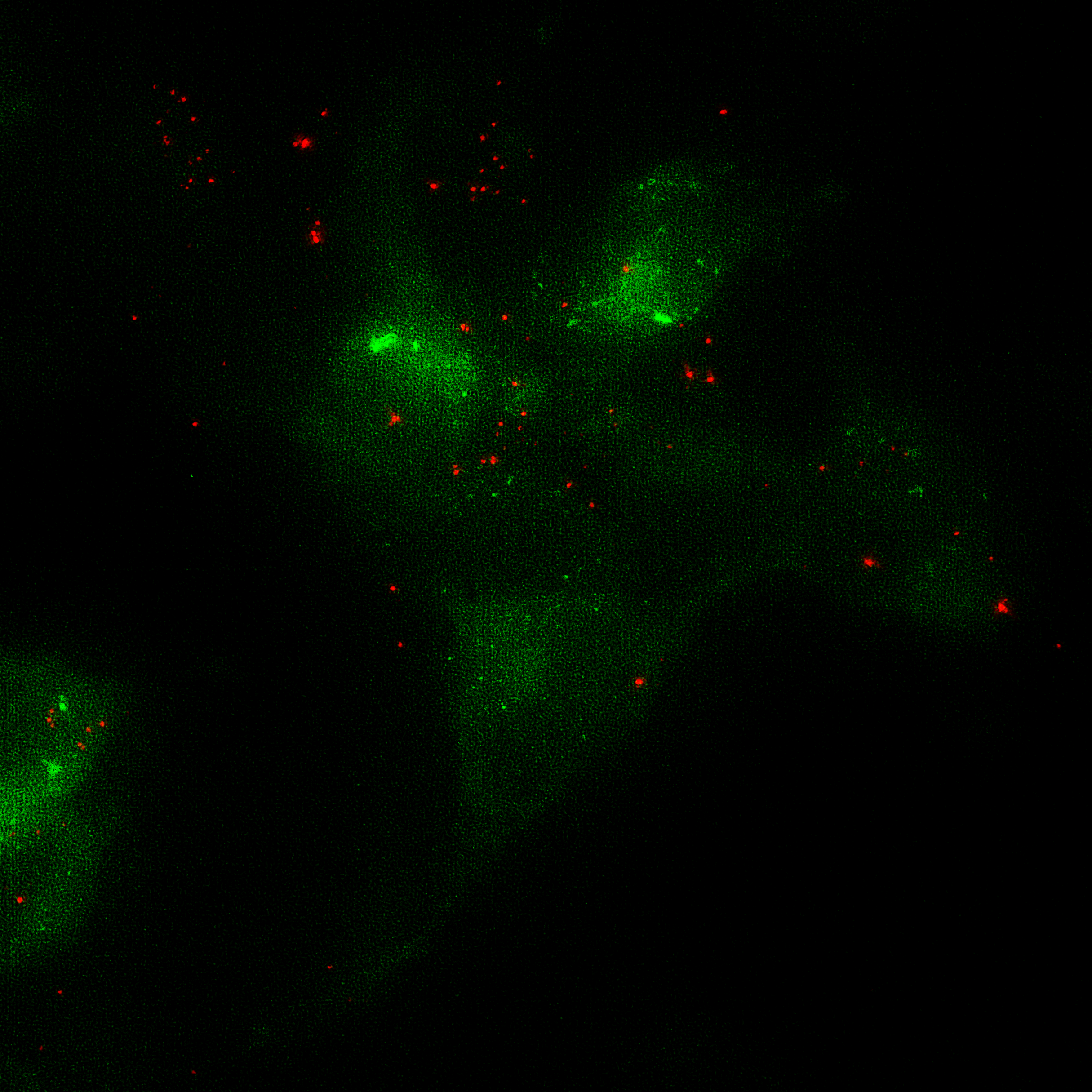

Supplement: Supplementary file 8 — Source Data Fig. 6 [file 44321_2023_14_MOESM8_ESM.zip › Figure 6/Fig 6D/JEV/Image 2_Out_Maximum intensity projection.tif]

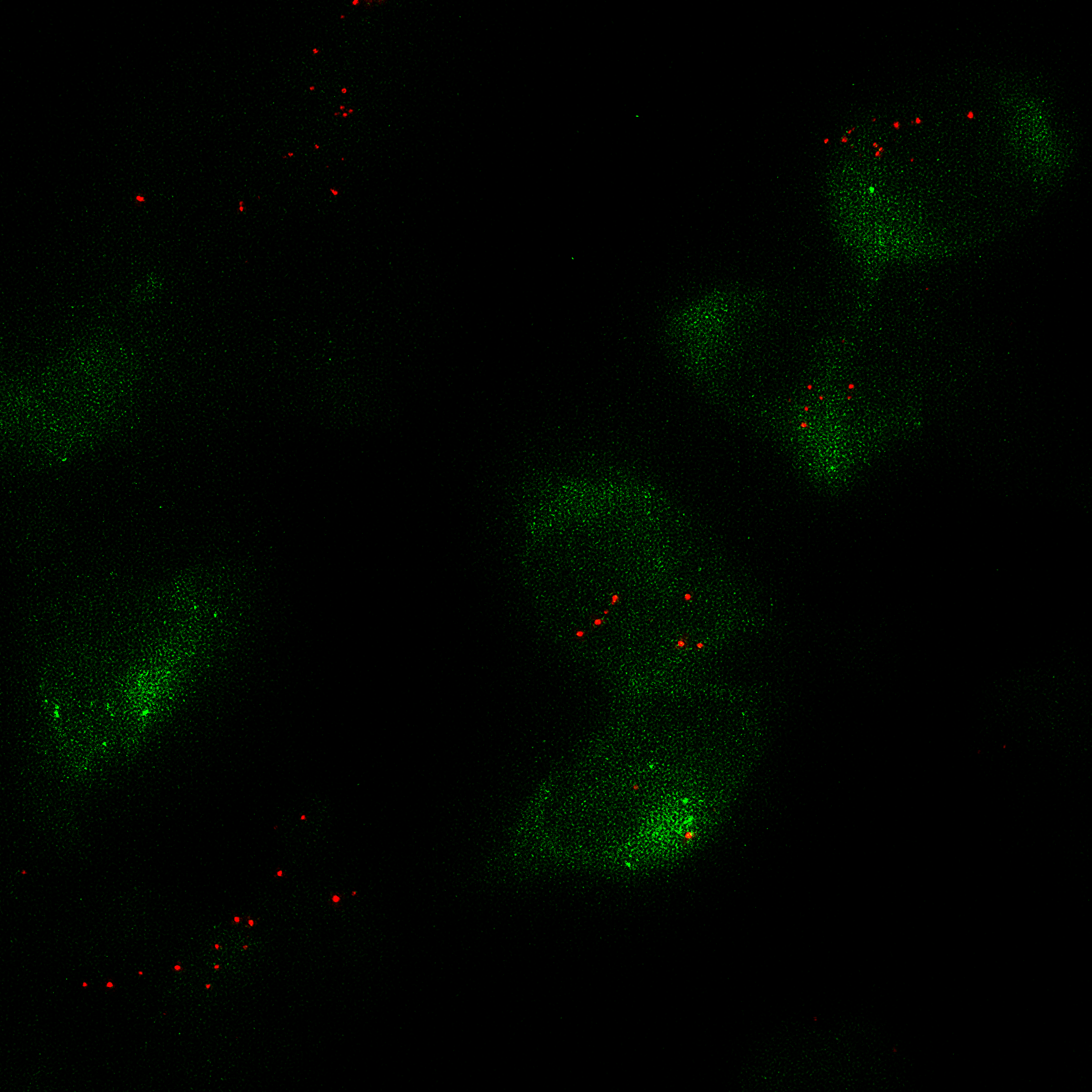

Supplement: Supplementary file 8 — Source Data Fig. 6 [file 44321_2023_14_MOESM8_ESM.zip › Figure 6/Fig 6D/JEV/Image 3_Out_Maximum intensity projection.tif]

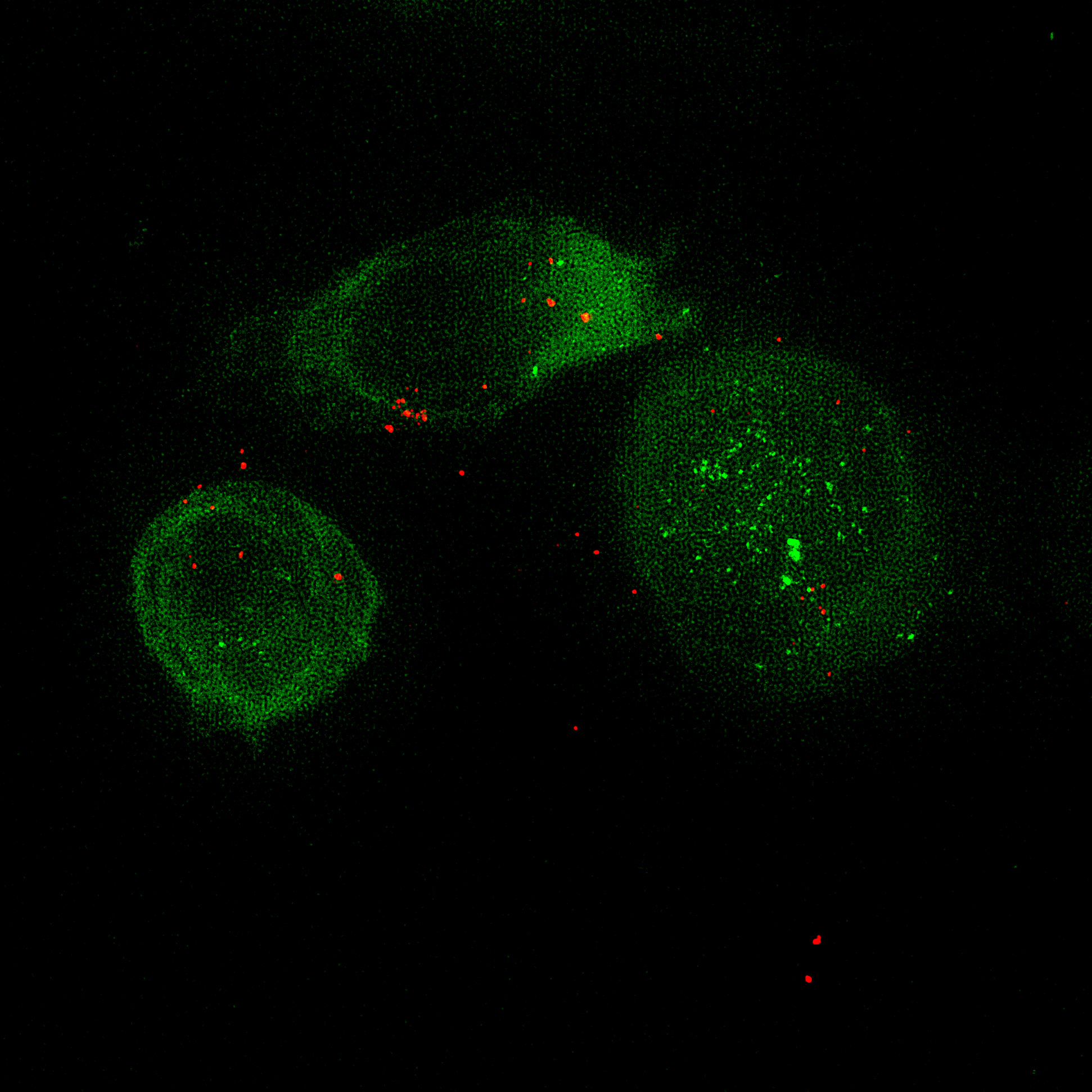

Supplement: Supplementary file 8 — Source Data Fig. 6 [file 44321_2023_14_MOESM8_ESM.zip › Figure 6/Fig 6D/JEV/Image 4_Out_ JEV.tif]

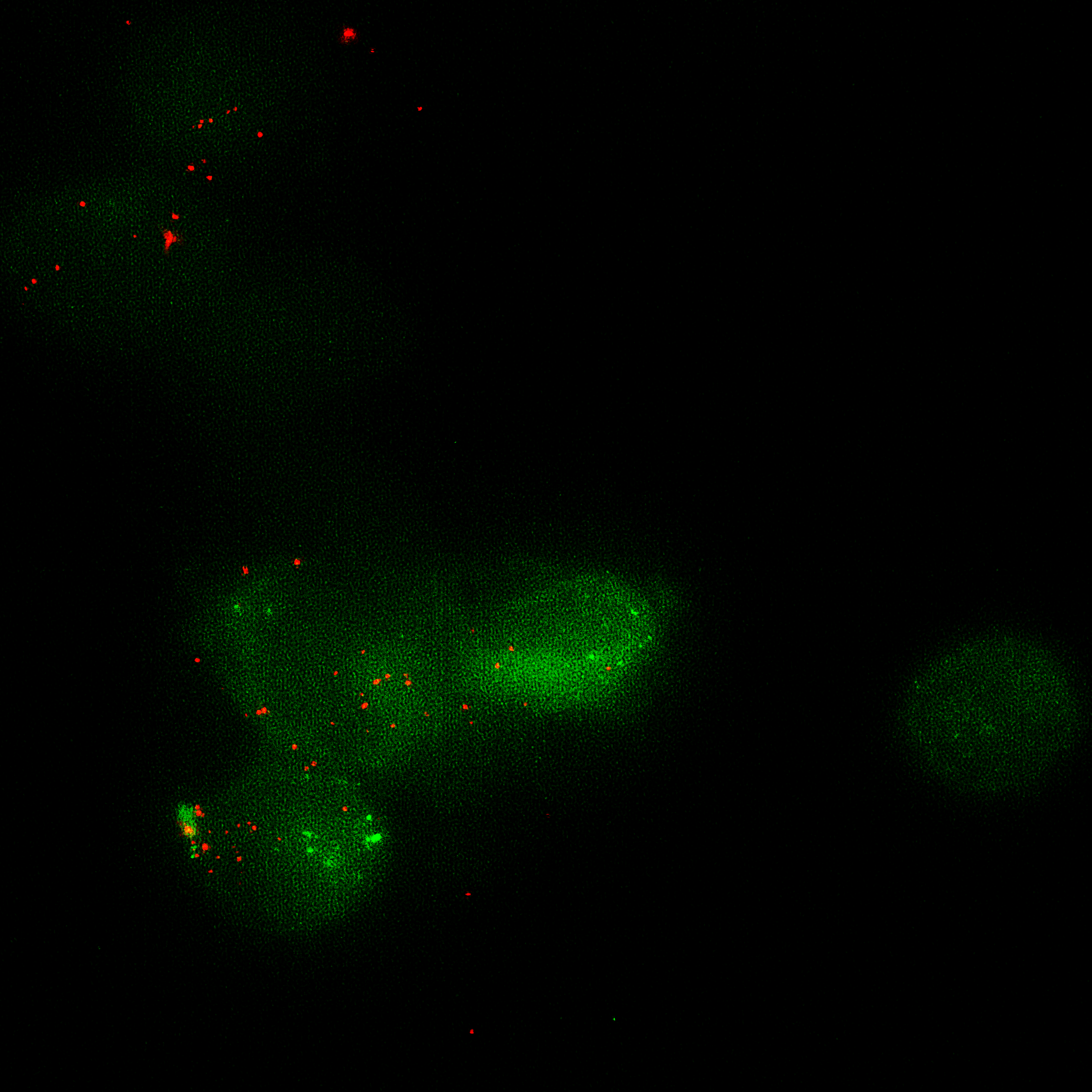

Supplement: Supplementary file 8 — Source Data Fig. 6 [file 44321_2023_14_MOESM8_ESM.zip › Figure 6/Fig 6D/JEV/Image 4_Out_Maximum intensity projection.tif]

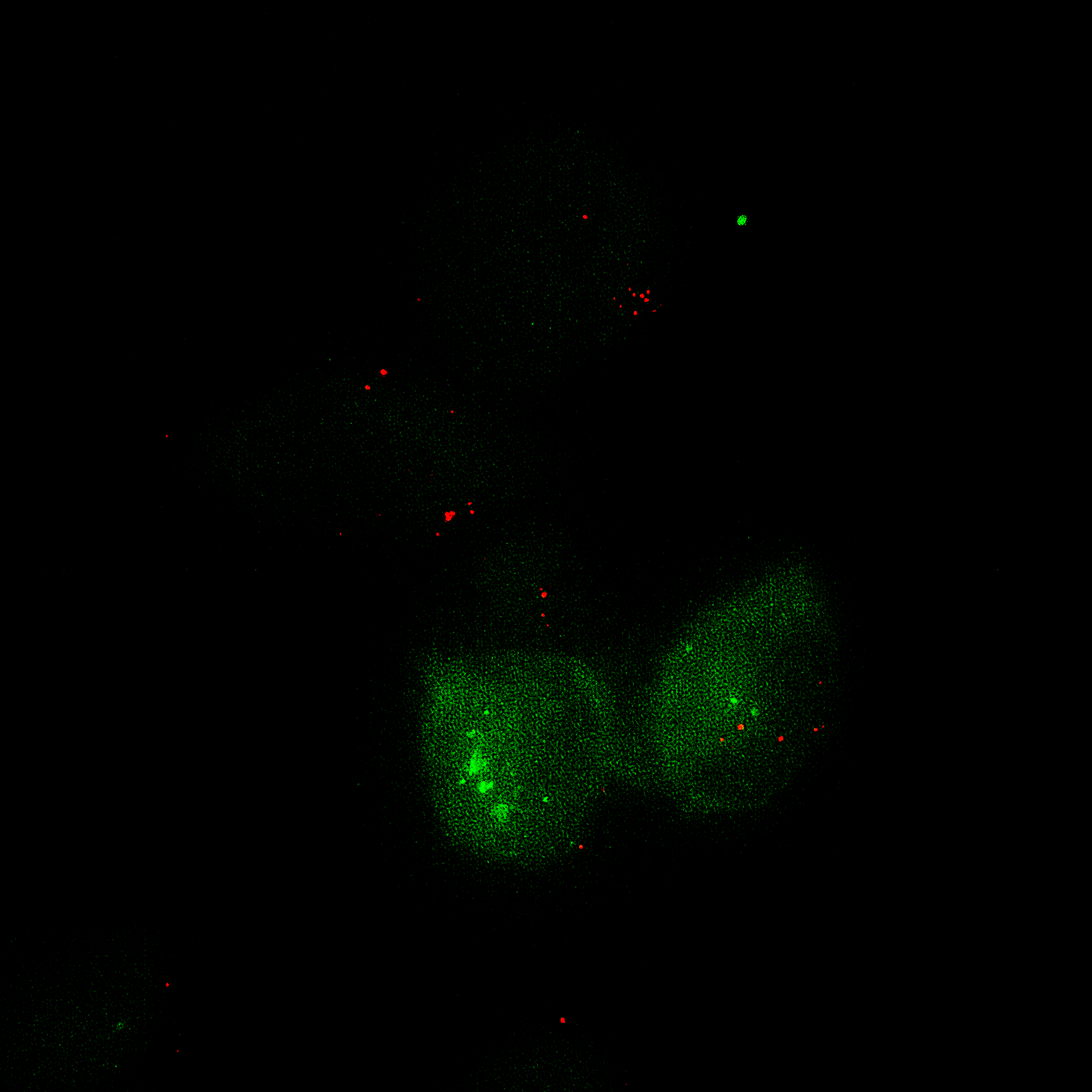

Supplement: Supplementary file 8 — Source Data Fig. 6 [file 44321_2023_14_MOESM8_ESM.zip › Figure 6/Fig 6D/JEV/Image 6_Out_Maximum intensity projection.tif]

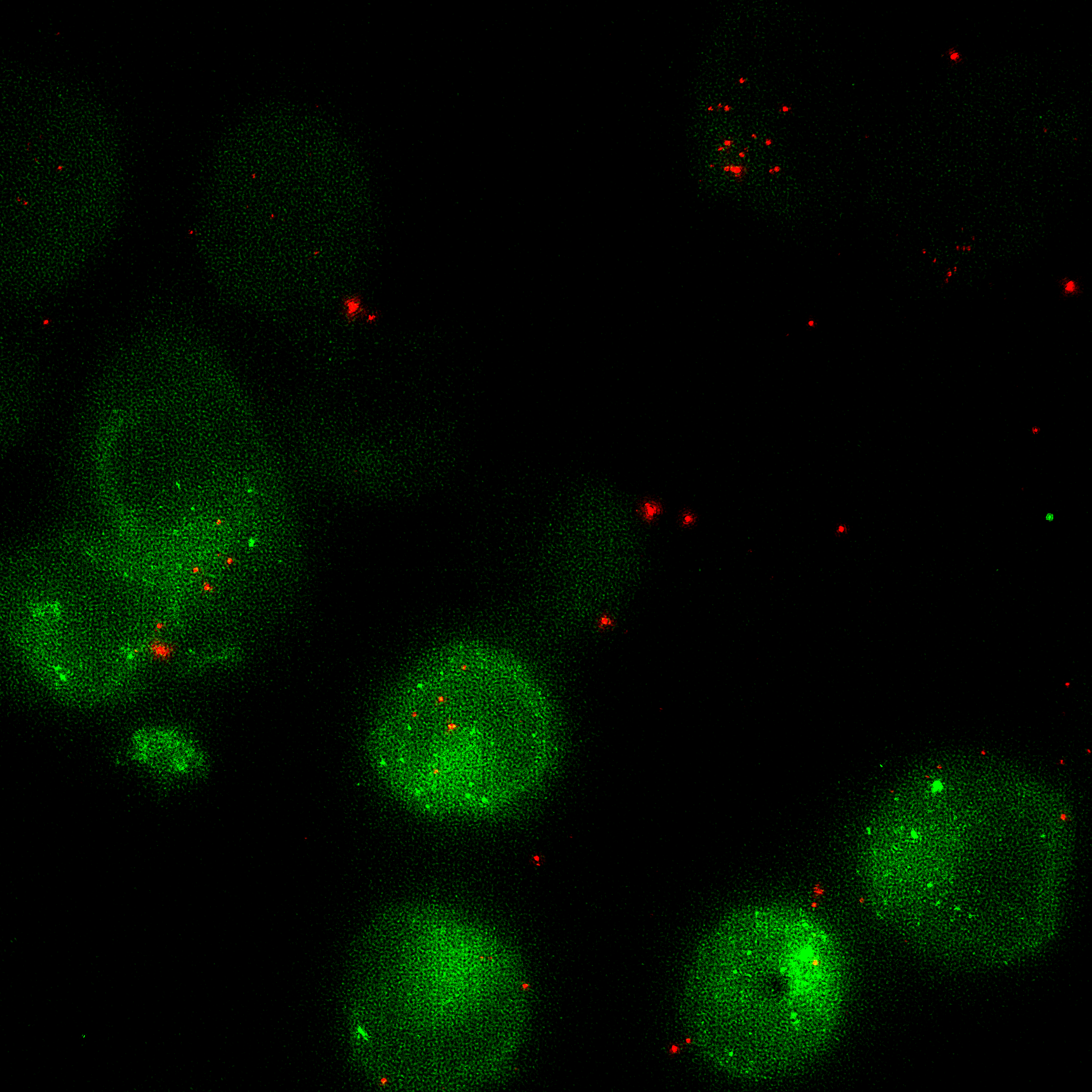

Supplement: Supplementary file 8 — Source Data Fig. 6 [file 44321_2023_14_MOESM8_ESM.zip › Figure 6/Fig 6D/JEV+MTP/Image 12_Out_Maximum intensity projection.tif]

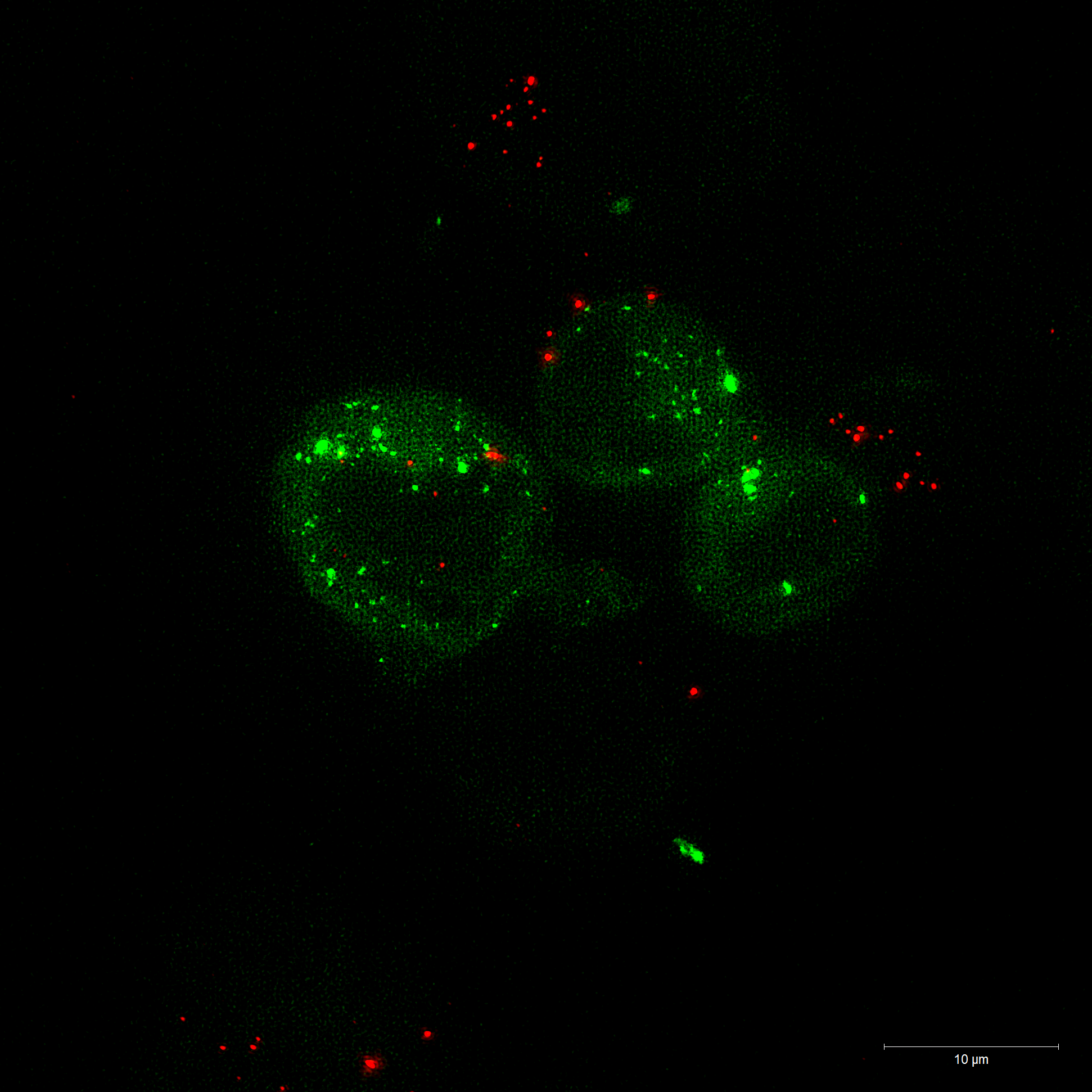

Supplement: Supplementary file 8 — Source Data Fig. 6 [file 44321_2023_14_MOESM8_ESM.zip › Figure 6/Fig 6D/JEV+MTP/Image 1_Out_ MTP.tif]

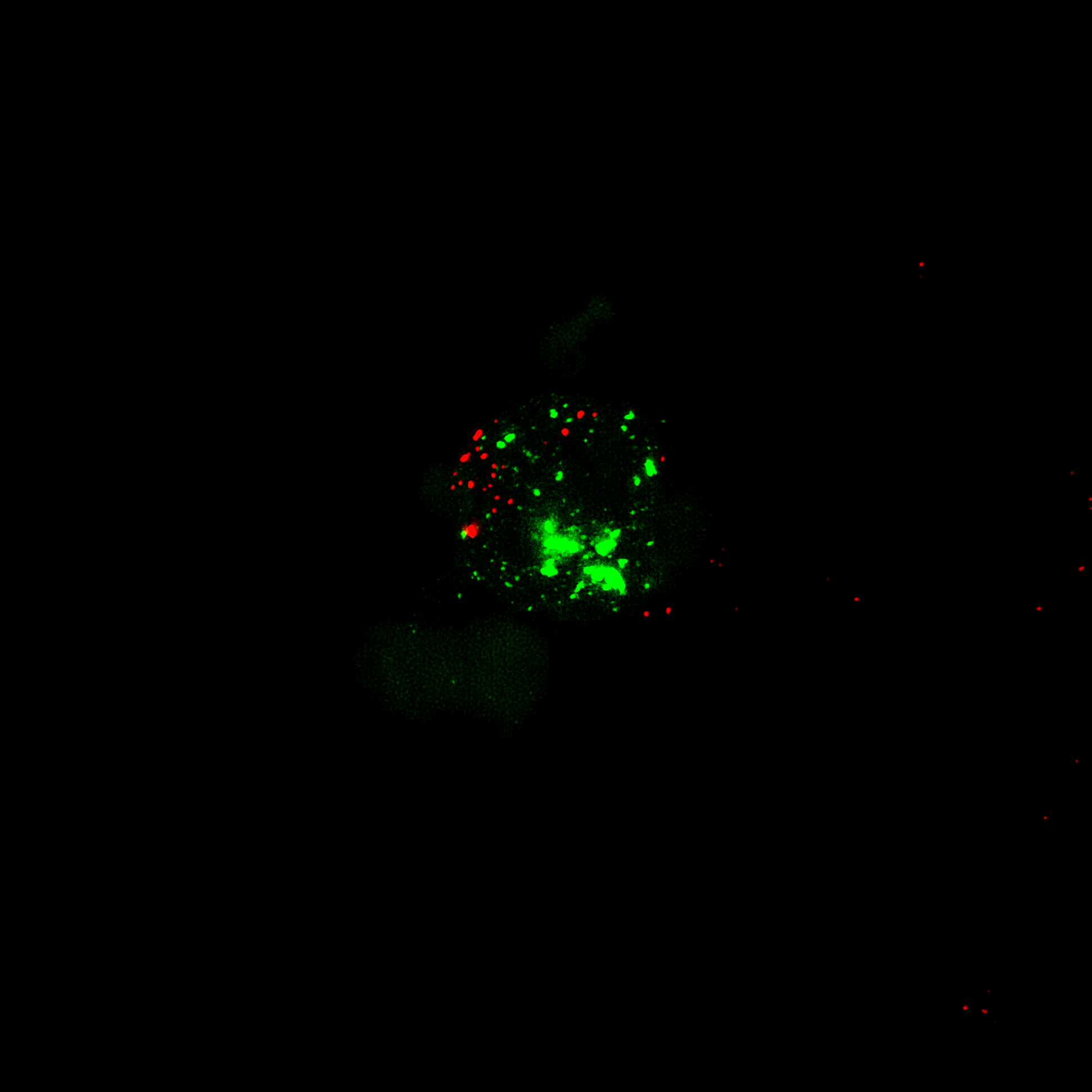

Supplement: Supplementary file 8 — Source Data Fig. 6 [file 44321_2023_14_MOESM8_ESM.zip › Figure 6/Fig 6D/JEV+MTP/Image 1_Out_Maximum intensity projection.tif]

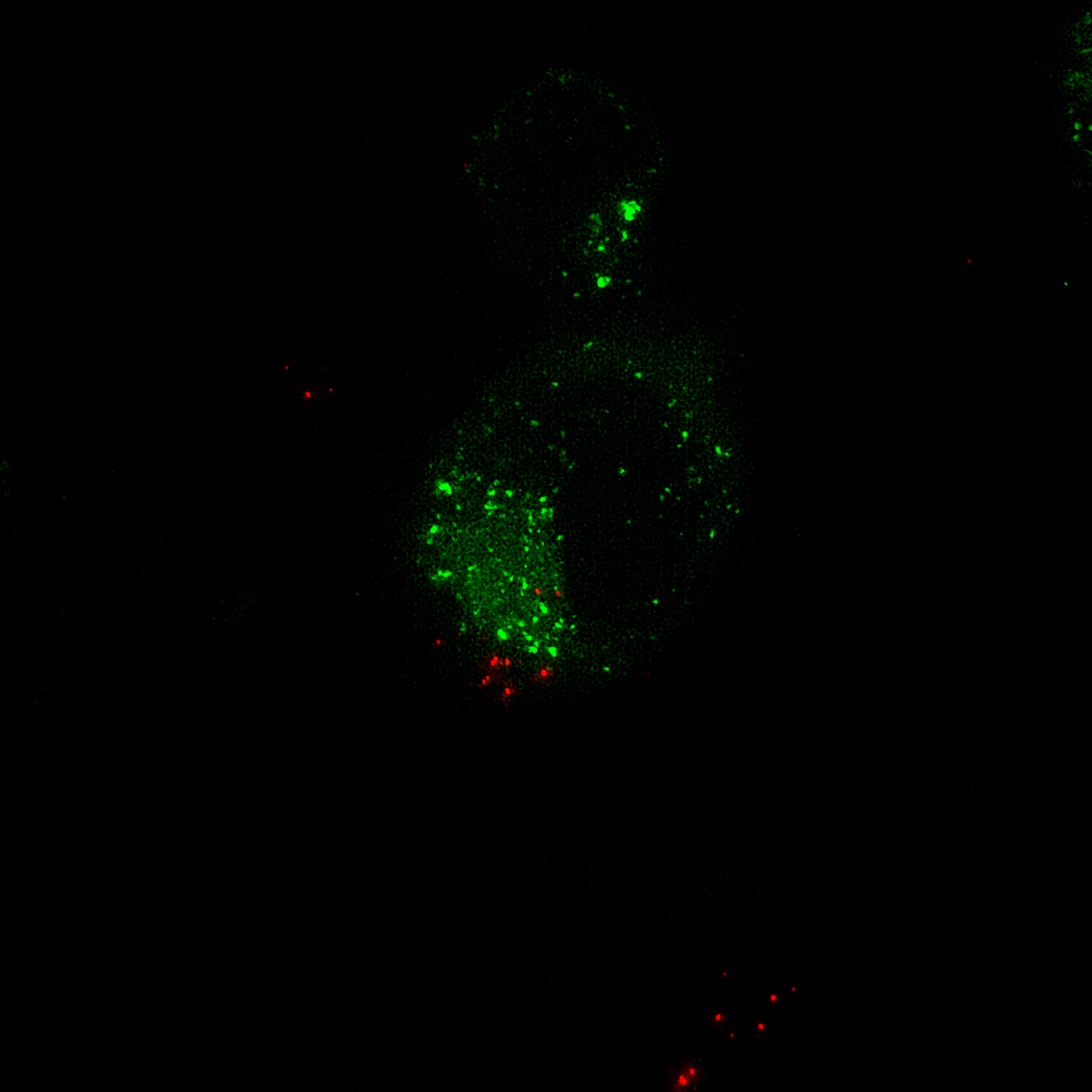

Supplement: Supplementary file 8 — Source Data Fig. 6 [file 44321_2023_14_MOESM8_ESM.zip › Figure 6/Fig 6D/JEV+MTP/Image 4_Out_Maximum intensity projection.tif]

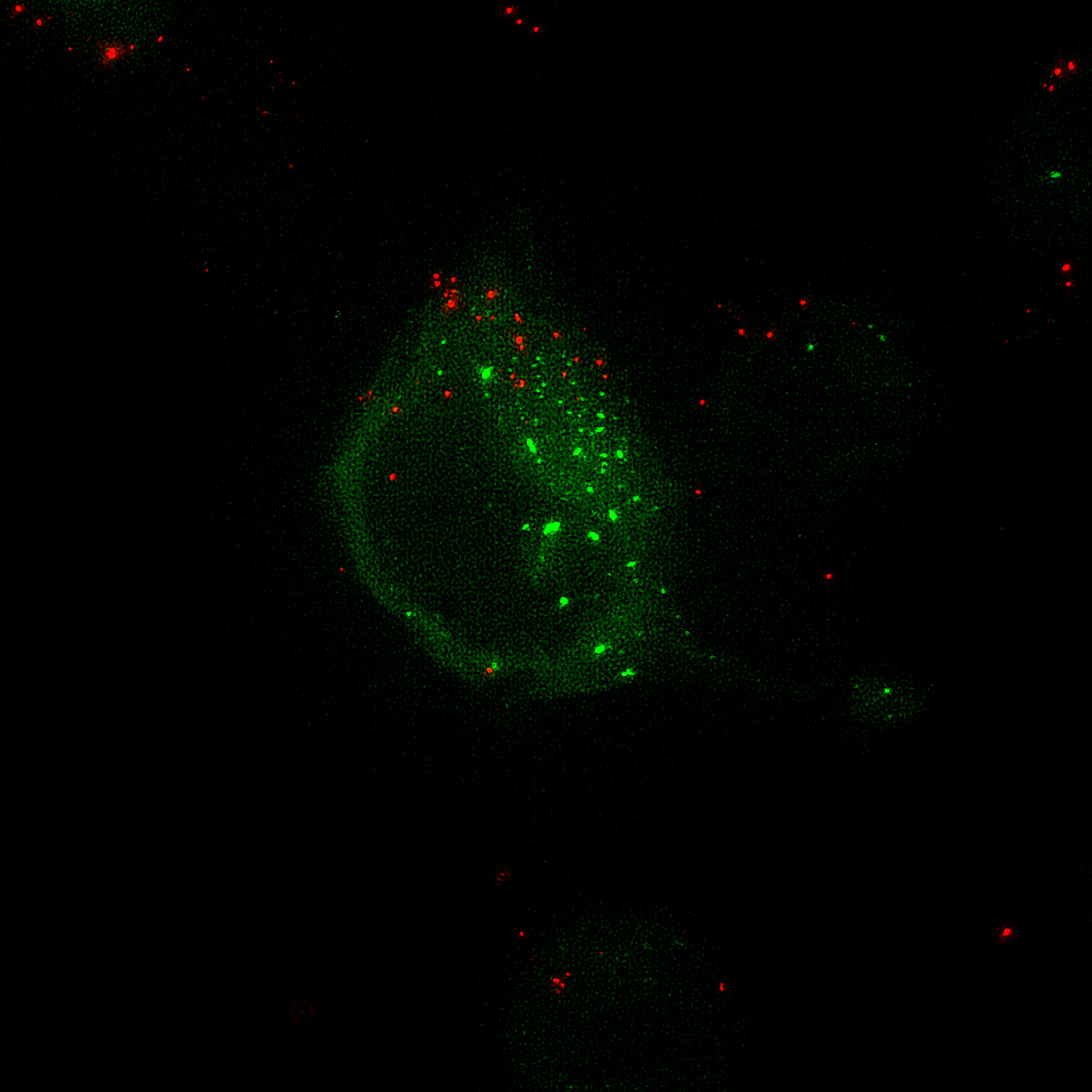

Supplement: Supplementary file 8 — Source Data Fig. 6 [file 44321_2023_14_MOESM8_ESM.zip › Figure 6/Fig 6D/JEV+MTP/Image 5_Out_Maximum intensity projection.tif]

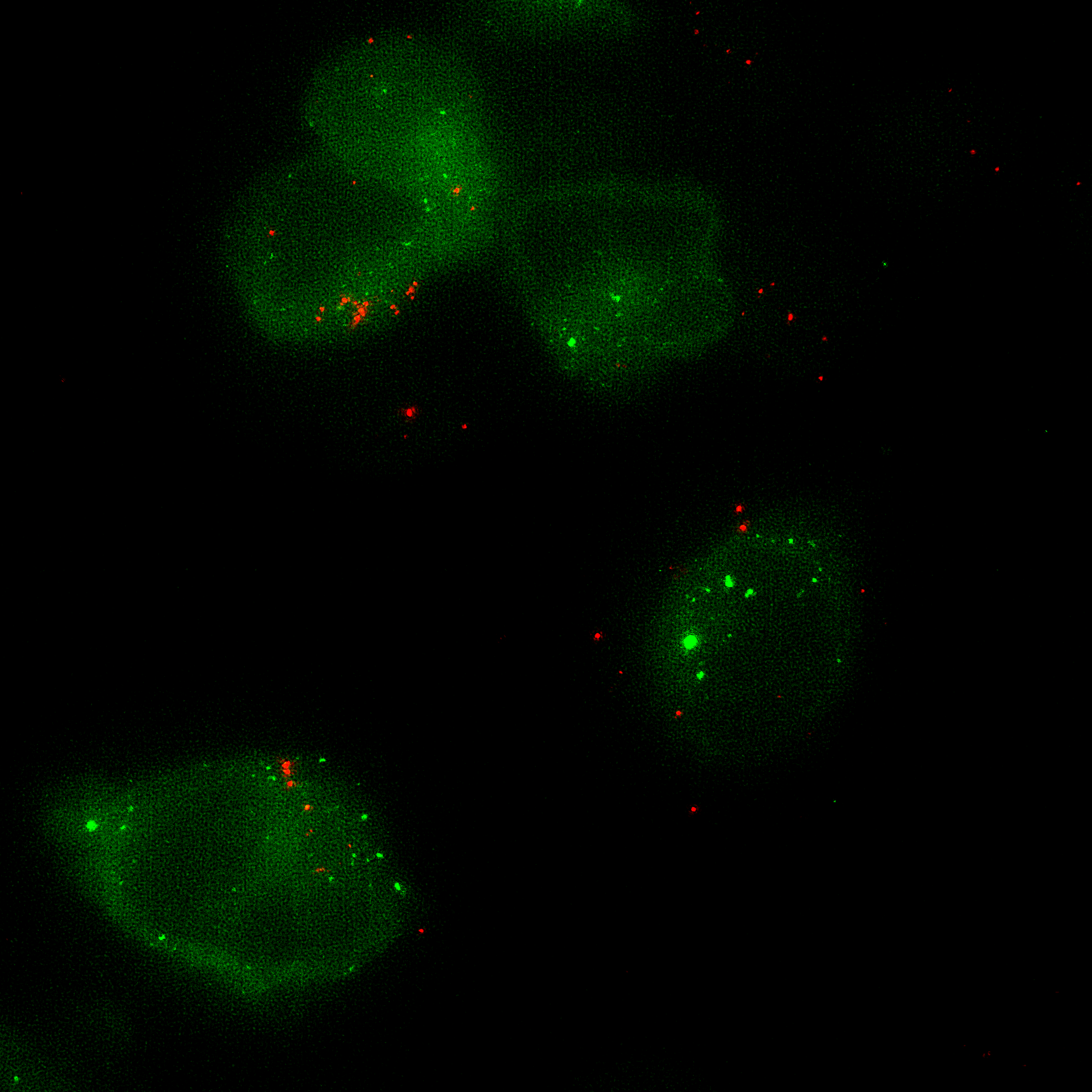

Supplement: Supplementary file 8 — Source Data Fig. 6 [file 44321_2023_14_MOESM8_ESM.zip › Figure 6/Fig 6D/JEV+MTP/Image 6_Out_Maximum intensity projection.tif]

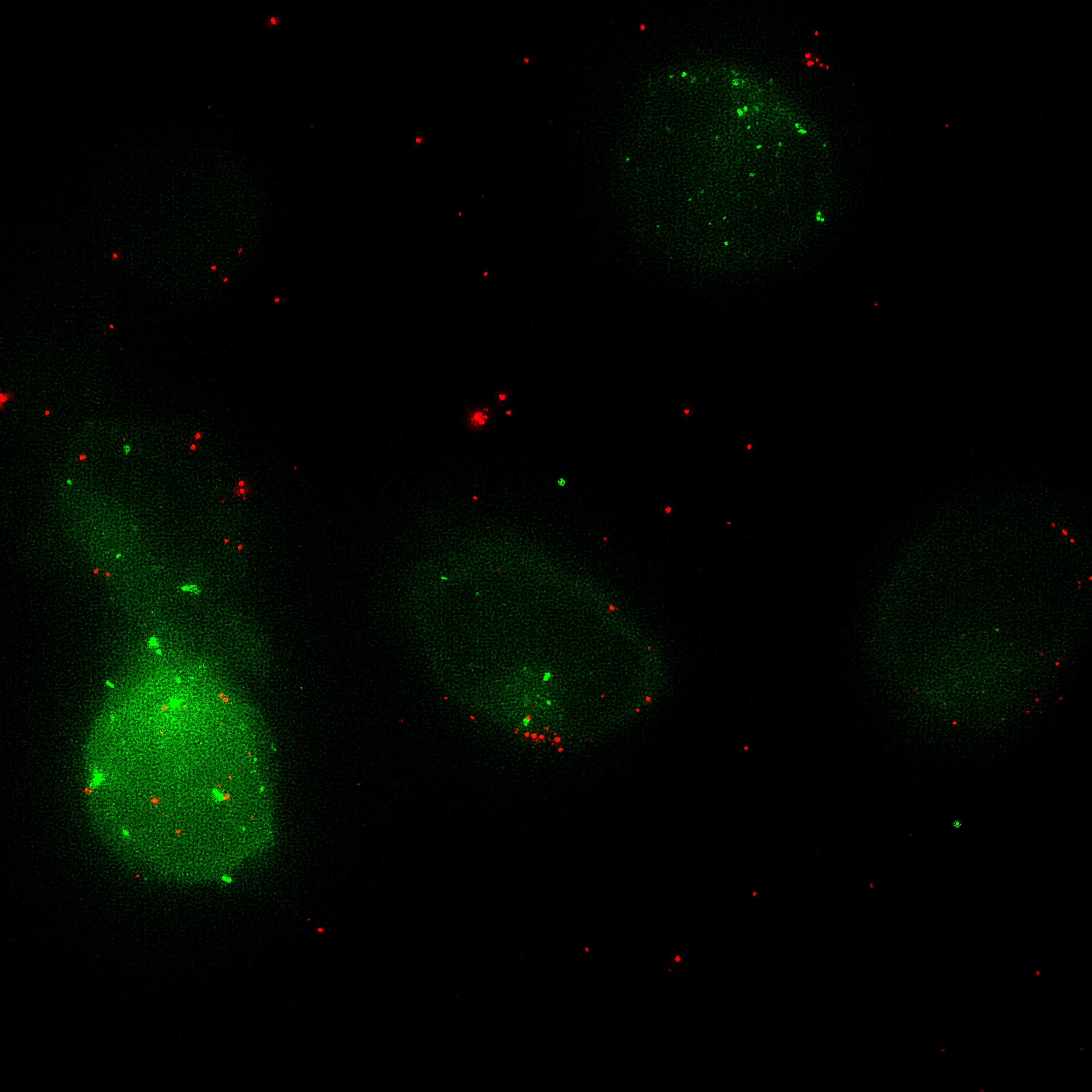

Supplement: Supplementary file 8 — Source Data Fig. 6 [file 44321_2023_14_MOESM8_ESM.zip › Figure 6/Fig 6D/JEV+MTP/Image 7_Out_Maximum intensity projection.tif]

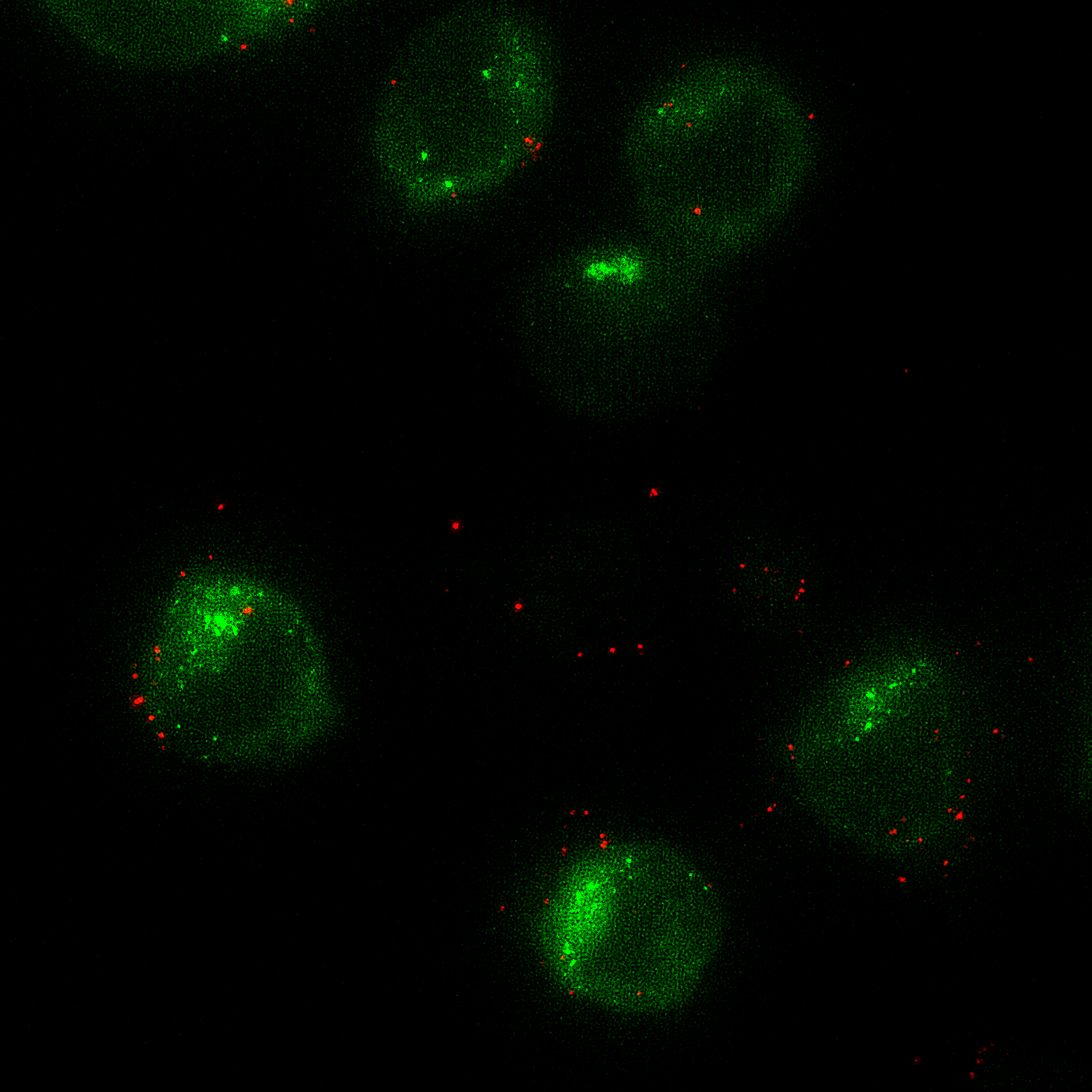

Supplement: Supplementary file 8 — Source Data Fig. 6 [file 44321_2023_14_MOESM8_ESM.zip › Figure 6/Fig 6D/JEV+MTP/Image 8_Out_Maximum intensity projection.tif]

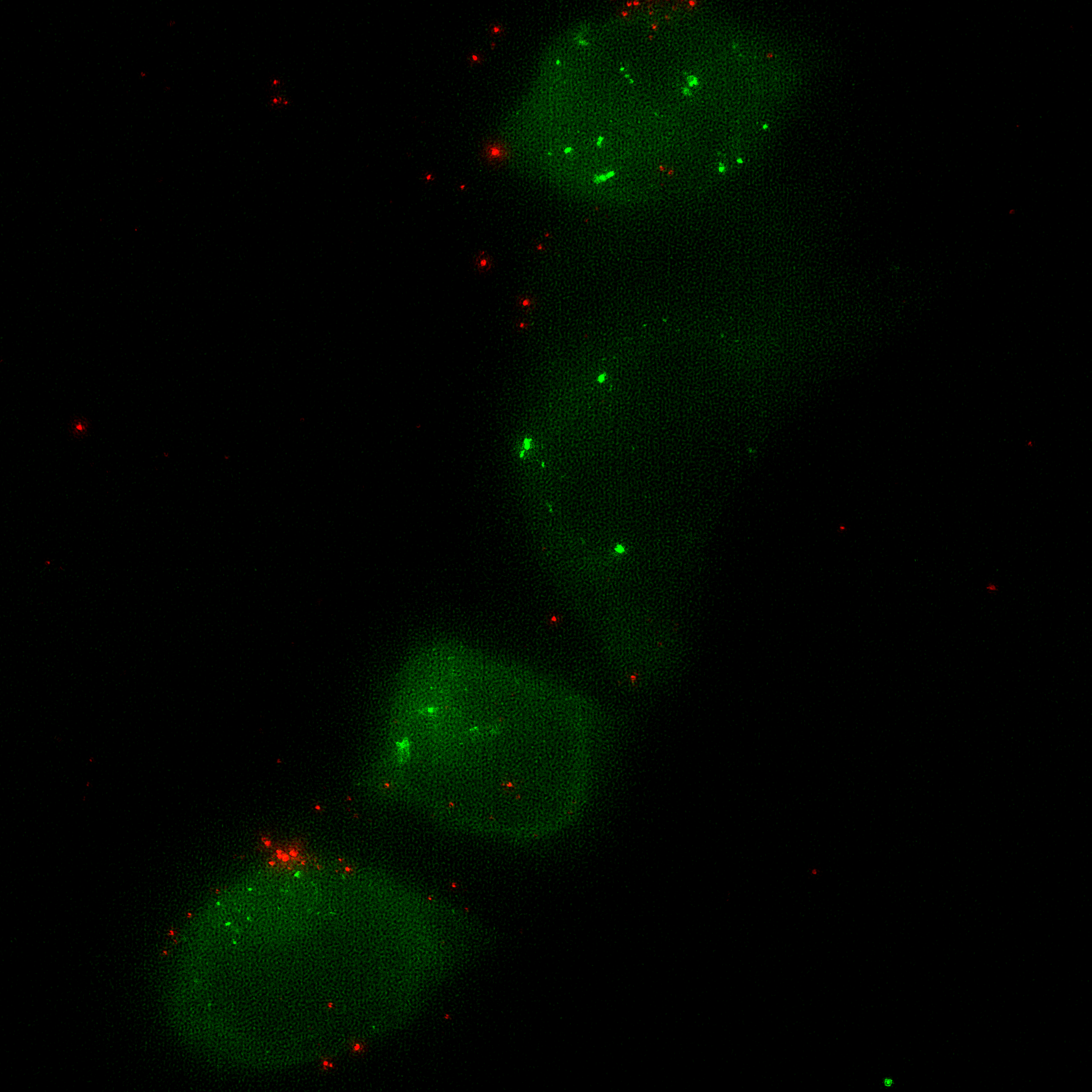

Supplement: Supplementary file 8 — Source Data Fig. 6 [file 44321_2023_14_MOESM8_ESM.zip › Figure 6/Fig 6D/JEV+MTP/Image 9_Out_Maximum intensity projection.tif]

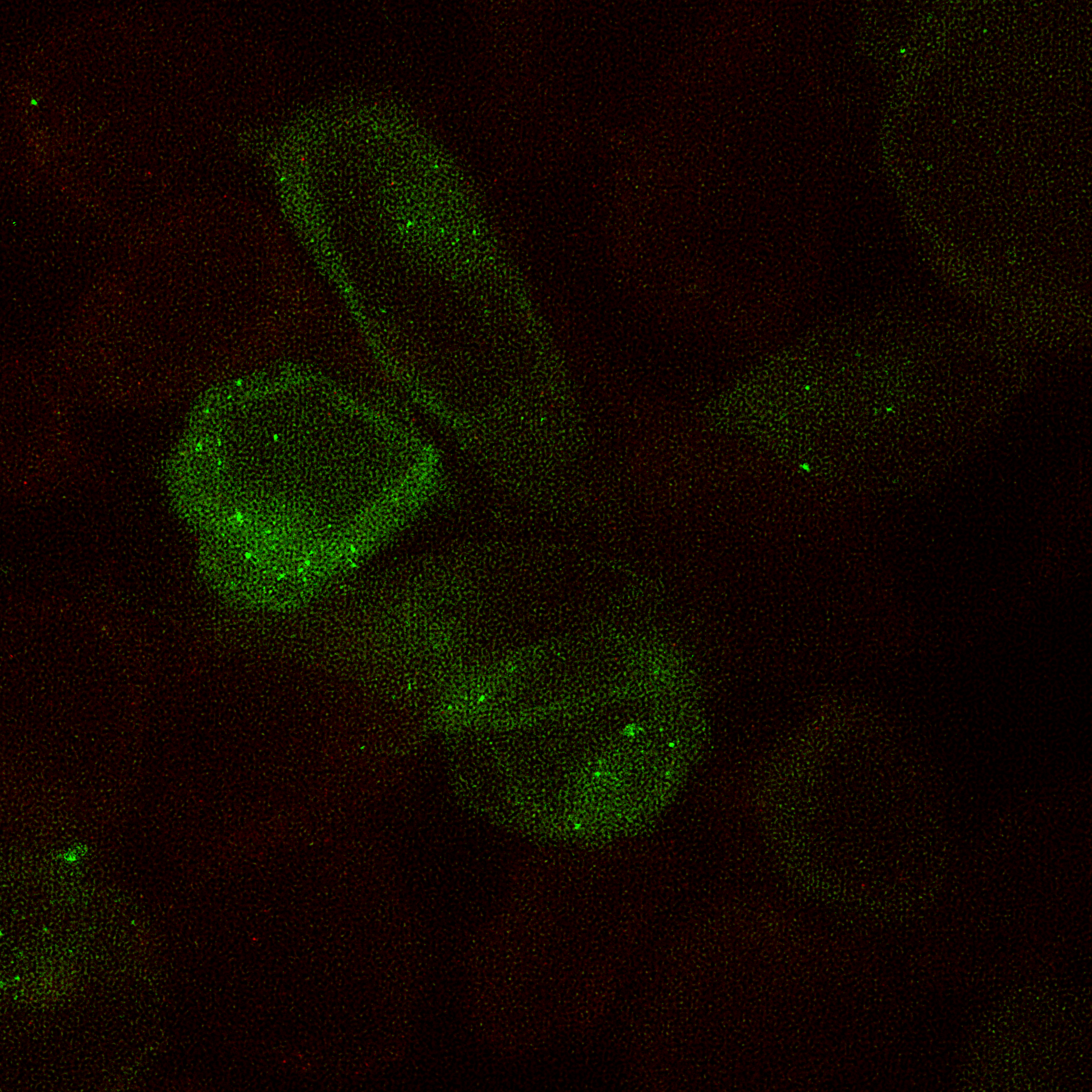

Supplement: Supplementary file 8 — Source Data Fig. 6 [file 44321_2023_14_MOESM8_ESM.zip › Figure 6/Fig 6D/Mock/Image 2_Out_mock.tif]

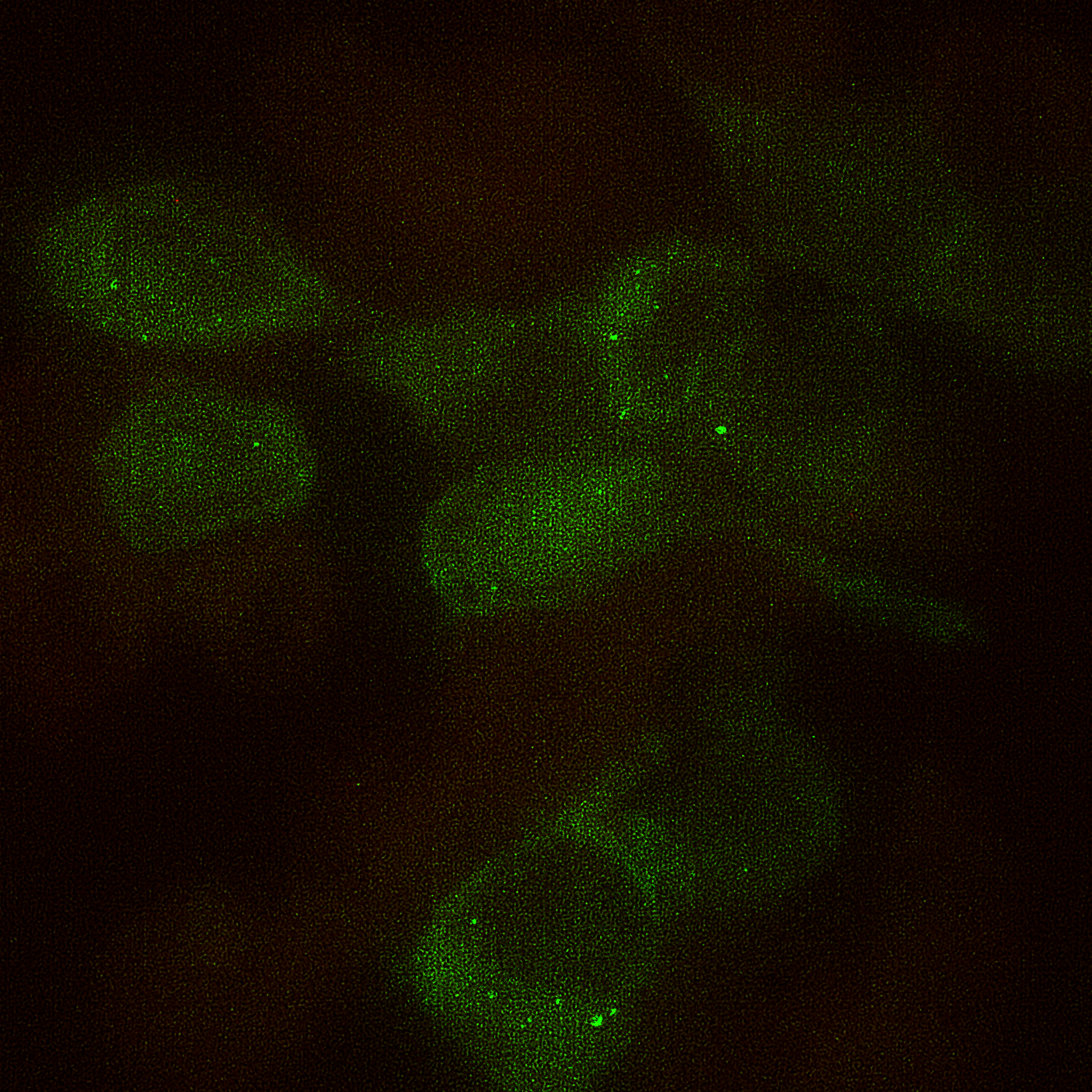

Supplement: Supplementary file 8 — Source Data Fig. 6 [file 44321_2023_14_MOESM8_ESM.zip › Figure 6/Fig 6D/Mock/Image 4_Out_Maximum intensity projection.tif]

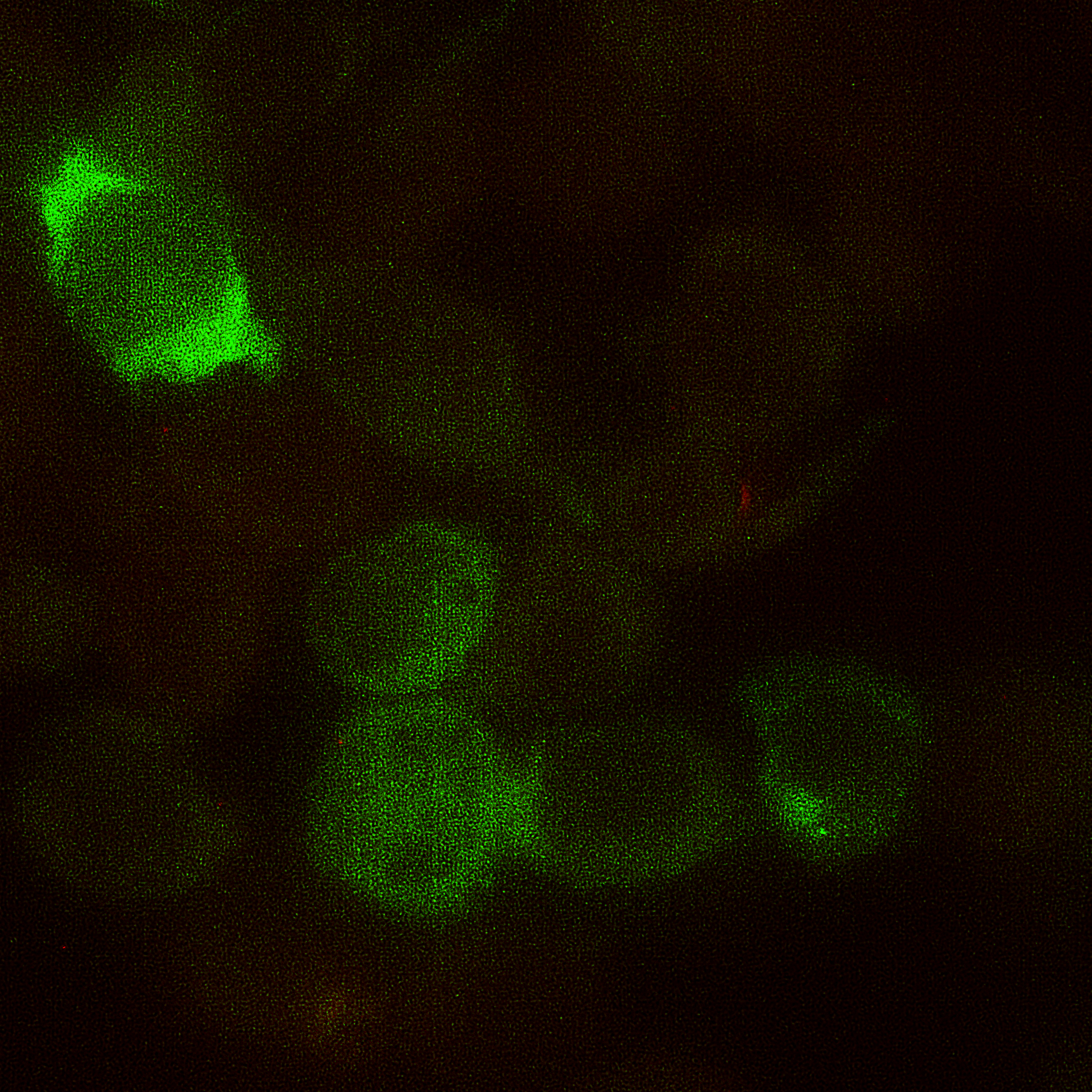

Supplement: Supplementary file 8 — Source Data Fig. 6 [file 44321_2023_14_MOESM8_ESM.zip › Figure 6/Fig 6D/Mock/Image 5_Out_Maximum intensity projection.tif]

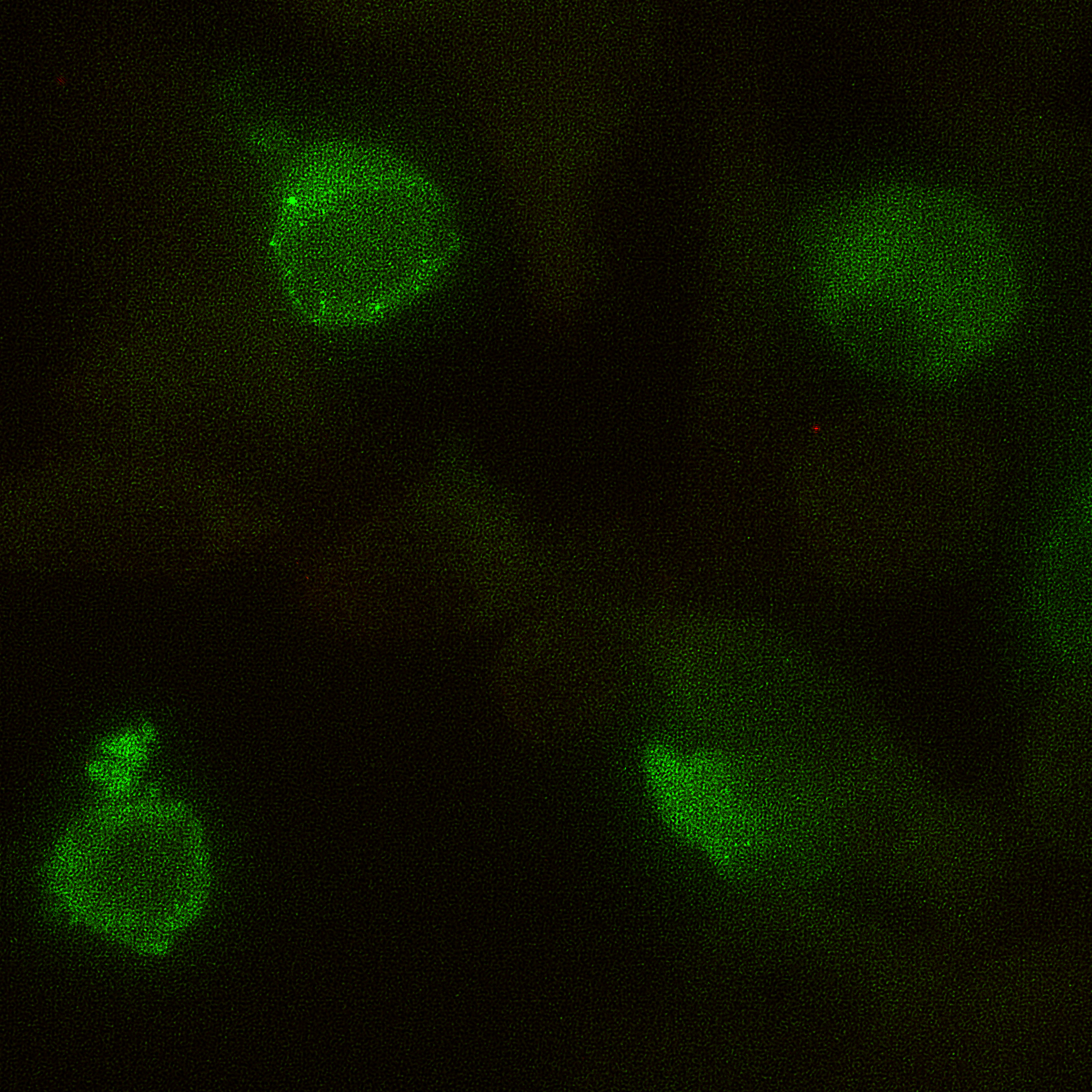

Supplement: Supplementary file 8 — Source Data Fig. 6 [file 44321_2023_14_MOESM8_ESM.zip › Figure 6/Fig 6D/Mock/Image 6_Out_Maximum intensity projection.tif]

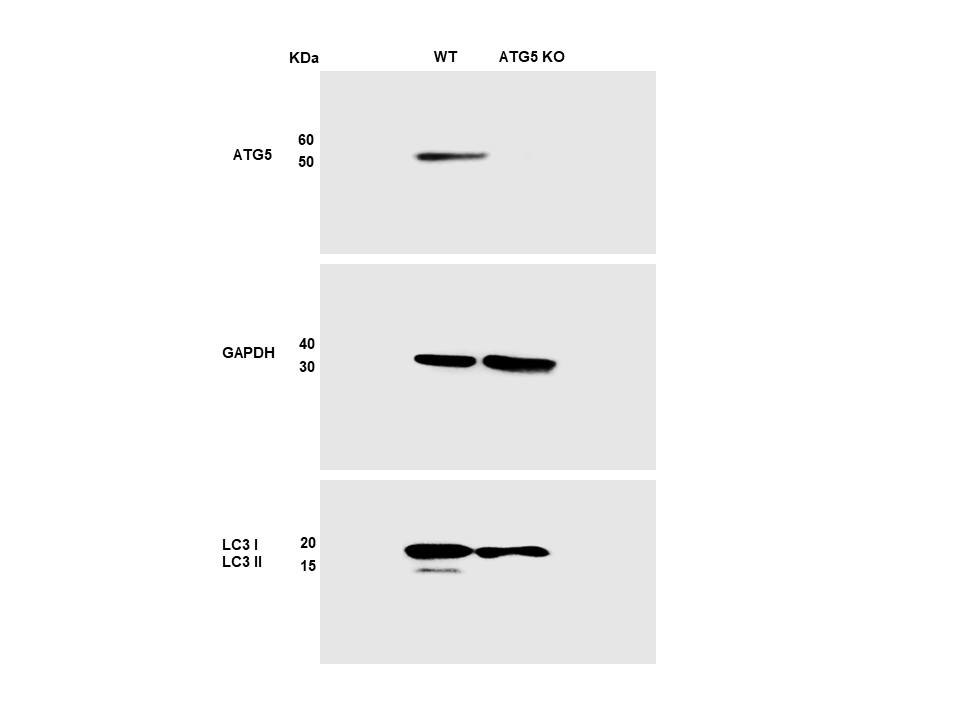

Supplement: Supplementary file 9 — Source Data Fig. 7 [file 44321_2023_14_MOESM9_ESM.zip › Figure 7/Fig 7A/ATG5 KO.tif]

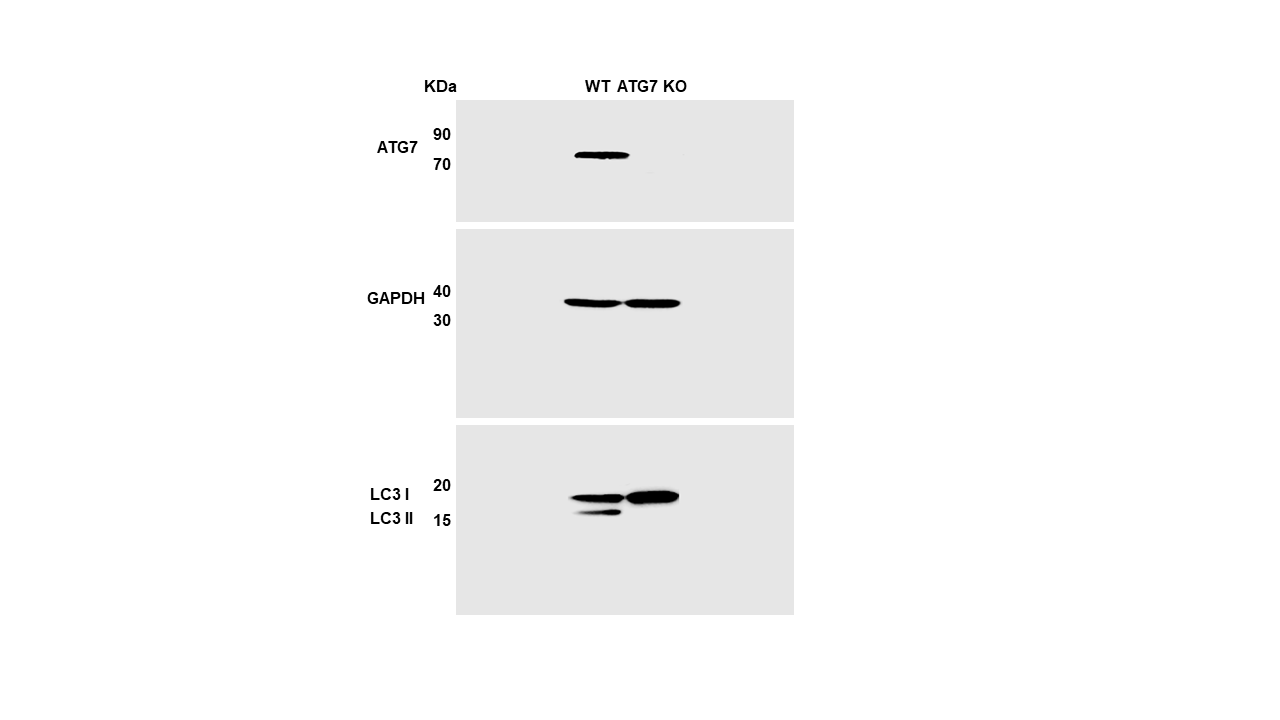

Supplement: Supplementary file 9 — Source Data Fig. 7 [file 44321_2023_14_MOESM9_ESM.zip › Figure 7/Fig 7D/ATG7KO N2a.tif]

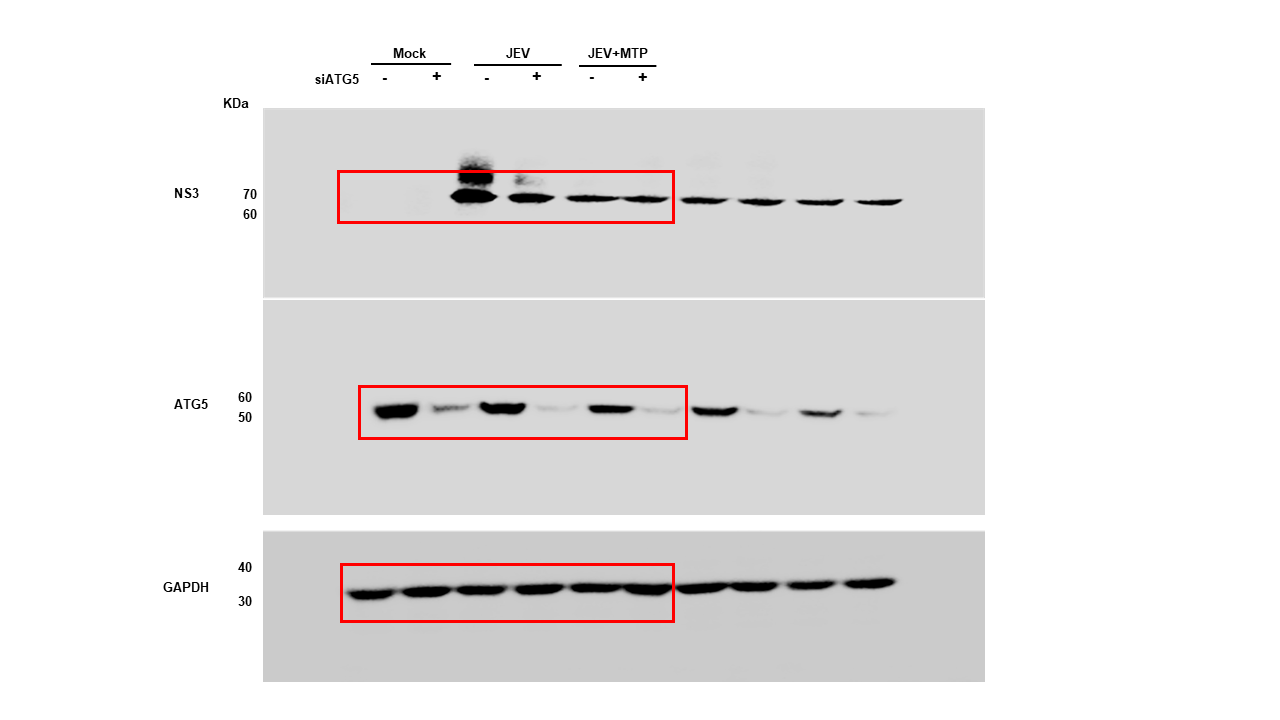

Supplement: Supplementary file 10 — Source Data Fig. 8 [file 44321_2023_14_MOESM10_ESM.zip › Figure 8/Fig 8A/atg5KD.tif]

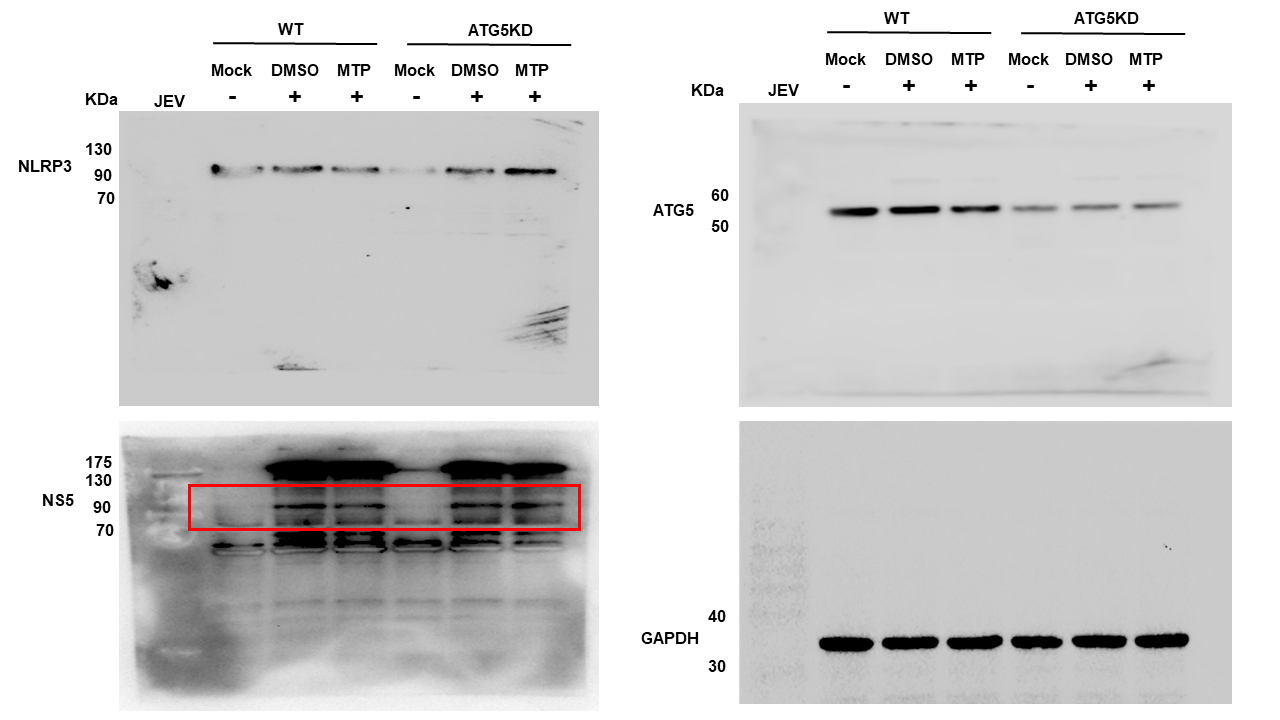

Supplement: Supplementary file 10 — Source Data Fig. 8 [file 44321_2023_14_MOESM10_ESM.zip › Figure 8/Fig 8C/Exp 1.tif]

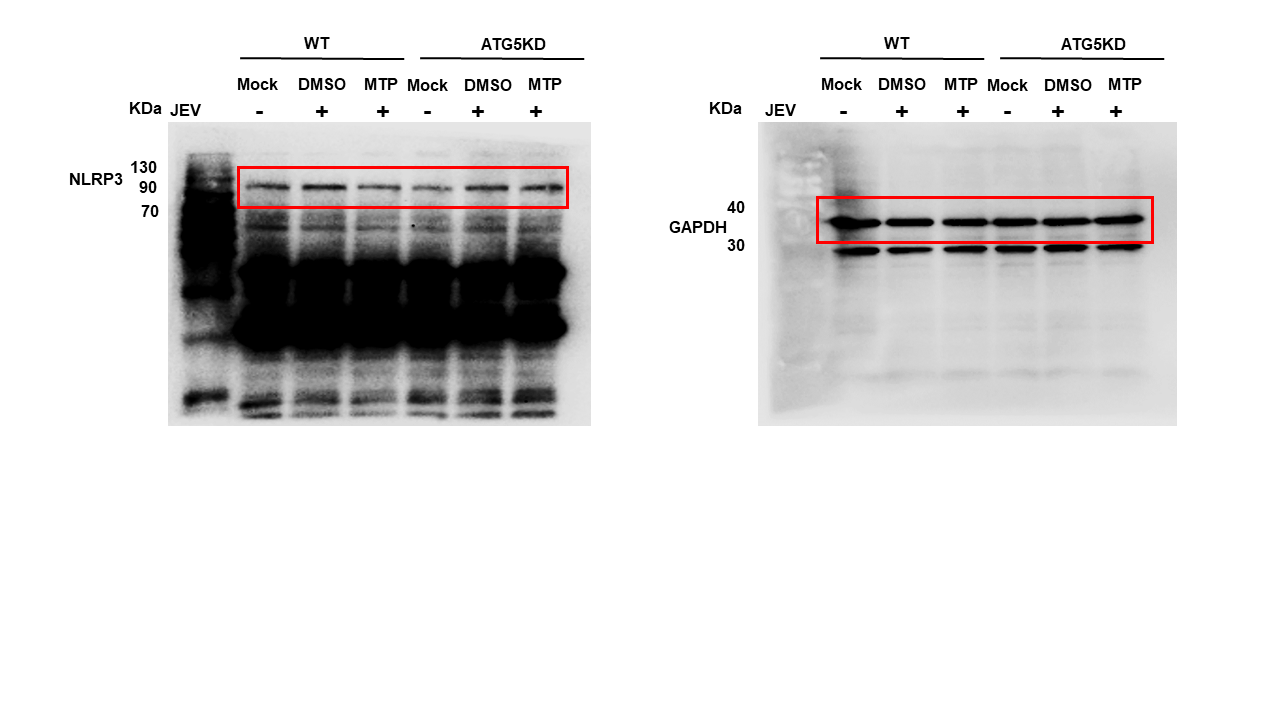

Supplement: Supplementary file 10 — Source Data Fig. 8 [file 44321_2023_14_MOESM10_ESM.zip › Figure 8/Fig 8C/Exp 2.tif]

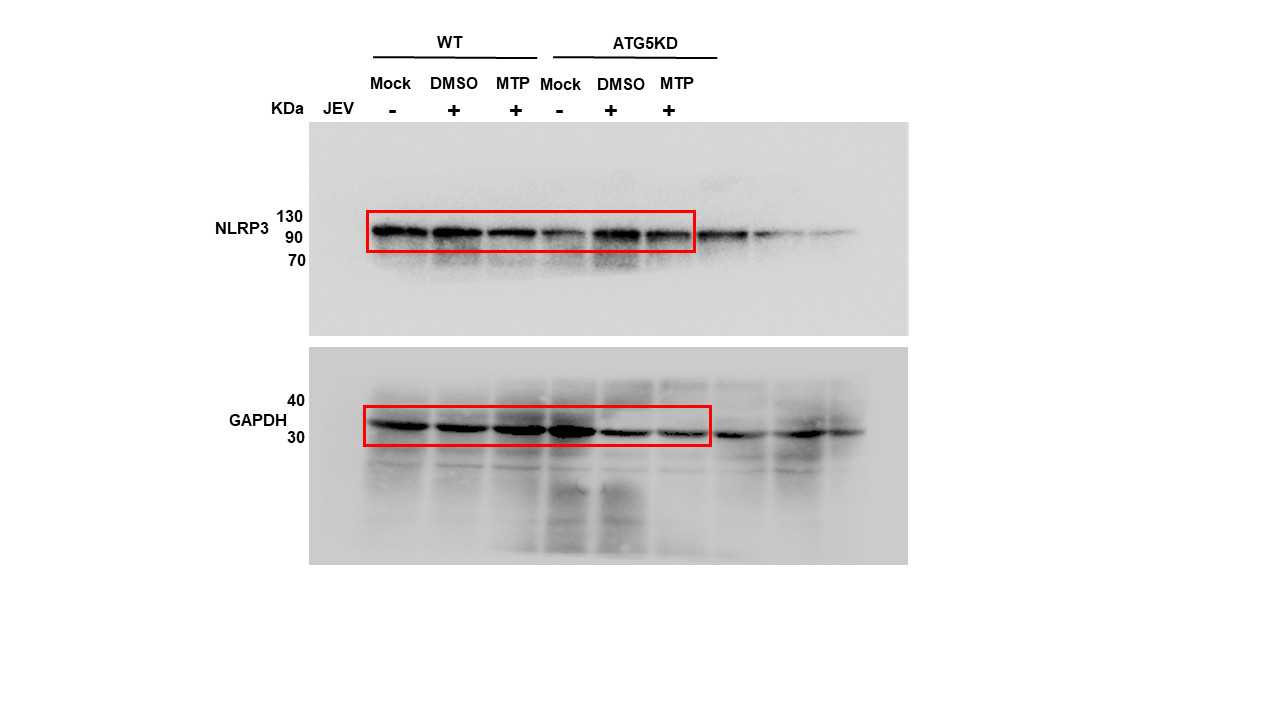

Supplement: Supplementary file 10 — Source Data Fig. 8 [file 44321_2023_14_MOESM10_ESM.zip › Figure 8/Fig 8C/Exp 3.tif]

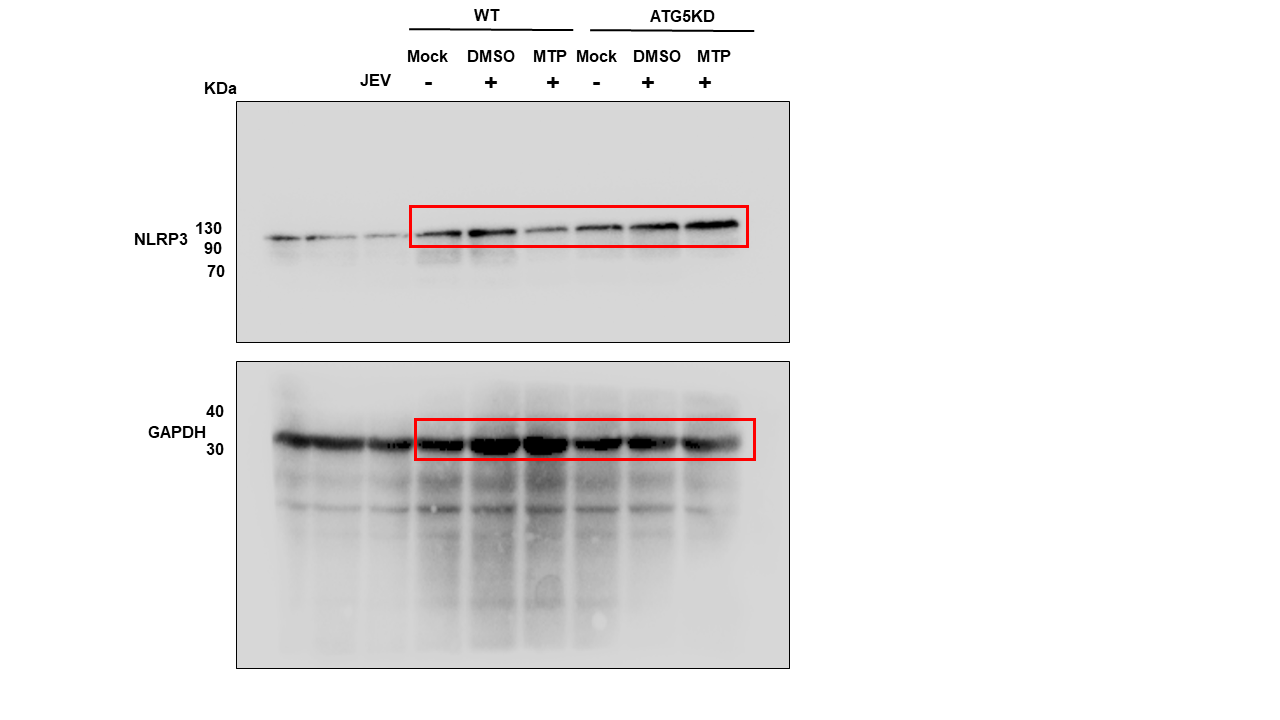

Supplement: Supplementary file 10 — Source Data Fig. 8 [file 44321_2023_14_MOESM10_ESM.zip › Figure 8/Fig 8C/Exp 4.tif]

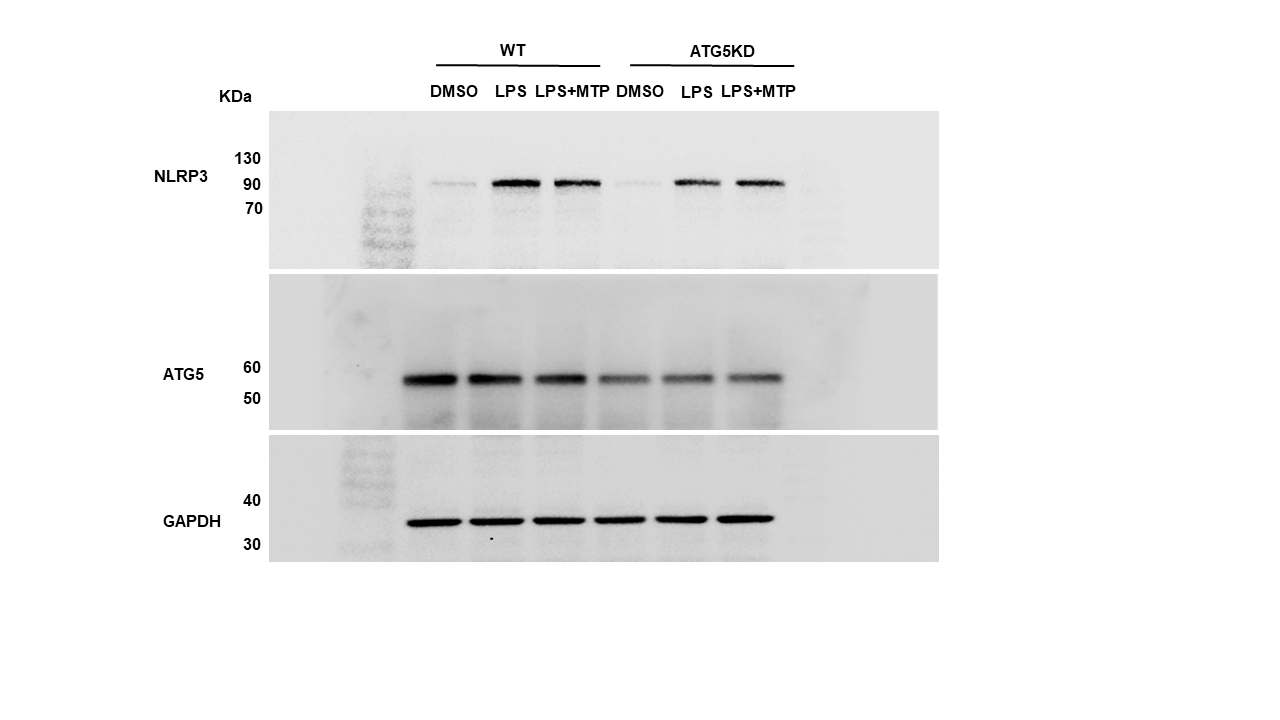

Supplement: Supplementary file 10 — Source Data Fig. 8 [file 44321_2023_14_MOESM10_ESM.zip › Figure 8/Fig 8E/Exp 1.tif]

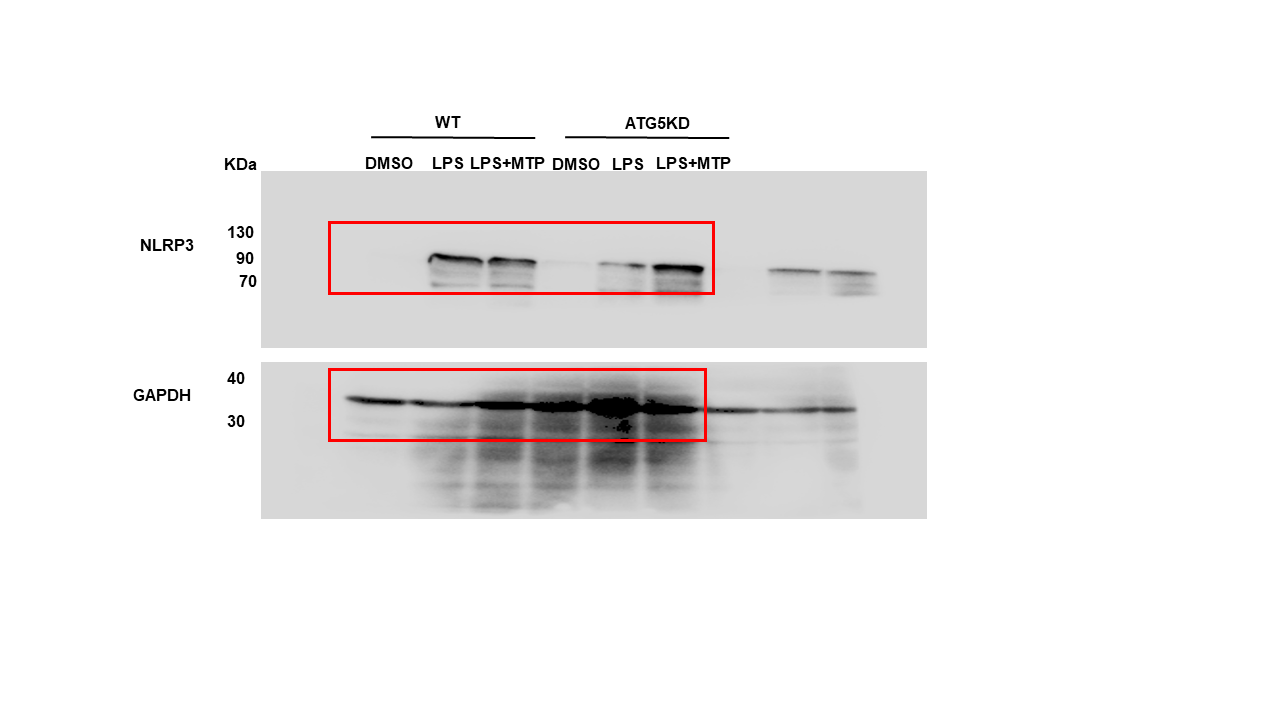

Supplement: Supplementary file 10 — Source Data Fig. 8 [file 44321_2023_14_MOESM10_ESM.zip › Figure 8/Fig 8E/Exp 2.tif]

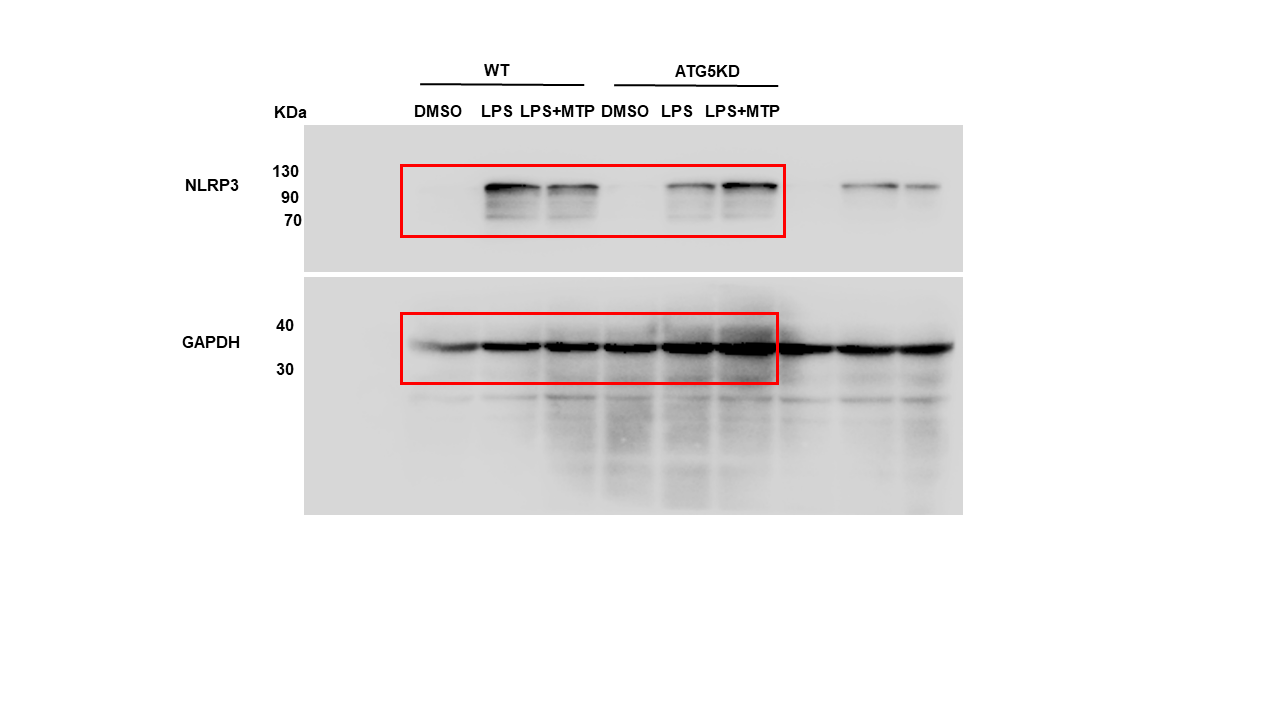

Supplement: Supplementary file 10 — Source Data Fig. 8 [file 44321_2023_14_MOESM10_ESM.zip › Figure 8/Fig 8E/Exp 3.tif]
