# Supplementary material for: Methotrimeprazine is a neuroprotective antiviral in JEV infection via adaptive ER stress and autophagy
Source: EMBO Mol Med. 2024 Jan 2;16(1):185–217. doi: 10.1038/s44321-023-00014-w (PMC10897192; doi:10.1038/s44321-023-00014-w)
Supplement: Supplementary file 12 — Expanded View Figures [file 44321_2023_14_MOESM12_ESM.pdf]

Expanded View Figures

Figure EV1. MTP inhibits the secretion of proinflammatory cytokines from JEV infected/LPS-stimulated astrocytes.

(**A**) Primary astrocytes were isolated from P2 pups, and purity was confirmed through immunofluorescence staining with GFAP antibody. Scale bar, 10 μm. (**B**) Primary astrocytes were treated with DMSO/MTP (10 μM)/LPS (1 μg/ml)/LPS + MTP for 24 h. Percentage cell viability was measured by MTT assay ($n = 3$). (**C**,**D**) Primary astrocytes were mock/JEV (MOI 1) infected for 1 h, followed by treatment with either DMSO or MTP (10 μM) till 24 h. (**C**) Viral RNA levels were quantified using qRT-PCR. Graph shows the relative expression levels of JEV RNA normalized to DMSO-treated control. Data is plotted from two independent experiments ($n = 6$). (**D**) Culture supernatant was used to determine virus titers using plaque assays. Data represents values obtained from two independent experiments ($n = 6$). (**E**,**F**) Primary astrocytes were infected with JEV (MOI 1) for 1 h then treated with DMSO/MTP (10 μM) till 24 h (**E**), or were treated with DMSO (mock)/LPS (1 μg/ml)/LPS + MTP for 24 h (**F**). Culture supernatants were collected and cytokine levels were quantitated by CBA using flow cytometry. Data were analyzed with LEGENDplexTM Multiplex assay software. Data shows values from one representative experiment ($n = 3$). Similar trends were seen in two independent experiments. Data information: All data expressed as means ± SD, statistical significance was determined using unpaired Student t-test. *$P < 0.05$; **$P < 0.01$; ***$P < 0.001$; ****$P < 0.0001$.

© The Author(s)

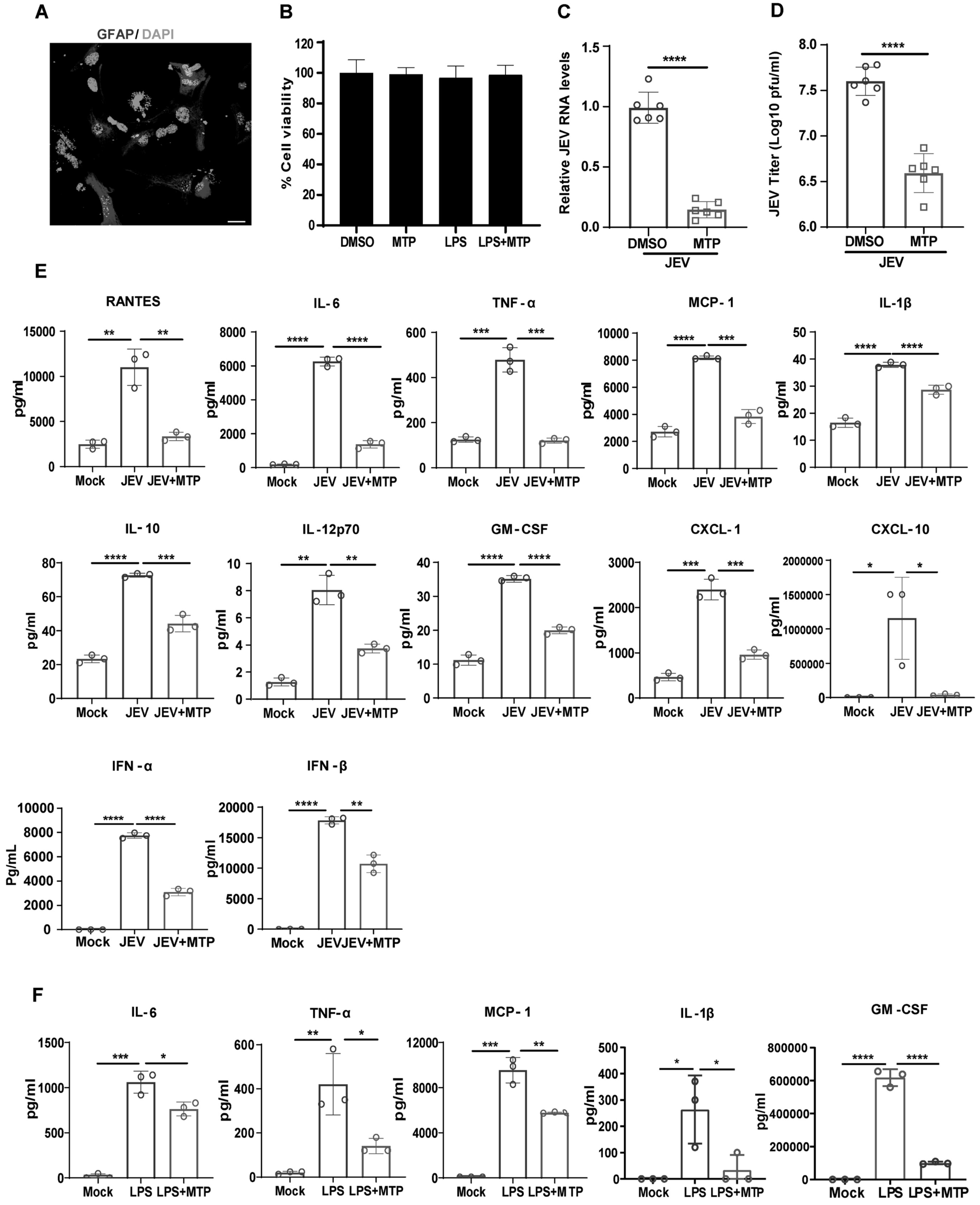
A
GFAP/ DAPI
B
% Cell viability
DMSO MTP LPS LPS+MTP
C
Relative JEV RNA levels
DMSO MTP
JEV
D
JEV Titer (Log10 pfu/ml)
DMSO MTP
JEV
E
RANTES
IL-6
TNF-α
MCP-1
IL-1β
IL-10
IL-12p70
GM-CSF
CXCL-1
CXCL-10
IFN-α
IFN-β
pg/ml
Pg/mL
Mock JEV JEV+MTP
F
IL-6
TNF-α
MCP-1
IL-1β
GM-CSF
pg/ml
Mock LPS LPS+MTP

 © The Author(s)

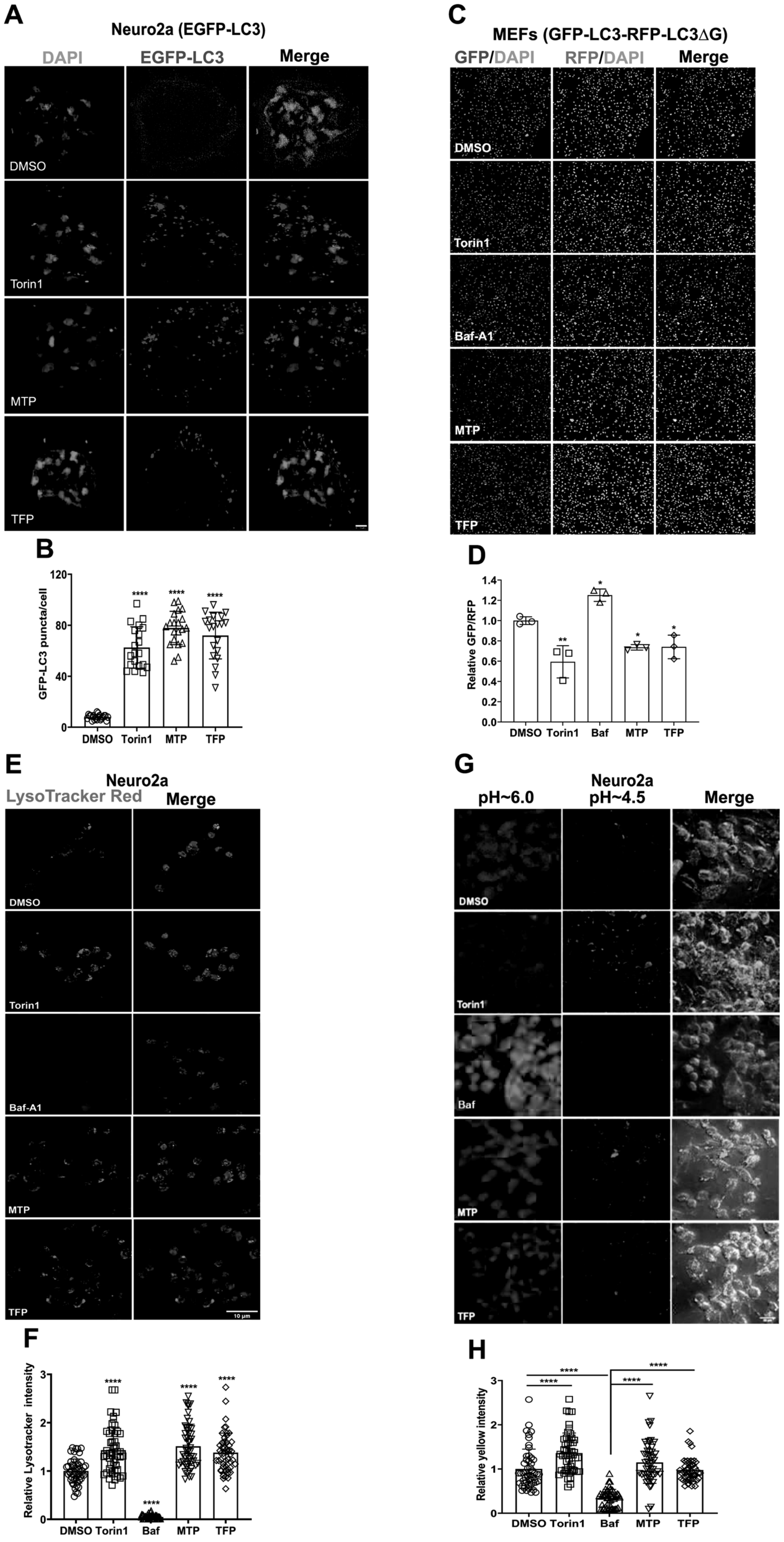
A
Neuro2a (EGFP-LC3)
DAPI
EGFP-LC3
Merge
DMSO
Torin1
MTP
TFP
C
MEFs (GFP-LC3-RFP-LC3ΔG)
GFP/DAPI
RFP/DAPI
Merge
DMSO
Torin1
Baf-A1
MTP
TFP
B
GFP-LC3 puncta/cell
DMSO Torin1 MTP TFP
D
Relative GFP/RFP
DMSO Torin1 Baf MTP TFP
E
Neuro2a
LysoTracker Red
Merge
DMSO
Torin1
Baf-A1
MTP
TFP
10 μm
G
Neuro2a
pH~6.0
pH~4.5
Merge
DMSO
Torin1
Baf
MTP
TFP
F
Relative Lysotracker intensity
DMSO Torin1 Baf MTP TFP
H
Relative yellow intensity
DMSO Torin1 Baf MTP TFP

◀ **Figure EV2. Phenothiazines induce functional autophagy flux and do not alter lysosomal pH.**

(**A**,**B**) EGFP-LC3 expressing stable Neuro2a cells were treated with DMSO/Torin1 (1 μM)/MTP (10 μM)/TFP (10 μM) for 6 h. (**A**) Representative SIM images are shown. Scale bar, 10 μm. (**B**) Bar-graph shows quantitation of EGFP-LC3 puncta per cell. Data is acquired from 20 cells across two independent coverslips. (**C**,**D**) GFP-LC3-RFP-LC3ΔG expressing stable MEFs were treated with DMSO (control)/Torin1 (1 μM)/BafA1 (100 nM) or MTP/TFP (10 μM) for 6 h. (**C**) Images were visualized by high-content imaging system. Scale bar, 100 μm. (**D**) Graph showing GFP/RFP ratios ($n = 3$). (**E–H**) Neuro2a cells grown on glass coverslips were treated with indicated drugs as described above for 6 h, followed by incubation with 10 μM LysoTracker Red for 40 min (**E**,**F**) or 10 μM LysoSensor Yellow-Blue for 5 min (**G**,**H**). Representative confocal images are shown. Scale bar, 10 μm (**E**); 20 μm (**G**). LysoTracker Red (**F**) and LysoSensor Yellow-Blue (yellow) (**H**) fluorescence intensities were calculated from 50 cells across two independent coverslips using ImageJ (Fiji). Data information: All data are normalized to DMSO control and expressed as means ± SD, one-way ANOVA test followed by Dunnett test was used for statistical significance. *$P < 0.05$; **$P < 0.01$; ****$P < 0.0001$.

 © The Author(s)

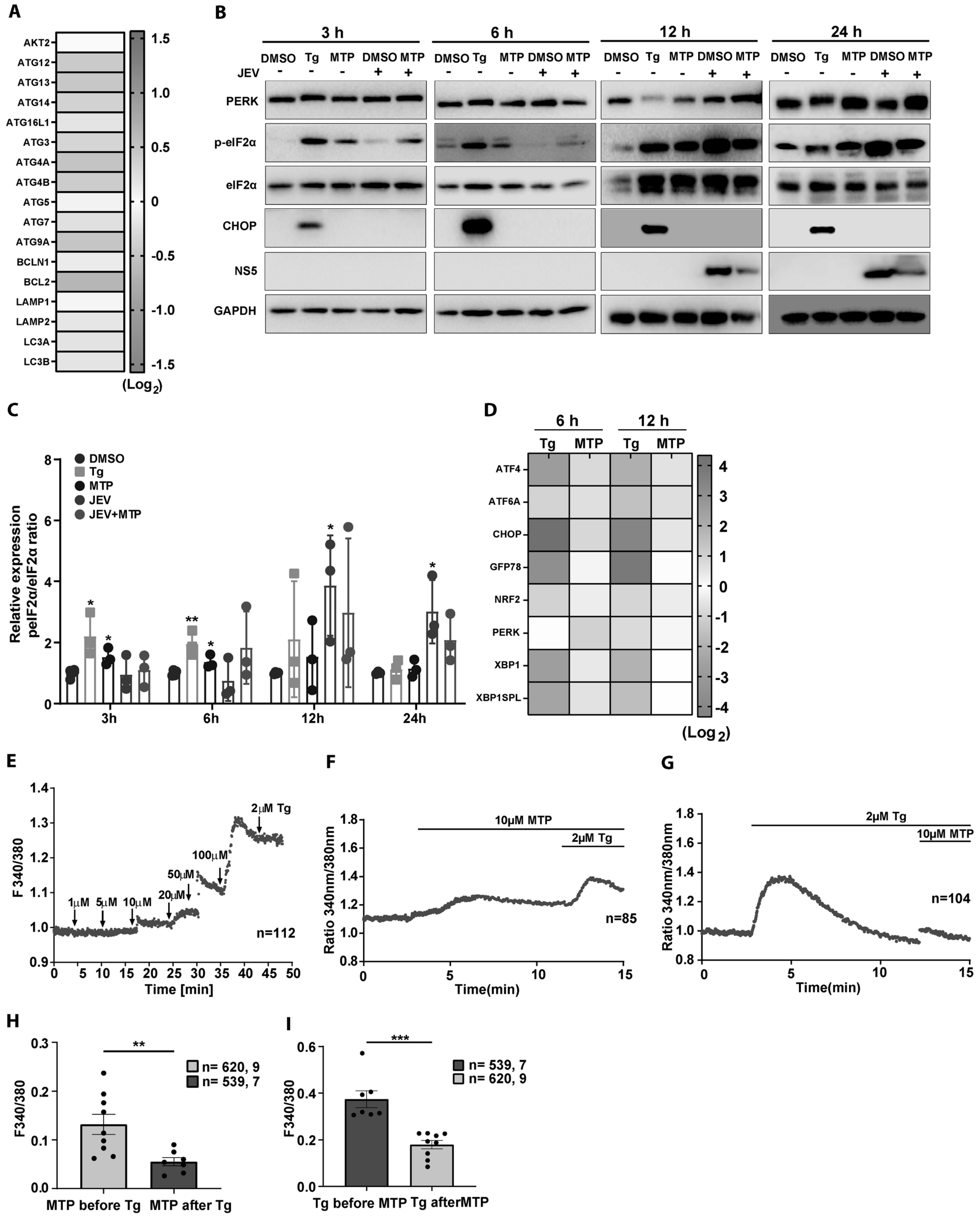
A
AKT2
ATG12
ATG13
ATG14
ATG16L1
ATG3
ATG4A
ATG4B
ATG5
ATG7
ATG9A
BCLN1
BCL2
LAMP1
LAMP2
LC3A
LC3B
(Log2)
B
3 h
6 h
12 h
24 h
DMSO Tg MTP DMSO MTP
JEV
PERK
p-eIF2α
eIF2α
CHOP
NS5
GAPDH
C
DMSO
Tg
MTP
JEV
JEV+MTP
Relative expression peIF2α/eIF2α ratio
3h
6h
12h
24h
D
6 h
12 h
Tg MTP Tg MTP
ATF4
ATF6A
CHOP
GFP78
NRF2
PERK
XBP1
XBP1SPL
(Log2)
E
F 340/380
1μM 5μM 10μM 20μM 50μM 100μM 2μM Tg
n=112
Time [min]
F
10μM MTP
2μM Tg
Ratio 340nm/380nm
n=85
Time(min)
G
2μM Tg
10μM MTP
Ratio 340nm/380nm
n=104
Time(min)
H
F340/380
n= 620, 9
n= 539, 7
MTP before Tg
MTP after Tg
I
F340/380
n= 539, 7
n= 620, 9
Tg before MTP
Tg afterMTP

Figure EV3. MTP activates adaptive ER stress and dysregulates ER calcium homeostasis.

(**A**) MEFs were treated with DMSO/MTP (10 μM) for 6 h and mRNA levels of autophagy genes were determined by qRT-PCR. Heatmap shows relative gene expression level after normalization to DMSO-treated control ($n = 3$). (**B**,**C**) MEFs were infected with JEV (MOI 1), at 1 hpi cells were treated with DMSO/Tg (1 μM)/MTP (10 μM) for the indicated time points. (**B**) Protein lysates were analyzed by immunoblotting using PERK, p-eIF2α, eIF2α, CHOP, NS5 (infection control) and GAPDH (loading control) antibodies. (**C**) Bar-graph shows relative expression of p-eIF2α/eIF2α normalized to DMSO control from three independent experiments, unpaired Student t-test. (**D**) MEFs were treated with DMSO/Tg (1 μM)/MTP (10 μM) for the indicated time points. mRNA levels of ER stress markers and chaperones were quantified using qRT-PCR. Heatmap depicts relative gene expression normalized to DMSO control, represented as mean ($n = 3$). (**E**) Representative Ca^{2+} imaging trace of MTP dose–response assay, where "$n = 112$" denotes the number of cells in that particular trace. Cells were stimulated with increasing doses of MTP- 1 μM, 5 μM, 10 μM, 20 μM, 50 μM and 100 μM followed by addition of 2 μM thapsigargin (Tg) in Ca^{2+}-free buffer. (**F**) Representative Ca^{2+} imaging trace of experiments where cells were stimulated with 10 μM MTP in absence of extracellular Ca^{2+} followed by addition of 2 μM Tg. Here, "$n = 85$" denotes the number of cells in that particular trace. (**G**) Representative Ca^{2+} imaging trace of experiments where cells were stimulated first with 2 μM Tg to deplete ER Ca^{2+} stores, followed by addition of 10 μM MTP in absence of extracellular Ca^{2+}. Here, "$n = 104$" denotes the number of cells in that particular trace. (**H**) Quantitation of MTP (10 μM) induced ER Ca^{2+} stores depletion before and after the addition of 2 μM Tg. 620 and 539 cells from 9 and 7 independent imaging dishes were analyzed for the two conditions, respectively. (**I**) Quantitation of Tg, (2 μM) induced ER Ca^{2+} stores depletion before and after the addition of 10 μM MTP. 539 and 620 cells from 7 and 9 independent imaging dishes were analyzed for the two conditions, respectively ("$n = x, y$" where "x" denotes total number of cells imaged and "y" denotes number of traces recorded). Data presented are mean ± S.E.M., unpaired Student's t test, $^{**}P < 0.01$; $^{***}P < 0.001$.

© The Author(s)

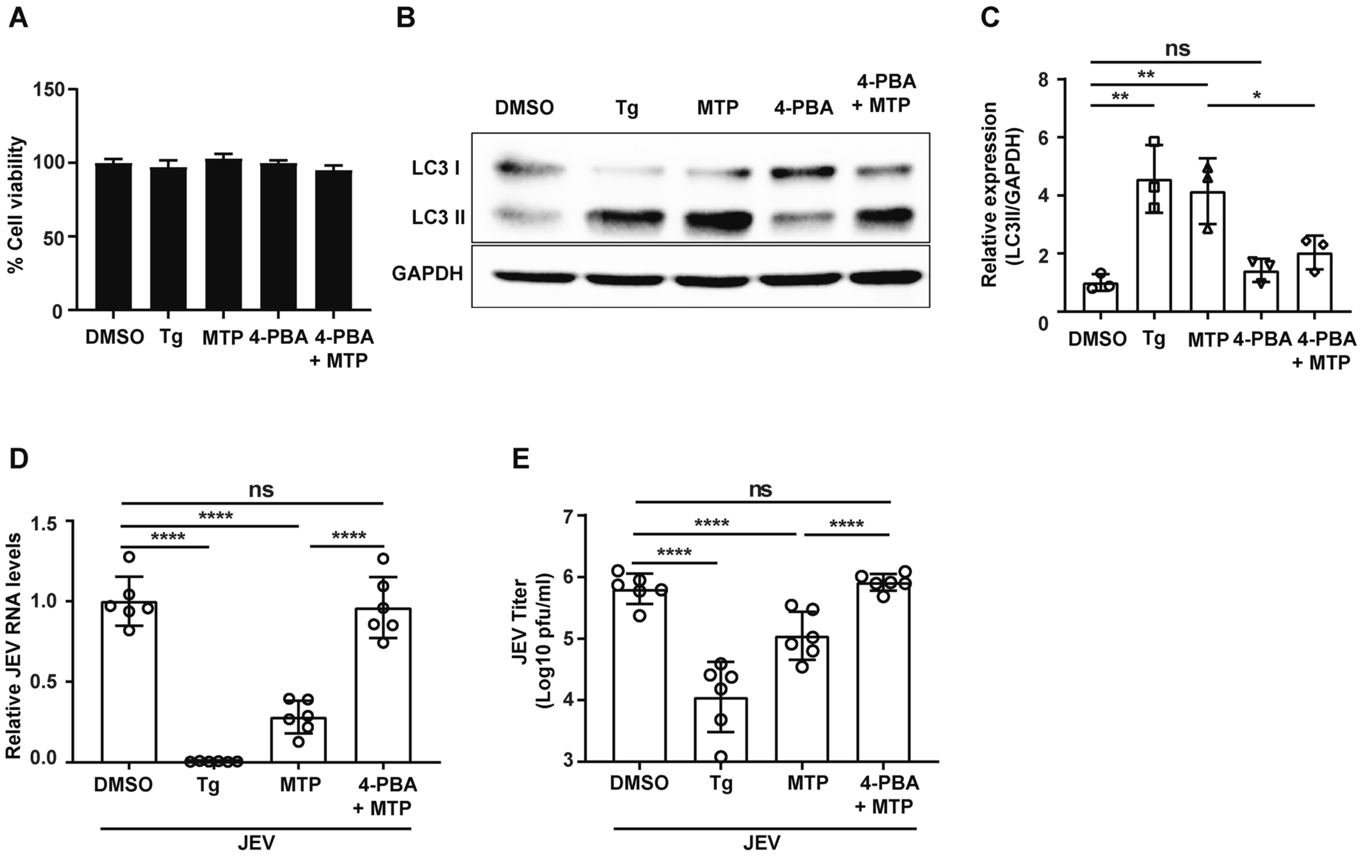

Figure EV4. MTP induced ER stress is essential for autophagy and antiviral effect.

(**A**) Neuro2a cells were treated with DMSO/Tg (1 μM)/MTP (10 μM)/4-PBA (2 mM)/4-PBA (2 mM) + MTP (10 μM) for 24 h. MTT assay was used to calculate % cell viability, and normalized to DMSO treated control ($n = 3$). (**B**,**C**) Neuro2a cells were treated with DMSO/Tg (1 μM)/MTP (10 μM)/4-PBA (2 mM)/4-PBA (2 mM) + MTP (10 μM) for 6 h. (**B**) Protein lysates were analyzed by immunoblotting using LC3 and GAPDH (loading control) antibodies. (**C**) Bar-graph shows relative protein expression level of LC3II/GAPDH calculated after normalization to DMSO control. Values were plotted from three independent experiments. (**D**,**E**) Neuro2a cells were infected with JEV at MOI 1 for 1 h. Post-infection, cells were treated with DMSO/Tg (1 μM)/MTP (10 μM)/4-PBA (2 mM) + MTP (10 μM) for 24 h. (**D**) Cells were harvested, viral transcript levels were measured using qRT-PCR and normalized to DMSO-treated infected control from two independent experiments ($n = 6$). (**E**) Virus titers was measured in culture supernatant using plaque assay, value plotted from two independent experiments ($n = 6$). Data information: All data were expressed as means ± SD, unpaired Student t-test was used to calculate statistical significance *$P < 0.05$; **$P < 0.01$; ***$P < 0.001$; ****$P < 0.0001$; ns, not significant.

© The Author(s)

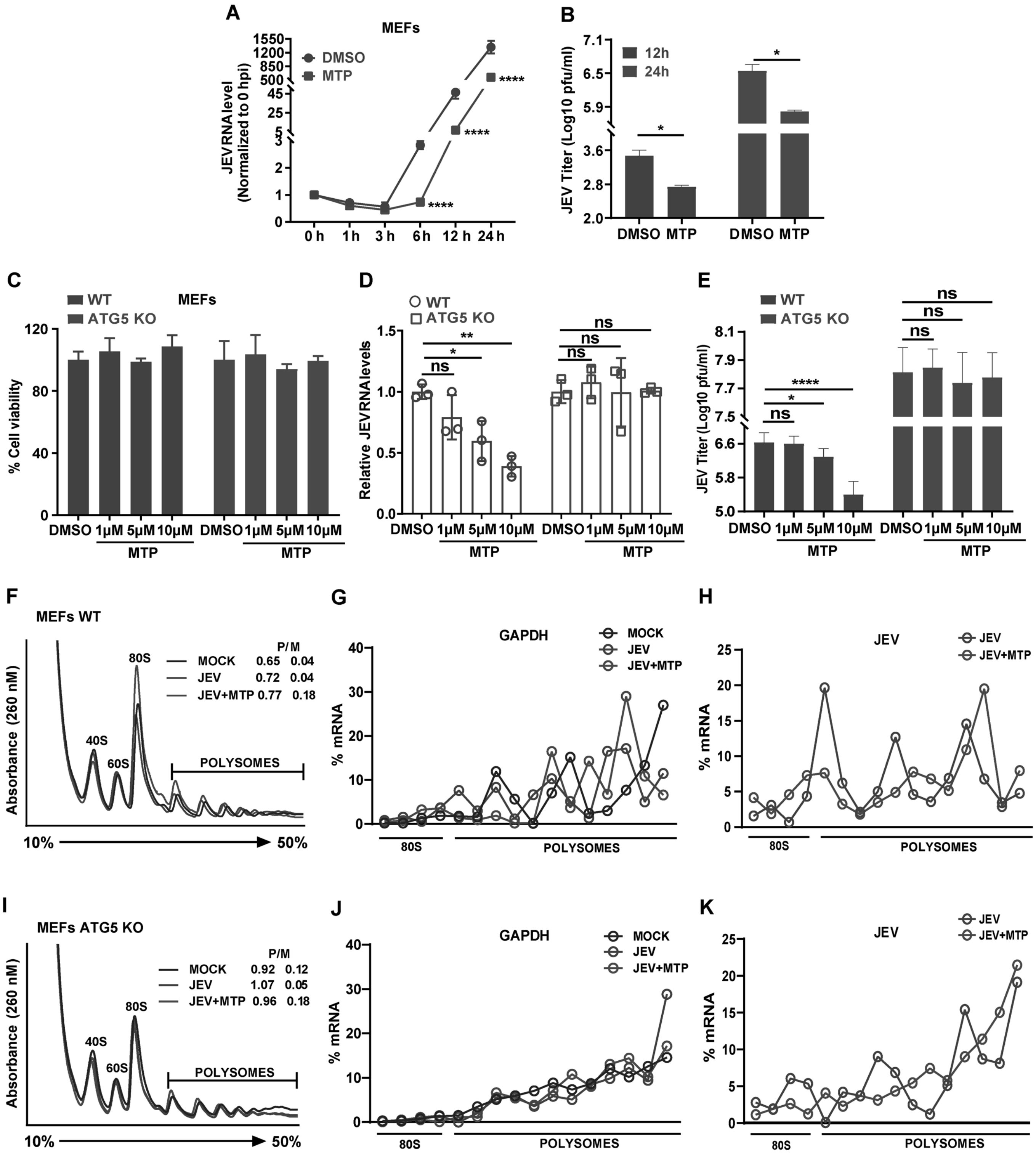
A
MEFs
DMSO
MTP
JEV RNA level (Normalized to 0 hpi)
0 h 1 h 3 h 6 h 12 h 24 h
B
12h
24h
JEV Titer (Log10 pfu/ml)
DMSO MTP DMSO MTP
C
WT
ATG5 KO
MEFs
% Cell viability
DMSO 1µM 5µM 10µM DMSO 1µM 5µM 10µM
MTP
D
Relative JEV RNA levels
E
JEV Titer (Log10 pfu/ml)
F
MEFs WT
Absorbance (260 nM)
40S
60S
80S
POLYSOMES
P/M
MOCK 0.65 0.04
JEV 0.72 0.04
JEV+MTP 0.77 0.18
10%
50%
G
GAPDH
MOCK
JEV
JEV+MTP
% mRNA
80S
POLYSOMES
H
JEV
I
MEFs ATG5 KO
MOCK 0.92 0.12
JEV 1.07 0.05
JEV+MTP 0.96 0.18
J
K

Figure EV5. Antiviral effect of phenothiazines is autophagy dependent.

(**A**,**B**) MEFs were mock/JEV infected (MOI 1) and at 1 hpi, treated with DMSO/MTP (10 µM). (**A**) Cells were harvested at the indicated hpi and viral RNA levels were quantified using qRT-PCR. Data represents values obtained from two independent experiments ($n = 6$). (**B**) Culture supernatant was used to determine virus titers using plaque assay. Data is plotted from two independent experiments ($n = 6$), and compared by unpaired Student t-test. (**C**) WT and ATG5 KO MEFs were treated with indicated concentrations of MTP for 24 h, and the percentage cell viability was measured and normalized to respective DMSO-treated controls ($n = 3$). (**D**,**E**) WT and ATG5 KO MEFs were infected with JEV at MOI 1, and at 1 hpi treated with MTP at indicated concentrations. Cells were harvested at 24 hpi and the relative viral RNA levels were quantitated using qRT-PCR, and plotted after normalization to respective DMSO-treated control. Data represents values from three independent experiments ($n = 9$). (**E**) Culture supernatant was collected and virus titers was determined using plaque assay. Data is plotted from three independent experiments ($n = 9$). (**F**–**K**) WT and ATG5 KO MEFs were mock/JEV (5 MOI) infected for 1 h, followed by DMSO/MTP (10 µm) treatment till 6 hpi. (**F**,**I**) Global polysome profile analysis of cell lysates were performed by the density gradient fractionation system. Polysome-to-monosome (P/M) ratios from two independent experiments, means ± SD. (**G**,**H**,**J**,**K**) Percentage distribution of GAPDH mRNA (housekeeping gene) (**G**,**J**), viral RNA (**H**,**K**) in the monosome and polysome fractions was analyzed by qRT-PCR. Similar trends were seen in two independent experiments. Data information: One-way ANOVA followed by Dunnett test was used for the determination of statistical significance, *$P < 0.05$; **$P < 0.01$; ****$P < 0.0001$, ns, non-significant.

© The Author(s)
